# Evolution of the expression and regulation of the nuclear hormone receptor *ERR* gene family in the chordate lineage

**DOI:** 10.1016/j.ydbio.2023.09.003

**Published:** 2023-09-09

**Authors:** Vasileios Papadogiannis, Dorit Hockman, Silvia Mercurio, Claire Ramsay, Mark Hintze, Cedric Patthey, Andrea Streit, Sebastian M. Shimeld

**Affiliations:** 1 Department of Biology, University of Oxford, 11a Mansfield Road, Oxford OX1 3SZ; 2 Division of Cell Biology, Department of Human Biology, Faculty of Health Sciences, University of Cape Town, Cape Town, RSA; 3 Neuroscience Institute, Faculty of Health Sciences, University of Cape Town, Cape Town, RSA; 4 Department of Environmental Science and Policy, Università degli Studi di Milano, via Celoria 2 20133 Milano; 5 Centre for Craniofacial & Regenerative Biology, Faculty of Dentistry, Oral and Craniofacial Sciences, King’s College London, London UK; 6 Department of Radiosciences, Umeå University, 901 85 Umeå, Sweden

## Abstract

The *Estrogen Related Receptor* (*ERR*) nuclear hormone receptor genes have a wide diversity of roles in vertebrate development. In embryos, ERR genes are expressed in several tissues, including the central and peripheral nervous systems. Here we seek to establish the evolutionary history of chordate *ERR* genes, their expression and their regulation. We examine *ERR* expression in mollusc, amphioxus and sea squirt embryos, finding the single *ERR* orthologue is expressed in the nervous system in all three, with muscle expression also found in the two chordates. We show that most jawed vertebrates and lampreys have four *ERR* paralogues, and that vertebrate *ERR* genes were ancestrally linked to *Estrogen Receptor* genes. One of the lamprey paralogues shares conserved expression domains with jawed vertebrate *ERRγ* in the embryonic vestibuloacoustic ganglion, eye, brain and spinal cord. Hypothesising that conserved expression derives from conserved regulation, we identify a suite of pan-vertebrate conserved non-coding sequences in *ERR* introns. We use transgenesis in lamprey and chicken embryos to show that these sequences are regulatory and drive reporter gene expression in the nervous system. Our data suggest an ancient association between *ERR* and the nervous system, including expression in cells associated with photosensation and mechanosensation. This includes the origin in the vertebrate common ancestor of a suite of regulatory elements in the 3’ introns that drove nervous system expression and have been conserved from this point onwards.

## Introduction

The *Estrogen Related Receptor* genes (*ERR*, also sometimes called *ESRR*) form a small family of transcription factors and encode Nuclear Hormone Receptor (NHR) type DNA binding proteins. There are typically four ERR family paralogues in jawed vertebrates, *ERRα, ERRβ, ERRγ* and *ERRδ*, though there is variation between lineages due to extra duplications and/or gene loss. Based on sequence similarity the ERR family is closely related to the Estrogen Receptor (ER) family (Baker, 2008; Bertrand et al., 2004), but there is an important difference in how they function: ER proteins require ligand-binding to activate transcription of target genes, while ERR is an ‘orphan’ NHR that does not need a ligand to bind DNA. ERR proteins act by binding to DNA sequences known as Estrogen Related Receptor Elements (ERREs) and by regulating the expression of target genes (Heard et al., 2000; Tremblay and Giguere, 2007).

The jawed vertebrate ERR genes have been quite well studied in several species, with embryonic expression identified in multiple tissues including Central and Peripheral Nervous Systems (CNS and PNS), muscle and kidney, with data from enough species to suggest that some and possibly all of these sites of expression could be ancestral for jawed vertebrates (Bardet et al., 2004; Heard et al., 2000; Susens et al., 2000). Our study was initiated following the identification of one ERR gene, *ERRγ*, as a marker for the neurons of the chicken vestibuloacoustic ganglion (VA) (Patthey et al., 2016). *ERRγ* is also expressed by VA cells in mouse and zebrafish, and the locus is associated with hearing loss in mouse mutants and human genetic conditions (Bardet et al., 2004; Bardet et al., 2005; Bertrand et al., 2007; Lorke et al., 2000; Nolan et al., 2013). These data suggest *ERRγ* functions in the specification of VA cells in a way that is conserved across jawed vertebrates. However, *ERRγ* expression has also been detected in some other cranial ganglia, including the trigeminal, and is widely expressed in the CNS as well (Bonnelye et al., 1997), complicating inference of the ancestral expression of *ERRγ* genes. This problem is compounded by limited data from invertebrates, with current studies restricted to the description of expression in the developing *Drosophila* gut deriving from a large-scale *in situ* hybridisation screen, expression in the larval head of tunicates, and expression in somites, CNS and PNS of amphioxus (Bardet et al., 2005; Gomes et al., 2019b; Park et al., 2009; Thurmond et al., 2019).

We studied the molecular evolution, expression and regulation of *ERR* genes in lamprey and in three invertebrates, a limpet and additional amphioxus and tunicate species to the ones already studied. Lampreys are members of the earliest-diverging extant vertebrate group. They lack hinged jaws and paired appendages but share many developmental processes and structures with other vertebrates (Green and Bronner, 2014; Shimeld and Donoghue, 2012). Evidence from broad genome comparison shows two rounds of whole genome duplication (2R) preceded the radiation of living jawed vertebrate lineages, with at least one of these predating separation of the lamprey lineage (Sacerdot et al., 2018; Simakov et al., 2020). Tunicates are the sister lineage to vertebrates and the immediate outgroup for comparison to vertebrates, while amphioxus gives insight into the ancestral chordate condition. Limpets are molluscs with motile planktonic embryos and larvae which may provide a better comparison than *Drosophila* to the embryos and larvae of tunicates and amphioxus.

We first report the evolutionary history of chordate *ERR* genes including the identification of four lamprey *ERR* genes. We establish *ERR* expression in mollusc, amphioxus, tunicate and lamprey embryos to obtain a broader picture of the evolution of ERR gene expression. One lamprey *ERR* gene, annotated as *ERRa*, shows highly similar expression to jawed vertebrate *ERRγ* in the CNS and VA, and we hypothesise this reflects a conserved, ancestral pattern for vertebrates. To examine this further we examine syntenic relationships between *ERR* genes, including identifying an ancient linkage to *ER* genes that probably reflects their origin through the tandem duplication of an *ERR/ER* ancestor gene. Furthermore, we search for conserved non-coding elements (CNEs) around *ERR* genes, identifying several embedded in the 3’ introns of jawed vertebrate *ERRγ* and lamprey *ERRa*. We test the function of one group of CNEs in transgenic lamprey and chicken, showing they drive expression in the CNS and weakly in the PNS. Our data demonstrate an ancient association between *ERR* genes and the nervous system, cemented in vertebrates with the origin and subsequent conservation of regulatory elements embedded in 3’ introns.

## Materials and Methods

### Gene and genome analyses

A reciprocal BLAST (Camacho et al., 2009) approach was used to identify cyclostome sequences. Transcriptomes for the brook lamprey (*Lampetra planeri)*, the arctic lamprey (*Lethenteron camtchaticum*) and the sea lamprey (*Petromyzon marinus*) (Lara-Ramirez et al., 2015; Mehta et al., 2013; Smith et al., 2018) were searched using jawed vertebrate sequences for each gene family. Transcript sequences matching the queries were searched via BLASTX against the NCBI RefSeq database to confirm they matched the expected gene family. Transcripts filtered through this process were subsequently mapped to the genomes of the arctic lamprey and the sea lamprey and then consolidated into individual genetic loci. Sequences for the inshore hagfish (*Eptatretus burgeri*), common limpet (*Patella vulgata*) and keelworm (*Spirobranchus lamarkii*) were identified via the same approach. For P. *vulgata* and *S. lamarckii* previously published transcriptome and genome data were used (Kenny et al., 2015; Kenny and Shimeld, 2012; Werner et al., 2013). Other sequences included in this analysis were retrieved from the NCBI database. Sequence alignments were built using MUSCLE and trimmed using TRIMAL with a gap threshold of 0.5. Maximum parsimony (raxml (Stamatakis, 2014)) and Bayesian (Mr Bayes (Altekar et al., 2004; Huelsenbeck and Ronquist, 2001; Ronquist and Huelsenbeck, 2003)) analysis were employed (JTT model, 1000 bootstrap) to construct phylogenetic trees. *ERRγ* synteny was investigated in the genomes of different annotated vertebrate species as listed in supplementary data. For jawed vertebrate genomes, the following genome assemblies were searched using the UCSC genome browser: *Homo sapiens* (GRCh38.p12, NCBI ID: 5800238), *Gallus gallus* (GRCg6a, NCBI ID: 1668981), *Xenopus tropicalis* (Xenopus_tropicalis_v9.1, NCBI ID: 768701), *Danio rerio* (Zebrafish Build 11/ GRCz11, NCBI ID: 1104621), *Lepisosteus oculatus* (LepOcu1, NCBI ID: 327908). Additionally, the following genomes were used to identify ERR-ER locations in different species shown in Figure 2: *Oryzias latipes* (UCSC oryLat2), *Anolis carolinensis* (AnoCar2.0, accession #GCA_000090745.1), *Branchiostoma floridae* (JGI v2.0), *Lottia gigantea* (JGI Lotgi1), *Saccoglossus kowalevskii* (Skow_1.1 NCBI ID: 95611). In cases where gene annotation was lacking, gene identity was confirmed via a reciprocal BLAST approach. New sequences for genes cloned as part of this study have been submitted to Genbank, accession numbers MN267867 to MN267870.

### Embryos and *in situ* hybridisation

Primers (Supplementary Table S2) were designed to amplify fragments 500-1000bp in length, when possible, from embryonic cDNA. *L. planeri* embryos were collected from the New Forest, UK, under licence from Forestry England, and staged as described (Tahara, 1988). Adult *C. intestinalis* adults were collected from Northney marina, Hayling Island, UK, and adult *P. vulgata* from Tinside, Plymouth, UK. Both were maintained in a running seawater aquarium at 12-16°C. Gametes were liberated by dissection and embryos were cultured at 16-18°C until they reached the desired stage. *B. lanceolatum* were collected from Banyuls-sur-Mer, France and heat stimulation was used to induce spawning (Fuentes et al., 2007; Fuentes et al., 2004).

*L. planeri, C. intestinalis* and *B. lanceolatum* embryos were fixed with 4% paraformaldehyde (PFA) and stored at -20°C after gradual transfer to Methanol (*L. planeri*) or Ethanol (invertebrate embryos). Lamprey *in situ* hybridisation was performed as described (Lara-Ramirez et al., 2015), *C. intestinalis* and *B. lanceolatum in situ* hybridisation was performed as described (Boorman and Shimeld, 2002). *P. vulgata in situ* hybridisation was performed as described (Shimeld et al., 2010). Scanning electron microscopy of *P. vulgata* embryos was performed as described (Thompson and Shimeld, 2015). Lamprey embryo clearing was either by transferring to 25% then 50% glycerol in PBS, or by dehydrating with 25%, 50%, 75% and 100% ethanol and transferring to benzyl benzoate/benzyl alcohol 2:1 (BABB). Differential Interference Contrast (DIC) microscopy was used to observe and photograph embryos. The *Ciona ERR* probe was derived from the *Ciona* release 1 gene collection library, clone coordinate R1CiGC03o05 (gene model KH2012.L8.21).

Adult sea lamprey (*Petromyzon marinus*) were supplied by the US Fish and Wildlife Service and Department of the Interior. Embryos obtained by *in vitro* fertilisation were grown to the desired stage in compliance with California Institute of Technology Institutional Animal Care and Use Committee protocol #1436.

### CNE cloning and transgenics

Human and Zebrafish CNE sequences from the Conserved Non-coDing Orthologous Regions (CONDOR: (Woolfe et al., 2007)) database were searched against the *Lethenteron camtchaticum* genome using BLAST, providing evidence that shared CNEs were present between the 3’ most introns of *ERRγ* and *ERR*a. The last two introns of *L. camtchaticum ERRa* and *ERR*γ from Human, Mouse, Chicken, *Anolis, Xenopus*, Zebrafish and elephant shark were subsequently aligned using BLAST, and VISTA plots were built using mVISTA (Frazer et al., 2004) to reveal conservation peaks. Sequences in each identified peak were extracted for each species and alignments were built using MUSCLE.

CNEs were cloned from the second to last intron of *ERRa* from *L. planeri* genomic DNA. CNE-containing fragments were cloned using the InFusion system (Takara Biosciences) into respective GFP reporter vector for testing in chicken (barcode 1 tagged pTK vector (Chen and Streit, 2015)) and lamprey (Parker et al., 2014). More specifically, for lamprey transgenics, CNE sequences were cloned into the HLC vector with a zebrafish *krt4* minimal promoter (Parker et al., 2014). *ERR8t12* was first cloned in the pCR™II vector and then transferred to the krt4-HLC vector after restriction digest of both with BamHI-XhoI and ligation. For chicken transgenics, *ERR8t12* was cloned from pCR™II to the tag1-pTK vector using restriction-ligation cloning with Spe1-ECORV. For the chicken transgenics, in-ovo electroporations were performed as described (Abello et al., 2007), targeting the otic region at stage HH10 and allowing embryos to grow to HH15-HH17 and HH21. The *ERR8t12* element was cloned into a modified form of pTK-EGFP (Chen and Streit, 2015). A control plasmid with a ubiquitous promoter driving DsRed was co-electroporated with the GFP reporter constructs to visualise all transgenic cells. For lamprey transgenics, i-SceI meganuclease integration was used in sea lamprey embryos as described (Parker et al., 2011), injecting zygotes and allowing embryos to grow to Tahara stage 26-29. Embryos of all three species were imaged live then fixed for 1 hour in 4% PFA and transferred to PBS-TritonX100 for storage. For sectioning chicken embryos were embedded in gelatin then sectioned with a cryostat.

### Immunohistochemistry

Fixed embryos were initially washed 3 times in PBS-TritonX100 (PBS with 0.5% Triton X100) for 30 minutes, washed 2 times in block solution (PBS-TritonX100 with 10% heat-treated sheep serum) for 30 minutes and blocked in block solution for longer than 2 hours, and up to overnight. Embryos were then incubated overnight with the primary antibody solution followed by 5 PBS-TritonX100 and 2 block solution washes for 30 minutes. Secondary antibody solution incubation was then carried out for longer than 4 hours up to overnight, followed by 5 PBS-TritonX100 washes for 30 minutes each. Embryos were finally transferred to 25% and then 50% glycerol in PBS to clear and visualised in an optical fluorescent (Zeiss Axioskop) or confocal (Olympus FV1000) microscope. Sections were initially incubated in PBS at 45°C for 30 minutes to remove gelatin. Then 5 washes in PBS-TritonX100 for 15 minutes were followed by blocking for 1 hour at 4°C and overnight incubation in primary antibody, 5 washes in PBS-TritonX100 for 15 minutes, incubation with secondary antibody for more than 2 hours and 5 washes in PBS-TritonX100 for 15 minutes before transferring to glycerol and sealing. Antibodies used were: Chicken Polyclonal Anti-GFP antibody (Abcam AB13970), Goat Anti-Chicken IgY H&L Alexa Fluor® 488 (Abcam AB150169), Mouse Hu/ELAV Monoclonal Antibody (Invitrogen 16A11), anti-Mouse alexa594 (Abcam AB150116), mouse anti-GFP (Invitrogen A-11120) (used in chicken) visualised with Goat anti-Mouse IgG H+L Alexa Fluor® 488 (Invitrogen A-11001).

### Image analysis

Analysis and processing of fluorescence microscopy data were carried out using ImageJ v. 1.52g. Confocal or optical z stacks and 3D projections were created using maximum intensity projection. Some images are composites of multiple individual images of the same embryo focused at slightly different levels to follow expressing cell populations as the embryo moved in and out of focus. These were assembled in PowerPoint by overlapping individual images. This applies to images shown in Figure 7A, B, G, and H.

### Transcriptome assembly and analysis

RNA-seq data from dissected eyes from lamprey, hagfish, gar and shark (Lamb et al., 2016) were downloaded from the NCBI (Bioproject PRJNA292033). Libraries for each species were separately assembled using Trinity with the integrated Trimmomatic option for trimming (Haas et al., 2013). These assemblies were then searched for ERR genes using BLAST. Read counts for each transcript were then generated using Kalisto (Bray et al., 2016).

### Single-cell RNAseq data analysis

*Ciona robusta* and amphioxus single-cell RNAseq (scRNAseq) data have been previously published (Cao et al., 2019; Ma et al., 2022). Data were accessed at https://singlecell.broadinstitute.org/single_cell/study/SCP454/comprehensive-single-cell-transcriptome-lineages-of-a-proto-vertebrate and https://lifeomics.shinyapps.io/shinyappmulti/ respectively. In each case, we first used the website interface to search for the profiles of selected genes across cell clusters. For the *Ciona robusta* data we were able to identify two well-defined neural cell clusters expressing *ERR* (gene ID KH2012: KH.L8.21). To gain insight into the nature of *ERR* expressing cells we extracted the lists of genes significantly enriched in these cell clusters (as defined by the original scRNAseq study (Cao et al., 2019)), and retrieved Gene Ontology (GO) terms for *C. robusta* genes from the Aniseed database (https://www.aniseed.fr/: (Brozovic et al., 2018)). These data were then analysed with g:Profiler (Raudvere et al., 2019) to identify functional enrichment with a threshold of 0.05. We also directly searched genes in these cell clusters for those annotated with GO terms related to photoreception.

## Results

### Evolutionary history of the *ERR* gene family

**Figure 1:**
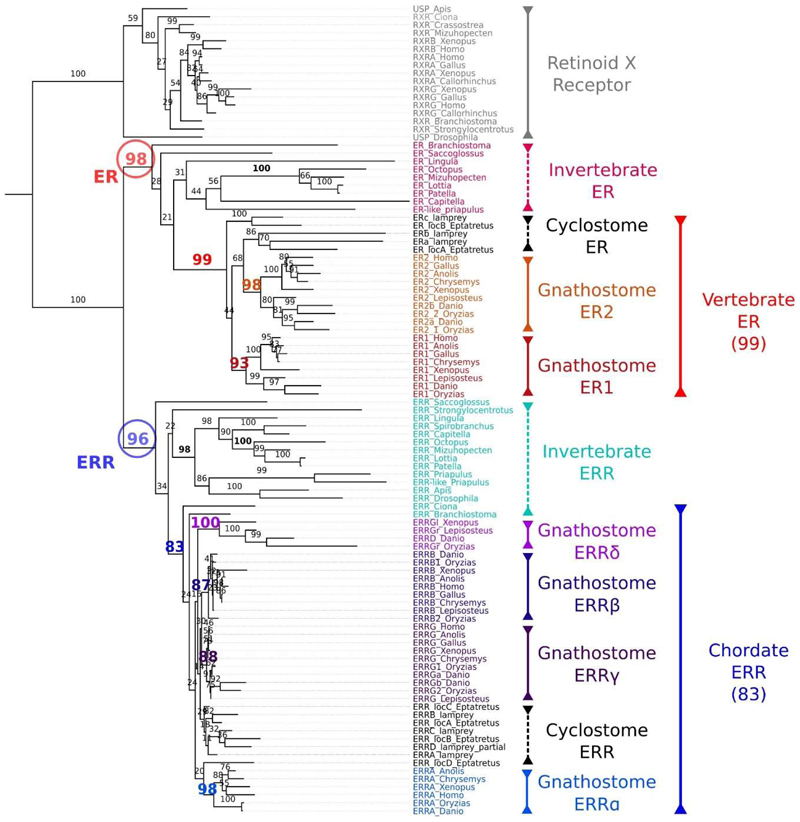
Molecular phylogenetic analysis of *ERR* and *ER* sequences Phylogenetic tree using maximum likelihood analysis with 1000 bootstrap replicates (showing as percentage support values on nodes) of ERR and ER family members. The RXR family is used as an outgroup. Different taxonomic groups are colour coded and highlighted with bars on the right side, with continuous lines indicating monophyly and dashed lines indicating non-monophyletic groups. All sequences described in this study fall robustly within their expected families: ER (98%, red circle), ERR (96%, blue circle), RXR (100%). Bootstrap support values for chordate, vertebrate and gnathostome clades are coloured according to the respective group name colour, other node support values have been omitted. *ERRδ* is also known as *ERRγr* or *ERRγl* depending on species. Lamprey sequences are from *Lethenteron camtschaticum*.

We first sought to describe the evolutionary history of chordate *ERR* genes. We identified *ER* and *ERR* genes from three lamprey species (*Lampetra planeri, Petromyzon marinus, Lethenteron camtschaticum*), one hagfish species (*Eptatretus burgeri*) and a range of other vertebrate and invertebrate taxa. Molecular phylogenetic analysis of encoded protein sequences using the Retinoid X Receptors as outgroups (Mangelsdorf et al., 1992) showed ER and ERR sequences formed well supported groups, each including vertebrate and invertebrate sequences (Fig. 1). Most invertebrates (including the invertebrate chordates) had a single *ERR* gene, while lampreys and hagfishes had four *ERR* genes. As expected, jawed vertebrate ER (ER1, ER2) and ERR paralog sequences formed separate well-supported groups. Lamprey and hagfish ERR and ER sequences grouped within the ERR and ER families respectively. However, while lamprey and hagfish sequences generally grouped, the tree did not resolve sufficiently to determine the orthology between lamprey and jawed vertebrate sequences.

**Figure 2:**
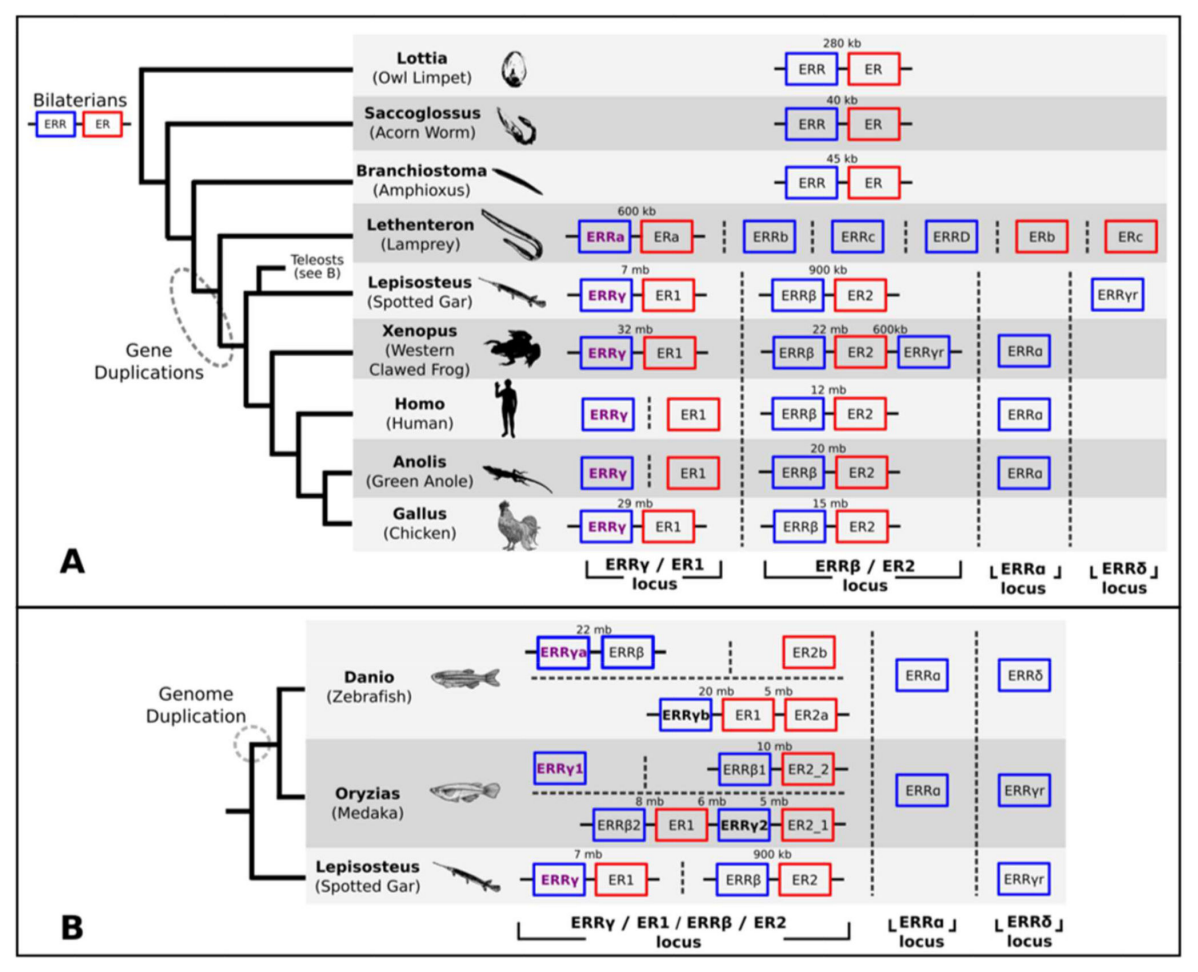
The evolutionary history of *ERR-ER* gene linkage *ERR* genes are in blue and *ER* genes in red. A black line connecting the gene boxes indicates the loci lie in proximity in the same scaffold/chromosome, with intergenic distances shown. Dotted vertical lines represent a change in scaffold/chromosome. Phylogenetic relationships of species presented are on the left. A shows the broader context across animals and spanning the 2R genome duplications. B is focused on the ray-finned fish, amongst which teleosts have undergone another genome duplication. Pictures from https://commons.wikimedia.org/w/index.php?title=Main_Page&oldid=343113363.

While extracting lamprey ERR and ER sequences, we noted one *ERR* paralogue and one *ER* paralogue were about 600kb apart in the same genomic scaffold. This prompted us to examine *ERR* and *ER* gene localisation in other genomes, identifying additional *ERR-ER* gene linkages (Fig. 2A, B). The distance between *ERR* and *ER* genes is small in some genomes, for example around 40Kb in the hemichordate *Saccoglossus kowalevskii*, but in jawed vertebrates is usually in the order of several Mb. Vertebrate paralogue pairs *ERRγ-ER1* and *ERRβ-ER2* often appear in linkage, while the *ERRα* and *ERRδ* loci appear unlinked to ER loci in all jawed vertebrates examined, except for *Xenopus* where *ERRδ* is found close to *ER2* (Fig. 2).

### *ERR gene* expression in tunicate, amphioxus and mollusc embryos

**Figure 3:**
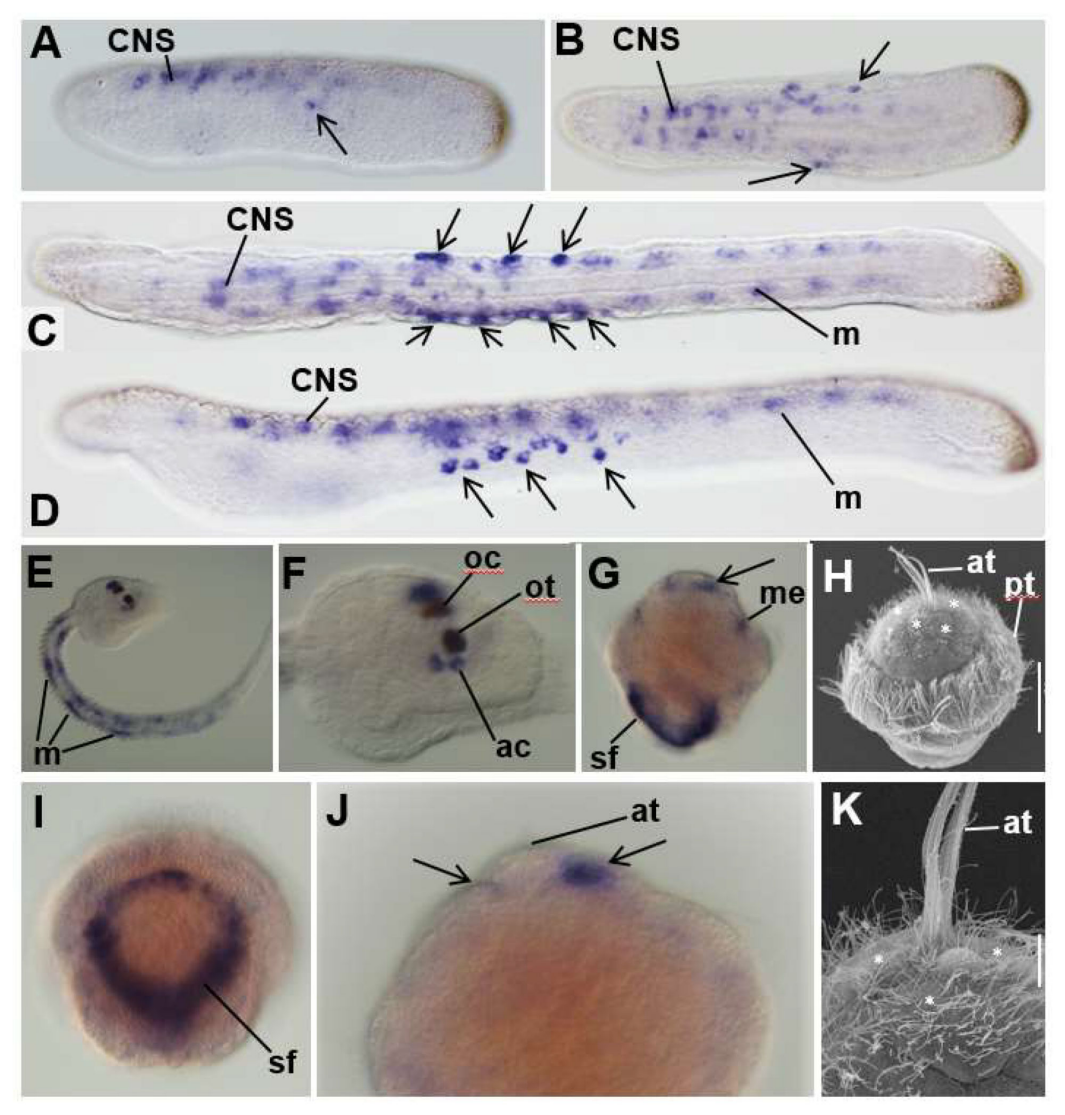
*ERR* expression in amphioxus, ascidian and mollusc embryos. A-D. Amphioxus (*Branchiostoma lanceolatum*) 32hr embryo (A,B) and early (42hr) larva (C,D) in lateral (A,D) and dorsal (B,C) views, anterior to the left. Expression is seen in the neural tube (CNS) and PNS (arrows), as well as in muscle (m) in the larva. E-F. *Ciona* late tailbud stage, F is a close up of the head of the specimen in E. Expression is seen in muscle and in ocellus (oc) and antenna cells (ac) by the otolith (ot) in the head. G. Limpet trochophore showing expression around the shell field (sf) and some anterior mesoderm (me) as well as in cells by the apical tuft (arrows). Close ups of the shell field and apical tuft (at) in different embryos are shown in I and J. Arrows in J point to expressing cells by the apical tuft. H, K, SEMs of limpet trochophores at the same stage, focused on the apical tuft and the surrounding ciliated cells which are marked with white asterisks and some of which express *ERR* (compare to G and J, respectively). Additional abbreviation: pt, prototroch. Scale bar on H is 50μm, on K is 10μm.

Previous studies of embryonic *ERR* spatial expression in invertebrates are limited to *Drosophila* (developed as part of high throughput *in situ* hybridisation screening (Thurmond et al., 2019)), the amphioxus *Branchiostoma floridae* (Bardet et al., 2005) and the larval head of an ascidian, *Phallusia mammillata* (Gomes et al., 2019a; Gomes et al., 2019b). Since these data were insufficient for useful comparison to vertebrates, we examined embryonic gene expression in additional invertebrates: a second amphioxus species, (*Branchiostoma lanceolatum*), another tunicate (*Ciona intestinalis*) and a gastropod mollusc (the common limpet *Patella vulgata*). *ERR* expression in *B. lanceolatum* localised to both CNS and PNS, and to axial muscles (Fig. 3A-D), essentially as reported in *B. floridae* (Bardet et al., 2005). As seen in *B. floridae*, expression in the PNS was confined to a subset of PNS cells. We also searched published scRNAseq data from amphioxus (Ma et al., 2022). This identified expression in mesoderm and neural tissue (Supplementary Figure S1) but was insufficient to identify specific PNS cell types.

In *C. intestinalis ERR* was expressed in axial muscles and in the CNS, in a structure called the sensory vesicle (Fig. 3E, F). Sensory vesicle cells expressing *ERR* form part of the ocellus (the visual organ of the larva) and also include two cells in the lining of the vesicle in which the otolith, the larval inertial/gravity sensor, lies. From their position, we considered that these cells are possibly those known as the ‘antenna cells’, which connect the otolith to the brain, probably relaying information about its physical displacement and thus allowing inertial sensing (Bostwick et al., 2020; Esposito et al., 2015; Ryan et al., 2017). *Ciona spcs*. also have several types of PNS cells at the stages we examined (Liu and Satou, 2020), but we did not detect *ERR* expression in any of these by *in situ* hybridisation.

To gain additional insight into the cell types expressing *ERR* we mined scRNAseq data from *Ciona robusta* (Cao et al., 2019; Horie et al., 2018b). The *Ciona robusta ERR* gene (KH2012:KH.L8.21) showed no evidence of expression in peripheral neural cell types, confirming in situ hybridisation results. However, *ERR* was prominently expressed in two central neural cell clusters, ‘Rx+ anterior sensory vesicle (Rx+ aSV)’ cells and ‘Lox5+ anterior sensory vesicle (Lox5+ aSV)’ cells (here we use the names given to these clusters by the authors of the scRNAseq study) (Supplementary Figure S1). Rx+ aSV cells express the *Rx* (*Retinal homeobox*) gene and are hence likely to be visual system cells (Mathers et al., 1997). To examine this, we extracted the list of genes significantly expressed in the Rx+ aSV cluster and assessed GO term enrichment, finding terms related to photosensation to be significantly enriched in this gene set (25 genes including an Opsin gene: Supplementary Figure S1). This indicates these cells are likely to be the *ERR* expressing cells adjacent to the ocellus, in turn implying the Lox5+ aSV cells may be the cells adjacent to the otolith. Genes significantly expressed in the Lox5+ aSV cells did not show informative GO term enrichment, though do include several genes with visual system GO annotations (19 genes, 8 of which overlap with the 25 genes from the Rx+ aSV cluster analysis: Supplementary Figure S1). If these cells are antenna cells, they should also express the vesicular glutamate transporter *VGlut* (KH2012:KH.C3.324) (Bostwick et al., 2020; Kourakis et al., 2021) while Rx+ aSV cells should not. The scRNAseq data confirmed this was the case (Supplementary Figure S1).

In *P. vulgata ERR* was expressed in trochophore larvae by cells of the mantle edge surrounding the shell field, and by cells of the apical organ (Fig. 3G, I, J). The apical organ is a ciliated sensory organ with a central tuft of long cilia, and comparison to scanning electron microscope images of embryos at the same developmental stage (Fig. 3H, K) shows expression is not in the central cells with elongated cilia, but in cells with shorter cilia lying next to them.

### *ERR gene* expression in lamprey embryos

**Figure 4:**
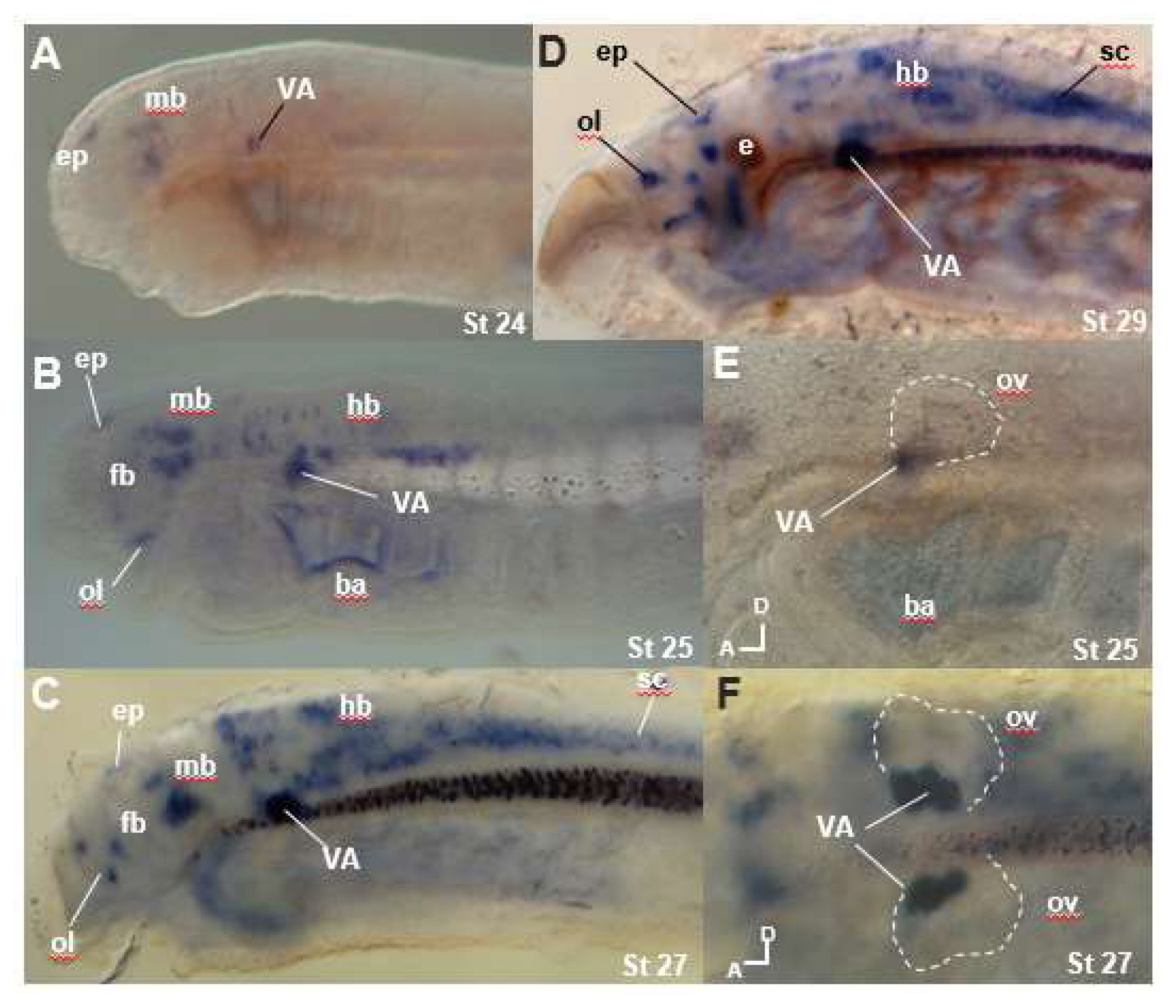
Lamprey *ERRa* Expression Embryos are in left lateral view with anterior to the right, except F which is a dorsal view. A. Lamprey *ERRa* expression at stage 24 in the epiphysis (ep), midbrain (mb) and vestibuloacoustic ganglion (VA). B. At stage 25, expression is also seen in in the ventral and dorsal hindbrain (hb), olfactory (ol) and forebrain (fb). Apparent labelling in the branchial arches (ba) in this specimen is background due to trapping. C. At stage 27 expression in the spinal cord (sc) is also visible. VA expression is very strong by this stage. D. Expression at stage 29 persists in all previous domains. E. Close-up of the otic region in a stage 25 embryo showing staining in the VA, positioned at the ventral and anterior side of the otic vesicle (ov; white dotted outline). F. Stage 27 embryo in dorsal view and focused on the VA region, white dotted outline marks the position of the otic vesicles. Additional abbreviations: e, eye.

We named the four lamprey *ERR* genes *ERRa* to *ERRd* to avoid direct attribution to jawed vertebrate *ERRα* to *ERRδ*, since this was not supported by molecular phylogenetic analysis. Sequences for *ERRa, ERRb* and *ERRc* were retrieved from embryo transcriptome data for three lamprey species and cloned from *L. planeri* embryo mRNA by reverse transcription PCR (RT-PCR). *ERRd* was identified in the *P. marinus* and *L. camtschaticum* genomes (Mehta et al., 2013; Smith et al., 2018), but was not present in the embryo transcriptomes and we were unable to amplify this gene from *L. planeri* embryo mRNA by RT-PCR. *In situ* hybridisation showed *ERRc* was only expressed in the hindbrain and part of the mandibular arch (Supplementary Figure S2), while embryonic expression of *ERRb* was not detected by this method. It also showed *ERRa* expression in the CNS including the midbrain, hindbrain, epiphysis, olfactory epithelium and spinal cord, as well as in the VA (Fig. 4). *ERRa* expression commenced at Tahara stage 24 (Tahara, 1988) in the epiphysis, midbrain and the VA (Fig. 4A). By stage 25 expression was also seen in the olfactory system, forebrain, hindbrain and in the spinal cord (Fig. 4B). From stage 27 onwards, expression persisted in all these domains, but with more widespread and stronger signal in the hindbrain and spinal cord (Fig. 4C). Expression in some cells in the eye could also be seen from stage 28 onwards (Fig. 4D, Supplementary Figure S3). Lamprey eye development is delayed relative to other vertebrates, with full vision only developing at metamorphosis after some years as a larva. To see if *ERR* expression persisted we examined published RNA-seq data from adult lamprey (*Geotria australis, Mordacia mordax*) and hagfish (*Eptatretus cirrhatus*) eyes (Lamb et al., 2016). We assembled transcriptomes for each species and searched for transcripts coded by *ERR* loci using reciprocal BLAST matches to *P. marinus* transcripts, identifying *ERRa, ERRb* and *ERRc* transcripts in all three species and *ERRd* in two species. Based on transcript per million (TPM) scores, we concluded that *ERRa, ERRb* and *ERRc* transcripts are expressed in adult lamprey and hagfish eyes (Supplementary Table S1).

### Lamprey *ERRa* and jawed vertebrate *ERRγ* share conserved synteny and non-coding elements driving CNS and PNS expression

**Figure 5:**
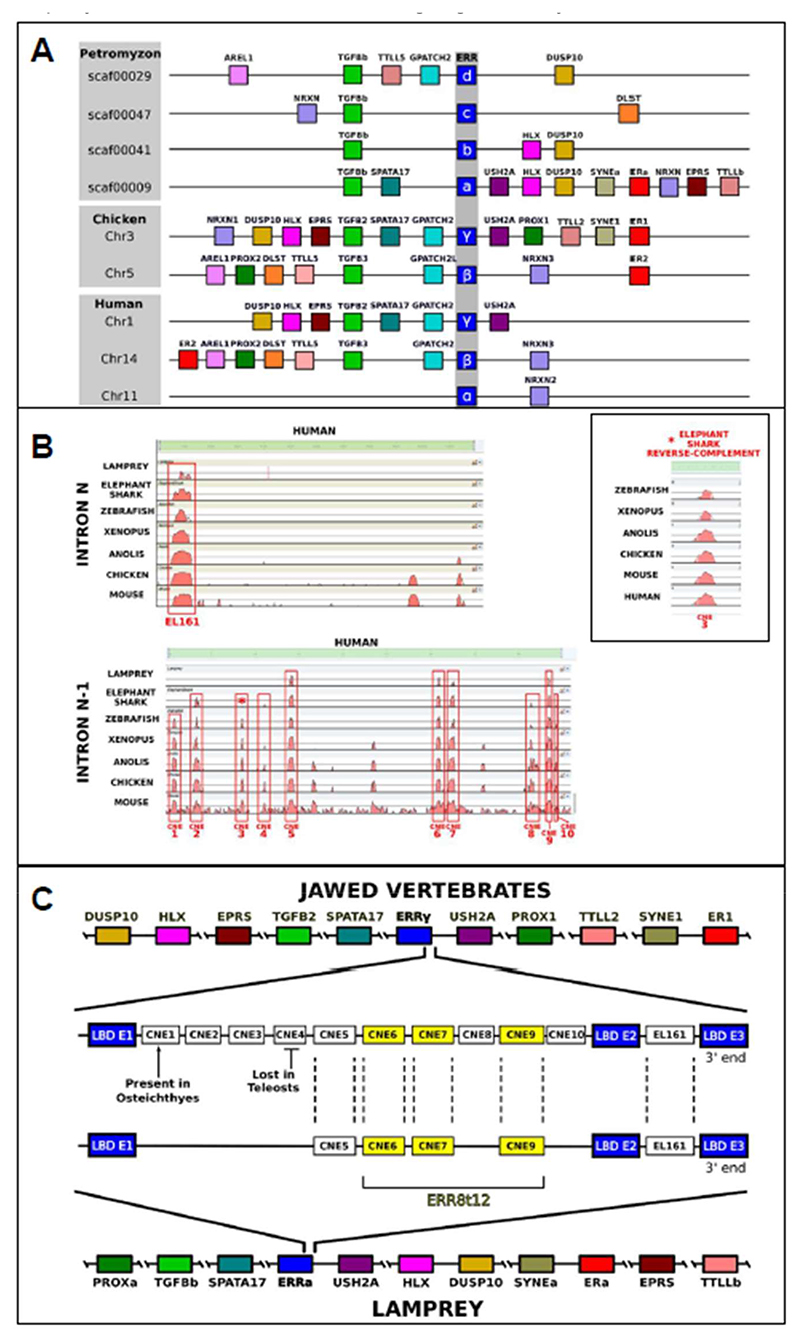
*ERRa-ERRγ* synteny and shared CNEs A. Local synteny around ERR loci in lamprey, chicken and human. The windows show a 1.5mb region for lamprey, a 6mb region for human and a 42mb window in chicken. Paralogues are colour coded. Only genes shared between loci are shown. Distances are not to scale, but gene order is as observed. B. Vista comparisons of the last and second-to-last introns of human ERR*γ* (N and N-1 respectively) showing conservation across vertebrate genomes and identification of CNEs. The inset shows a similar analysis using the putative shark CNE3 sequence in reverse orientation. C. schematic representation of CNEs also identified in lamprey. The ERR8t12 cluster is highlighted in yellow

It is notable that while lamprey *ERRa* and jawed vertebrate *ERRγ* do not appear as orthologues in our molecular phylogenetic analysis they share similarity in expression in the brain, spinal cord and VA. Synteny can provide parallel evidence to molecular phylogenetics for gene relationships between species. We compared neighbouring genes between lamprey and jawed vertebrate *ERR* loci, revealing they all evolved by block duplication of a single ancestral *ERR* locus (Fig. 5A; Supplementary Figure S4). This is consistent with the origin of vertebrate *ERR* paralogues by chromosome and genome scale duplications proposed to have occurred early in vertebrate evolution (Putnam et al., 2008; Simakov et al., 2020). Synteny was most similar between lamprey *ERRa* and jawed vertebrate *ERRγ*, with both flanked by the single copy genes *EPRS, SPATA17* and *USH2A*. This suggests that although *ERRa* and *ERRγ* do not resolve as orthologues in molecular phylogenetic analysis, they are sited in orthologous loci. Their similar expression prompted us to ask if they might also have shared regulatory CNEs leading to such conserved gene expression. A CNE named *EL161* shared across jawed vertebrates has been previously identified in the last intron of *ERRγ* and shown to be capable of regulating part of the *ERRγ* expression pattern in transgenic zebrafish (Roberts et al., 2014). Using Vista we identified this CNE in the last intron of lamprey *ERRa*, showing it to be ancestral for vertebrates (Fig. 5B). Our Vista comparison also identified additional CNEs (CNEs1-10) in the second to last intron of *ERRγ* (Fig. 5B). Four are conserved in lamprey *ERRa* and hence also ancestral for all living vertebrates (Fig. 5C). We also searched ascidian, amphioxus and mollusc *ERR* loci for these CNEs but did not find the conserved sequence in the orthologous introns, or elsewhere. Identifiable sequence conservation hence appears vertebrate-specific. CNE sequences and alignments can be found in the supplementary appendix.

**Figure 6:**
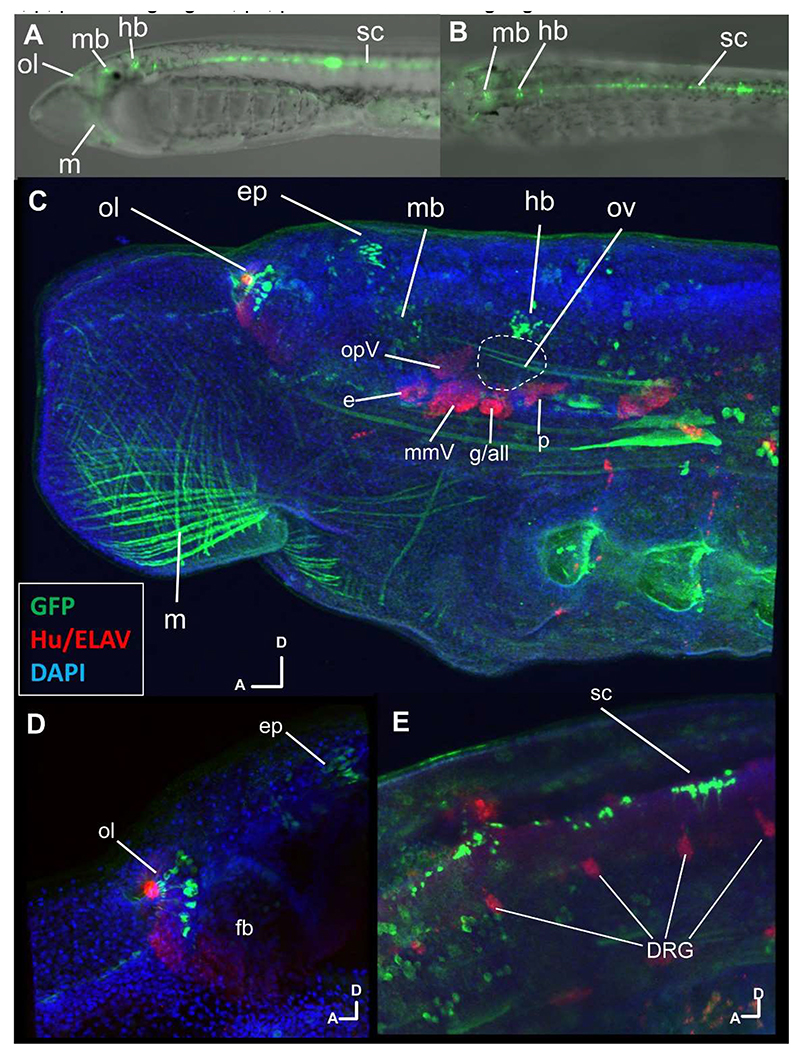
*ERR8t12* CNE activity in lamprey embryos A, B. Live stage 29 embryo in lateral and dorsal view respectively, showing reporter expression in olfactory system (ol), midbrain (mb), hindbrain (hb) and spinal cord (sc), as well as head muscle (m). For additional images see Supplementary Figure S5. C-E. Confocal reconstructions labelled for GFP (reporter, green), Hu/ELAV (differentiated neurons, red) and DAPI (nuclei, blue). Embryo orientations are shown in each image (A, anterior, D, dorsal). C. Stage 29 head showing reporter localisation in olfactory, epiphysis, midbrain (mb) and hindbrain, as well as cranial muscle. The otic vesicle is outlined, and cranial ganglia identified. D. close up of the olfactory and epiphysis (ep) in the forebrain region. E. spinal cord reporter localisation, alongside the dorsal root ganglia (DRG) which are labelled by Hu/ELAV. Additional abbreviations: e, eye; g/all, geniculate/anterior lateral line ganglia; mmV, maxillomandibular trigeminal ganglion; opV, ophthalmic trigeminal ganglion; p, petrosal ganglion; pll, posterior lateral line ganglion.

We hypothesised that the ancestral vertebrate CNEs are regulatory elements responsible for expression in territories shared between the genes in jawless and jawed vertebrates. To investigate this, we selected a region with multiple CNEs nearby (named *ERR8t12* and containing CNE6, CNE7 and CNE9; Fig. 5C), which was cloned from lamprey genomic DNA and tested for its ability to drive reporter expression in transgenic lamprey embryos generated by i-SceI meganuclease integration into zygotes (Fig. 6, Supplementary Figure S5). Reporter expression viewed in live embryos overlapped with domains of the endogenous *ERRa* expression, with 36.3% of embryos showing expression in the brain, and 40.3% in more posterior neural tube (Fig. 6A, Supplementary Figure S5). We also saw expression of the reporter in the branchial arches and head muscles in many embryos (Supplementary Figure S5). These sites do not map to sites of *ERRa* expression and are common ectopic sites when using this plasmid vector (Papadogiannis et al., 2022).

Since precise sites of expression in the CNS were difficult to see in live embryos and cranial ganglia difficult to distinguish from underlying CNS, we stained fixed embryos using antibodies against GFP and the neural differentiation marker Hu/ELAV and recorded detailed expression via optical fluorescence microscopy (20 embryos) and confocal microscopy (3 embryos) (Fig. 6C-E). This gave single cell level resolution and revealed GFP in sites that could not be seen in live embryos. It confirmed CNS expression mirrored the expression of *ERRa*, with reporter expression in the olfactory system (43.5% of embryos) and epiphysis (30.5% of embryos)) (Fig. 6C, D), midbrain (69.5% of embryos) (Fig. 6C) and spinal cord (56.5% of embryos) (Fig. 6E). Activity was also seen in the eye (56.5% of embryos), in a pattern similar to the *ERRa in situ* hybridisation signal (Supplementary Figure S3). However, GFP staining in the cranial ganglia (including the VA) (21.5% of embryos) was not detected above the level seen in transgenic vector-only transgenic control embryos (Papadogiannis et al., 2022), and as viewed by confocal microscopy showed signal in only a few cranial ganglia cells in the two embryos screened (Supplementary Figure S6).

**Figure 7:**
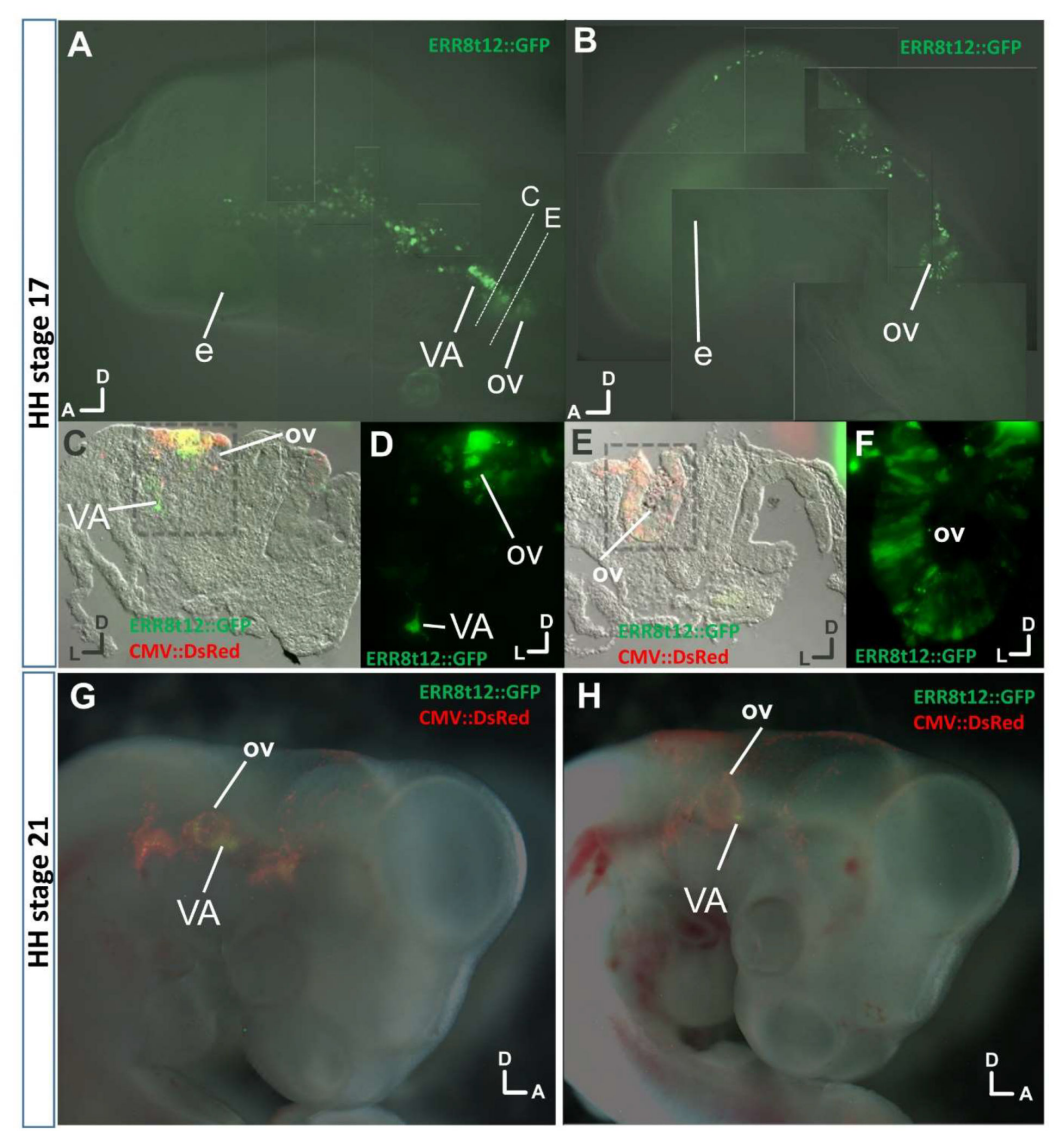
Lamprey *ERR8t12* CNE activity in chicken embryos A. HH17 embryo showing *ERR8t12* activity (green) in the otic vesicle (OV) and vestibuloacoustic ganglion (VA). Lines labelled C and E mark the planes of section shown below. B. HH17 embryo showing *ERR8t12* activity in the otic vesicle. C. Transverse section at the level of the VA of the embryo in A, as shown by the dotted line in A, and overlaying DsRed and GFP. The framed region is shown in D. E. Transverse section at the level of the otic vesicle of the embryo in A, as shown by the dotted line. F. Close-up of the region framed in E, with overlay of multiple sequential focal planes revealing green fluorescence in the otic vesicle. G, H. HH stage 21 embryos showing *ERR8t12* activity (green) in the otic vesicle and VA. Embryos were co-electroporated with a control construct in which DsRed was driven by CMV (CMV::DsRed). Embryo numbers are n=3 (HH17) and n=7 (HH21). Orientations dorsal (D) and anterior (A) or lateral (L) are shown. e, eye.

Since detecting weak reporter signal in lamprey embryos was hampered by both background and the need to fix and stain transgenic embryos, we sought an alternative approach to testing for reporter expression in the VA. Building on the hypothesis that sequence conservation reflects evolutionary constraint on sequence change due to conserved upstream regulation, we considered that *ERR8t12* function should extend to other vertebrates. To explore this, we tested lamprey *ERR8t12* reporter activity in transgenic chicken embryos. Embryos were electroporated at stage HH10 targeting DNA constructs to the head ectoderm, including cells from where the otic vesicle and VA develop. Imaging and sectioning of embryos showed that lamprey *ERR8t12* was able to drive reporter expression specifically in the otic vesicle and VA at both stages, but not more broadly in head ectoderm (Fig. 7).

## Discussion

### *ERR* gene family evolution

The *ER* and *ERR* genes were proposed to have originated from an ancestral NHR gene early in animal evolution (Bertrand et al., 2004). Our data suggest that this occurred by tandem gene duplication, as the genes are linked in many species. Molecular phylogenetic analysis resolved *ER* and *ERR* gene families well, as seen previously (Bertrand et al., 2004; Filowitz et al., 2018). Most invertebrates have a single *ERR* gene, while vertebrates had three or more *ERR* genes. Lamprey and hagfish *ERR* genes were placed with other vertebrate *ERR* genes as expected, but not as clear orthologues of *ERRα, ERRβ, ERRγ* or ERR*δ*. Comparing synteny between *ERR* loci in lamprey and jawed vertebrates showed they evolved by block duplication of a single ancestral locus. This is consistent with the evolution of lamprey and jawed vertebrate genomes by chromosome and genome level duplications (Simakov et al., 2020). It also showed that the lamprey *ERRa* and jawed vertebrate *ERRγ* loci are most similar to each other, as they share proximity with several single copy genes. This, along with similarities in expression and CNE content (discussed below) mean *ERRa* and *ERRγ* are probably orthologues.

### *ERR* gene expression in invertebrates and vertebrates

While *ERR* expression in jawed vertebrates like zebrafish and mice has been quite well described, expression in invertebrate embryos has been poorly documented. We evaluated *ERR* expression in the embryos of three additional invertebrates, plus lampreys. Comparison between these species and jawed vertebrates allows us to infer conserved and derived aspects of *ERR* expression.

#### ERR expression in the nervous system

All species examined showed ERR expression in the nervous system. Lamprey *ERRa* was expressed in several sites in the developing CNS, including the eye, epiphysis, olfactory system, hindbrain and spinal cord. Many of these sites are shared with jawed vertebrate embryos, suggesting they are conserved. *ERR* appears to be confined to the endoderm of *Drosophila* (Thurmond et al., 2019), however we observed *ERR* in the nervous systems of all three invertebrates we studied. The limpet produces a typical planktonic trochophore larva and we observed *ERR* expression in the cells of its apical organ, a ciliated sensory structure sited at the animal pole. In some species, the apical organ mediates multiple stimuli including mechanosensation and chemosensation (Marlow et al., 2014). Different cell morphologies in the limpet apical organ (Fig. 3H, K) suggest that it may also mediate multiple stimuli, although this has not been experimentally validated.

Both species of amphioxus show *ERR* expression in CNS and PNS (this study; (Bardet et al., 2005; Ren et al., 2020)). CNS *ERR* expression includes the frontal eye (part of the anterior CNS and a homolog of the vertebrate eye (Vopalensky et al., 2012)), plus the photoreceptor cells of Hesse, scattered light-sensitive cells found more posteriorly in the CNS (Bardet et al., 2005). This association with the visual system may be conserved across chordates, given *ERR* expression in lamprey and jawed vertebrate eyes and the tunicate ocellus (discussed further below). Amphioxus *ERR* is also expressed in some other cells along the length of the anterior CNS whose precise identity is unknown (Ren et al., 2020). In mice, single cell sequencing has identified *ERRγ* as expressed in neurons in the dI2 region of the spinal cord (which houses commissural neurons) as well as in ventral interneuron populations (Delile et al., 2019). Embryonic lamprey spinal cord neurons have not been sufficiently well described to determine if *ERRa* is expressed by equivalent cells, though the activity of *ERR8t12:GFP* shows there is probably conservation of regulation. It is likely at least some of the *ERR* positive cells in lamprey and jawed vertebrate spinal cords are of the same type, though proving this will require further study.

Amphioxus *ERR* expression is also found in epidermal sensory neurons, part of the PNS. Some amphioxus epidermal sensory neurons delaminate to lie between the epidermis and underlying mesendoderm, leaving their sensory cilium projecting through the epidermis to the outside world (Mazet et al., 2004; Schubert et al., 2004). Their function has not been tested, though it is likely some are mechanosensory and some chemosensory (Parker, 1908; Satoh, 2005). Their developmental history is distinct from vertebrate cranial ganglia neurons as they are born in the ventral embryonic ectoderm as scattered precursors (Lu et al., 2012) rather than in dorsal placodes. However, they do express some genes characteristic of vertebrate placodes such as *Eya* and *Six1/2* (Kozmik et al., 2007). One possibility that accounts for these conflicting data is that the cells are comparable to vertebrate cranial ganglia cells at the level of the cell types concerned, but not at the level of the placode or ganglion morphogenesis (Patthey et al., 2014). *ERR* is restricted to a subset of epidermal neurons in the middle of the animal, alongside the ‘rhombospinal’ part of the amphioxus neural tube which is homologous (based on Hox expression) to the hindbrain and anterior spinal cord region of vertebrates (Albuixech-Crespo et al., 2017; Ferran and Puelles, 2019). If *ERR* expression in these amphioxus cells is conserved with vertebrates it would imply homology to vertebrate VA *ERR*-expressing cells. Our data neither support nor conflict with this possibility.

Tunicates are the sister group to vertebrates. A previous study has described spatial *ERR* expression in tunicate development, revealing expression in parts of the sensory vesicle of the ascidian *P. mammillata* but not defining the precise cells. In the tunicate *Ciona*, we identified *ERR* expression in the CNS associated with cells of two sensory organs, the ocellus and the otolith. Expression in the ocellus is interesting, as the organ has been suggested to be homologous to the vertebrate retina (Kusakabe et al., 2001) and our mining of scRNAseq data also suggests these cells have a visual system function. This adds support to the contention discussed above that *ERR* expression in the visual sensory system of the anterior CNS is an ancestral chordate character still shared by amphioxus, tunicates, lampreys and jawed vertebrates.

The *Ciona* otolith system functions as a gravity sensor (Tsuda et al., 2003), helping the swimming larva swim downwards to settle and metamorphose. The system is thought to work as an inertial sensor, detecting displacement of the otolith pigment cell. Our data suggest *Ciona ERR* expression near the otolith is in cells known as ‘antenna cells’, based on their position and on *ERR* co-expression with *Vglut* in scRNAseq data. Antenna cells are neurons that relay sensation of otolith displacement to the processing part of the brain (Ryan et al., 2017). This has functional equivalence to inertial sensing by the otic/VA system of vertebrates but does not appear to be homologous in the classical sense, as in *Ciona* these cells develop from within the CNS and not from sensory placodes and cranial sensory ganglia. Furthermore, good candidates for placode homologs are present in *Ciona* and produce some sensory neurons (Horie et al., 2018a; Mazet et al., 2005; Papadogiannis et al., 2022), but we did not detect *ERR* at all in the *Ciona* PNS either by in situ hybridisation or through examining published scRNAseq data. We therefore suggest that if these *ERR* expressing systems do have shared ancestry it is also at the level of the cell type. We can speculate that the common sensory theme is mechanosensation: in the VA/otic system of vertebrates, the otic system of *Ciona* and the epidermal sensory cells of amphioxus. This could be tested by establishing the downstream targets of *ERR* genes in the respective cells in these different species.

#### Mesoderm *ERR* expression in amphioxus, *Ciona* and vertebrates

In both *Ciona* and amphioxus ERR genes are expressed by segmented muscle cells (Bardet et al., 2005). In amphioxus these cells derive from multicellular somites, while in *Ciona* these are individual muscle cells arranged segmentally on either side of the notochord. In zebrafish *ERR* expression in somites and other mesoderm derivatives has also been reported (Bardet et al., 2004; Bertrand et al., 2007), which could point to somite expression as conserved across the chordates. However, we did not identify *ERR* expression in these tissues in lamprey. If mesodermal *ERR* expression is ancestral for chordates it has either been lost by lampreys or occurs at a later stage in the life cycle than those analysed here.

#### A summary of the evolution of ERR expression

Neural and possibly mesodermal expression appear to be ancestral for chordate ERR genes. The visual system expresses ERR genes in all the chordate lineages examined. Cell type expression also suggests a connection to mechanosensation, albeit speculative as there are also substantial differences here between amphioxus, *Ciona* and vertebrates. Within vertebrates, expression of *ERRa* in lamprey and *ERRγ* in other vertebrates show similarity in the eye, other parts of the CNS and VA. This suggests the presence of a conserved regulatory network in the vertebrate common ancestor, driving *ERR* expression in these tissues, inherited by *ERRa*/*ERRγ*. Mapping and testing of potential regulatory elements allowed us to examine this further.

### The evolution of vertebrate *ERR* regulation

Lamprey *ERRa* and jawed vertebrate *ERRγ* share CNEs with the same order, orientation and intron location. We did not find similar CNEs around other *ERR* paralogues. These features indicate *ERRa* and *ERRγ* are probably orthologues, and we can definitively conclude that the vertebrate common ancestor had at least one *ERR* gene with this expression pattern and CNE suite. A follow-on prediction is that similar *ERR* expression in lamprey and jawed vertebrates is controlled by these CNEs. Partly confirming this, our sequence analysis showed that one CNE (*EL161*) is shared with lampreys and has been previously shown to drive reporter expression into the zebrafish CNS (Roberts et al., 2014). We focused experimental analysis on the *ERR8t12* CNE cluster in the second-to-last intron of *ERRa* since this includes three of the remaining four lamprey CNEs. *ERR8t12* activity in transgenic lamprey embryos showed overlap with *ERRa* expression domains in the CNS, including the eye, olfactory system, epiphysis, and along the spinal cord. This confirms *ERR8t12* can function as an enhancer, driving reporter expression into sites in the CNS that express *ERRa*.

While low level *ERR8t12* reporter expression was observed in the cranial ganglia of transgenic lampreys, the weak activity was similar to that previously observed in vector-only control transgenics (Papadogiannis et al., 2022). However, *ERR8t12* activity in the otic and VA system of transgenic chicken embryos provides some evidence for a conserved role in these tissues. These results make the lack of clear *ERR8t12* activity in the VA of transgenic lampreys surprising, as we might expect element activity to be more robust in a same-species environment than when tested cross species. One possibility is *ERR8t12* drives lamprey VA activity at different developmental stages than the ones analysed here. This is possible as detecting some aspects of transgene expression required fixation and antibody labelling, limiting the stages we could census.

Combined, these data show that the expression of this subset of vertebrate *ERR* genes was inherited from the expression of one ancestral *ERR* gene and that this was regulated by conserved regulatory elements in the 3’ introns. This shows the gene regulatory network driving *ERR* expression in these conserved sites evolved before the separation of lamprey and jawed vertebrate lineages. This network presumably came under sufficient constraint to be maintained from that point onwards. Similarly, the absence of conservation outside of vertebrates suggests it is vertebrate specific. This last point will be difficult to test directly, though it might be insightful to determine how *ERR* is regulated in one or more invertebrate outgroups, as we would not expect it to be conserved with vertebrates.

## Supplementary Material

Supplementary figures
