# Supplementary material for: Evolution of the expression and regulation of the nuclear hormone receptor ERR gene family in the chordate lineage
Source: Dev Biol. Author manuscript; Available in PMC 2025 Jul 4. (PMC7617844; doi:10.1016/j.ydbio.2023.09.003)
Supplement: Supplementary figures [file EMS206502-supplement-Supplementary_figures.pdf]

Supplementary information: Evolution of the expression and regulation of the nuclear hormone receptor *ERR* gene family in the chordate lineage

Vasileios Papadogiannis, Dorit Hockman, Silvia Mercurio, Claire Ramsay, Mark Hintze, Cedric Patthey, Andrea Streit, Sebastian M. Shimeld

Contents

Supplementary Figures and Tables

Supplementary Figure S1. *Ciona* and amphioxus scRNAseq data analysis
Supplementary Figure S2. Lamprey *ERRc* embryo expression.
Supplementary Figure S3. *ERRa* expression and *ERR8t12* CNE activity in the lamprey eye
Supplementary Figure S4. Synteny comparison of jawed vertebrate *ERRγ* loci
Supplementary Figure S5. Reporter expression in representative lamprey embryos
Supplementary Figure S6. Cranial sensory ganglia reporter expression

Supplementary Table S1. Evaluation of ERR transcripts in the eye RNAseq data
Supplementary Table S2. Primer sequences used in this study.

Supplementary appendix

1. ERR exon alignments.
For these ‘n’ refers to the last exon, n-1 the second to last and so on

- ERR-γ exon n-2 alignment in nucleotide and amino acid versions
- ERR-γ exon n-1 alignment in nucleotide and amino acid versions
- ERR-γ exon n alignment in nucleotide and amino acid versions

2. ERRγ and ERRa protein sequence alignment
This shows the alignment of jawed vertebrates ERRγ and lamprey ERRa amino acid sequences.

3. Alignment of CNE sequences as portrayed in Figure 4B, C.
Alignments of CNE sequences from a range of jawed vertebrates are shown. Includes CNEs 1-10 plus EL161.

4. ERR CNE sequences in FastA format
All the CNE sequences used in the alignment in the section above are listed in FastA format

5. NCBI IDs for sequences used in molecular phylogenetic analyses
This table contains the NCBI accession numbers for all the sequenced used in molecular phylogenetic analysis in this study

Supplementary Figure S1. ***Ciona*** **and amphioxus scRNAseq data analysis**
A. *ERR* (KH2012:KH.L8.21) expression in neural cells compared to that of the pan-neural gene *ETR* (KH2012: KH.C6.128) and *VGlut* (KH2012:KH.C3.324), extracted from the *Ciona* scRNAseq data set (Cao et al., 2019). Note expression of *ERR* in the cell clusters named Lox5+ aSV and Rx+ aSV but not in peripheral neuron populations including aATENs, pATENs, RTENs, BTNs and PSCs (palp sensory cells). Note *VGlut* expression in Lox5+ aSV cluster cells but not Rx+ aSV cluster cells.
B. Venn diagram showing genes annotated as related to photoreception in Rx+ aSV cells (25 genes) and Lox5+ aSV cells (19 genes), of which 8 (including *ERR*) are shared. Only selected genes in each compartment are specifically identified.
C. Heat map of amphioxus scRNAseq data (Ma et al., 2022), extracted from the Shiny App built by the authors (available at https://lifeomics.shinyapps.io/shinyappmulti/) on 19 July 2023. *ERR* is shown compared to a selection of marker genes. *ERR* shows expression in mesoderm and neural ectoderm, and weakly in primordial germ cells. Data are not resolved sufficiently to reveal cell types such as PNS within these categories, though *ERR* distribution is consistent with our *in situ* hybridisation data and published amphioxus *ERR* expression data (Bardet et al., 2005; Ren et al., 2020).

Bardet, P.L., Schubert, M., Horard, B., Holland, L.Z., Laudet, V., Holland, N.D., Vanacker, J.M., 2005. Expression of estrogen-receptor related receptors in amphioxus and zebrafish: implications for the evolution of posterior brain segmentation at the invertebrate-to-vertebrate transition. Evol Dev 7, 223-233.
Cao, C., Lemaire, L.A., Wang, W., Yoon, P.H., Choi, Y.A., Parsons, L.R., Matese, J.C., Wang, W., Levine, M., Chen, K., 2019. Comprehensive single-cell transcriptome lineages of a proto-vertebrate. Nature 571, 349-354.
Ma, P., Liu, X., Xu, Z., Liu, H., Ding, X., Huang, Z., Shi, C., Liang, L., Xu, L., Li, X., Li, G., He, Y., Ding, Z., Chai, C., Wang, H., Qiu, J., Zhu, J., Wang, X., Ding, P., Zhou, S., Yuan, Y., Wu, W., Wan, C., Yan, Y., Zhou, Y., Zhou, Q.J., Wang, G.D., Zhang, Q., Xu, X., Li, G., Zhang, S., Mao, B., Chen, D., 2022. Joint profiling of gene expression and chromatin accessibility during amphioxus development at single-cell resolution. Cell Rep 39, 110979.
Ren, Q., Zhong, Y., Huang, X., Leung, B., Xing, C., Wang, H., Hu, G., Wang, Y., Shimeld, S.M., Li, G., 2020. Step-wise evolution of neural patterning by Hedgehog signalling in chordates. Nat Ecol Evol 4, 1247-1255.

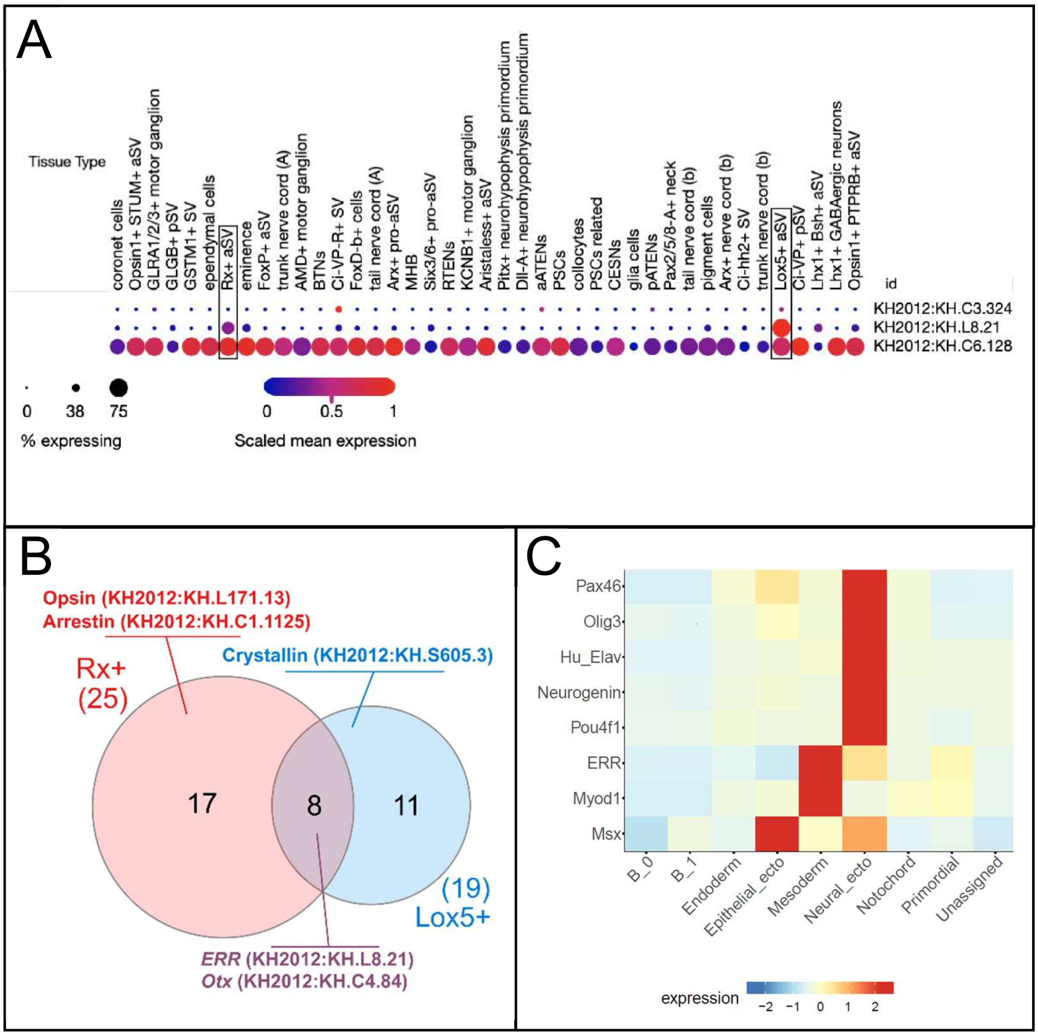
A
Tissue Type
coronet cells
Opsin1+ STUM+ aSV
GLRA1/2/3+ motor ganglion
GLGB+ pSV
GSTM1+ SV
ependymal cells
Rx+ aSV
eminence
FoxP+ aSV
trunk nerve cord (A)
AMD+ motor ganglion
BTNs
Ci-VP-R+ SV
FoxD-b+ cells
tail nerve cord (A)
Arx+ pro-aSV
MHB
Six3/6+ pro-aSV
RTENs
KCNB1+ motor ganglion
Aristaless+ aSV
Pitx+ neurohypophysis primordium
Dll-A+ neurohypophysis primordium
aATENs
PSCs
collocytes
PSCs related
CESNs
glia cells
pATENs
Pax2/5/8-A+ neck
tail nerve cord (b)
pigment cells
Arx+ nerve cord (b)
Ci-hh2+ SV
trunk nerve cord (b)
Lox5+ aSV
Ci-VP+ pSV
Lhx1+ Bsh+ aSV
Lhx1+ GABAergic neurons
Opsin1+ PTPRB+ aSV
id
KH2012:KH.C3.324
KH2012:KH.L8.21
KH2012:KH.C6.128
0
38
75
% expressing
0
0.5
1
Scaled mean expression
B
Opsin (KH2012:KH.L171.13)
Arrestin (KH2012:KH.C1.1125)
Rx+
(25)
Crystallin (KH2012:KH.S605.3)
17
8
11
(19)
Lox5+
ERR (KH2012:KH.L8.21)
Otx (KH2012:KH.C4.84)
C
Pax46
Olig3
Hu_Elav
Neurogenin
Pou4f1
ERR
Myod1
Msx
B_0
B_1
Endoderm
Epithelial_ecto
Mesoderm
Neural_ecto
Notochord
Primordial
Unassigned
expression
−2
−1
0
1
2

Supplementary Figure S2. Lamprey *ERRc* embryo expression.
Expression of lamprey *ERRc* was identified in a small patch of cells in the hindbrain (hb) and in the mandibular arch (ma) at stages 25 and 26.

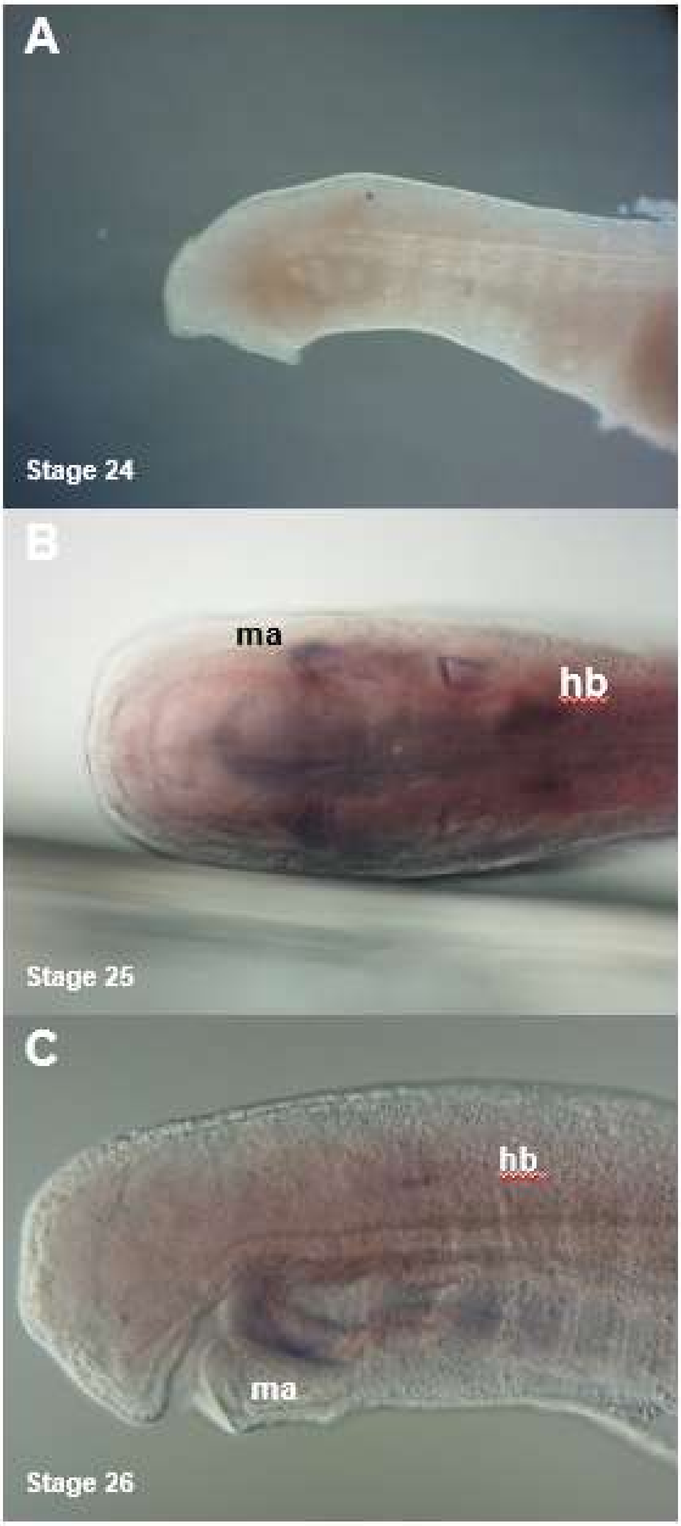

Supplementary Figure S3. ***ERRa* expression and *ERR8t12* CNE activity in the lamprey eye**

A-C. *ERRa* expression in *L. planeri* embryos, focusing on expression in the eye (white arrows). The right (A) and left (B) eyes of a stage 28 embryo in dorsal view are shown in close-up in A' and B' respectively. Two focal planes of the left eye of a stage 29 embryo (C) are shown in C' and C''. D-F. *ERR8t12* activity in stage 29 *P. marinus* embryos, with and close up views of the eyes in D'-F'. For full details of reporter gene expression analysis see main text, Figure 5 and Supplementary Figure 4.

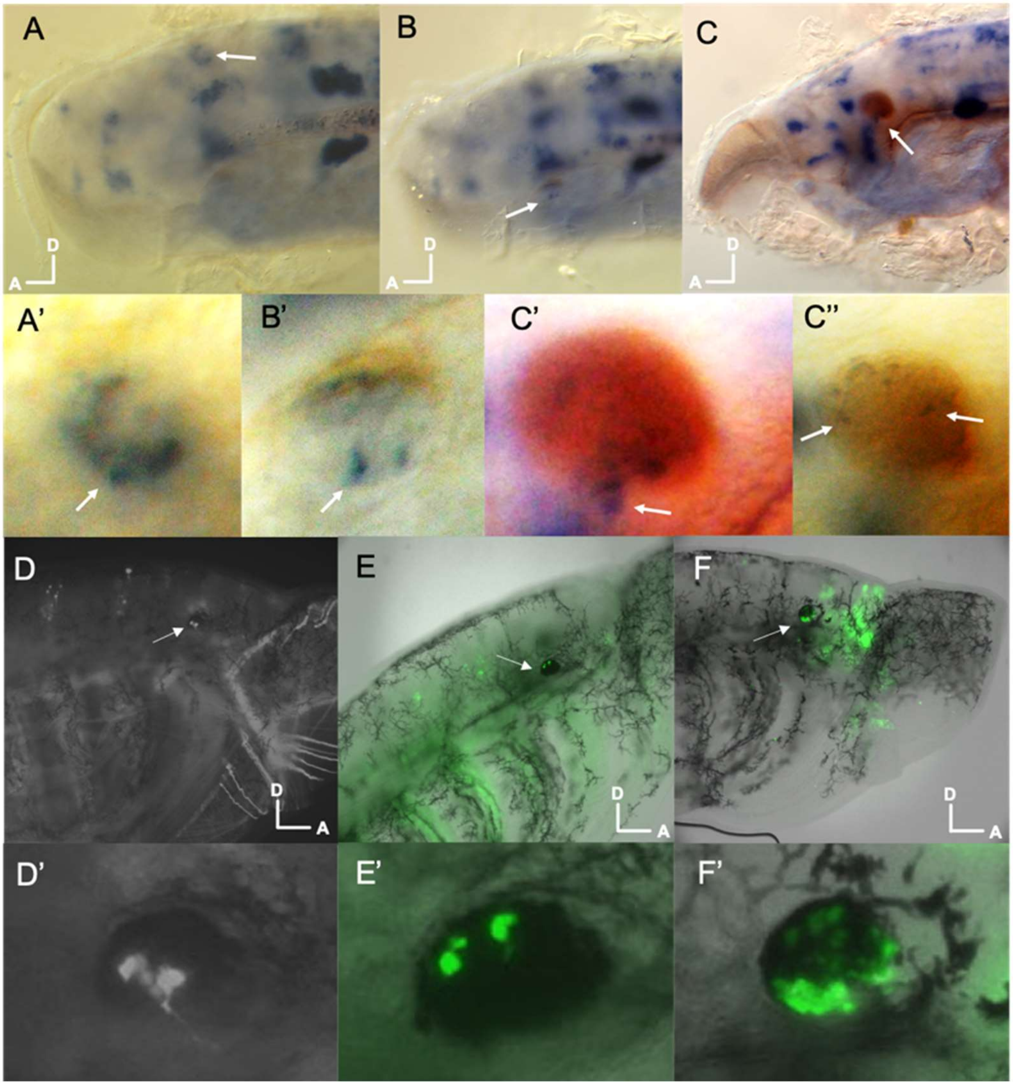

Supplementary Figure S4
Synteny comparison of jawed vertebrate *ERRγ* loci with lamprey *ERRa*. Genes are colour coded by family.

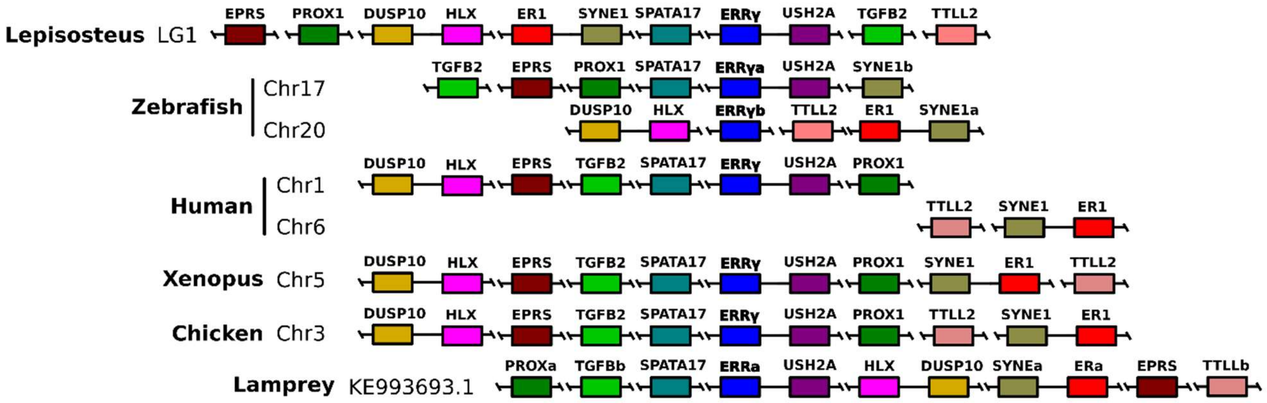

Supplementary Figure S5
Reporter expression in representative lamprey embryos at T26 (A-A'') and T28 (B-D'') showing expression in the sites scored in E.

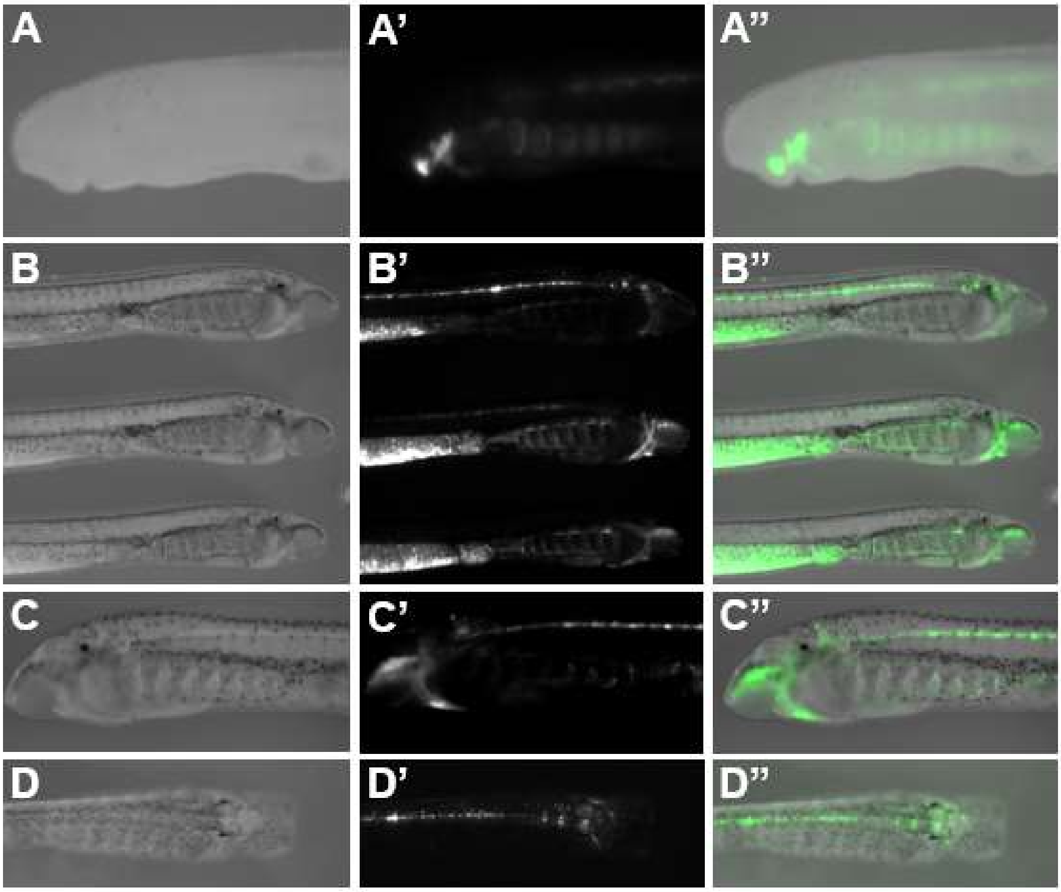

E. Scoring of GFP in live transgenic lamprey embryos. Expression in head mesoderm and musculature (for example as in A and C above) were not scored as these are ectopic sites that do not overlap with endogenous gene expression. Ganglia and finer detail in the brain were difficult to observe in live embryos and are explored by confocal imaging in Figure 5. 295 embryos were generated in total, data are show by embryo and by expression site

Expression by embryo

Location of expression	Number of embryos
Brain, posterior neural tube, branchial arches	80
Posterior neural tube, branchial arches	67
Posterior neural tube, brain	27
Posterior neural tube only	25
Branchial arches only	29
No expression	67
Total scored	295

Expression by site

Expression site	Number of embryos	Percentage of total (295)
Branchial arches	176	59.7%
Posterior neural tube	119	40.3%
Brain	107	36.3%

Supplementary Figure S6
Cranial sensory ganglia reporter expression in *ERR8t12::GFP* transgenic lamprey embryos.

Confocal images of the two embryos showing ERR8t12::GFP reporter expression in cranial sensory ganglia. Hu/ELAV labels neurons (red) and DAPI labels DNA (blue). The Anterior (A) and Dorsal (D) orientation of each image is marked. Labelled structues are the eye (e), the maxiliomandibular ganglion (mmV), vestibuloacistic ganglion (VA) geniculate/anterior lateral line ganglia (g/all) and petrosal/posterior lateral line ganglia (p). White arrows point to ganglia cells expressing both GFP and labelled with Hu/ELAV, which show as yellow.

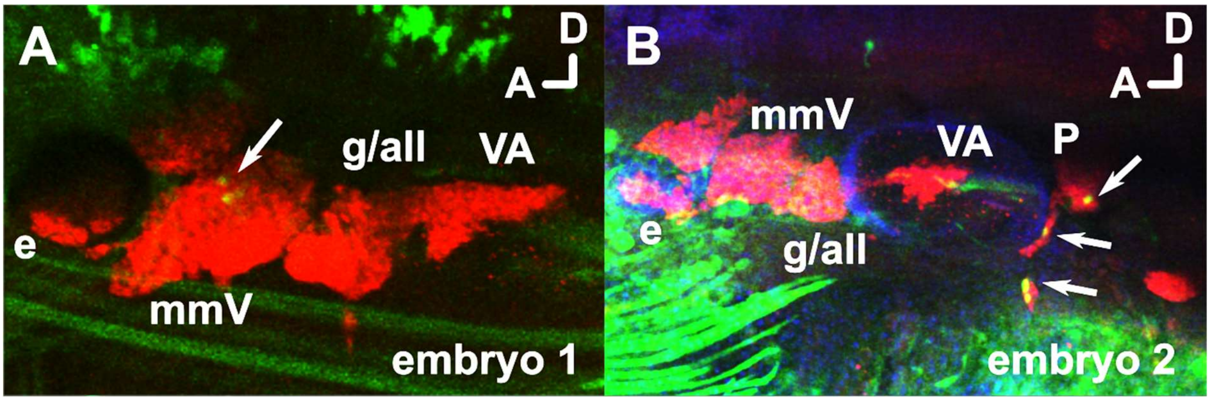

Supplementary Table S1. Evaluation of ERR transcripts in the eye RNAseq data of Lamb et al (2016)

In brief, RNAseq data were assembled then reads mapped back to predicted transcripts. Where more than one transcript matched an ERR gene, we have included them all of separately. These data cannot be compared statistically between species or genes as no biological replicates were available.

Species	Gene	Transcript target_id	Transcript length	Transcript effective length	Read counts	tpm
MORDACIA	ERRA	c42655_g3_i1	1460	1280.67	339	10.44
	ERRC	c51508_g1_i1	424	245.903	38	6.09
	ERRC	c72514_g1_i1	271	103.532	24	9.14
	ERRB	c54569_g1_i1	318	145.131	10	2.72
	ERRB	c55662_g1_i1	202	49.949	3	2.37
EPTATRETUS	ERRA	c156261_g1_i1	4271	4115.56	6364.24	10.88
	ERRC	c156261_g2_i1	3382	3226.56	5765	12.58
	ERRB	c165867_g1_i6	9958	9802.56	2540.04	1.82
	ERRB	c165867_g1_i7	9288	9132.56	5812.89	4.48
	ERRD_fragment1	c352755_g1_i1	839	683.564	31	0.32
	ERRD_fragment2	c335911_g1_i1	225	77.7115	3	0.27
	ERRD_fragment3	c174461_g1_i1	276	123.383	4	0.23
GEOTRIA	ERRA	c60443_g1_i1	1445	1286.15	1191.3	11.43
	ERRC	c60443_g1_i2	1410	1251.15	338.698	3.34
	ERRB	c141828_g1_i1	201	55.8882	8	1.77
	ERRB	c82006_g1_i1	334	176.093	22	1.54
	Hybrid ERRD	c152437_g1_i1	328	170.198	12	0.87

Supplementary Table S2

Primer sequences used in this study. All sequences 5'-3'.

Primer	Sequence
ERRa 5'	CGAGTACATGCTCAACGCCA
ERRa 3'	GTCCTGAAGCTTTTGCACCG
ERRb 5'	CCTCGTCCATGACGAAGTCC
ERRb 3'	GTGCAGACCAAGTGCGAGTA
ERRc 5'	GCAAGTGCGAGTACATGCTG
ERRc 3'	AGGAAAAGTTTGTGCATGGGC
ERR8t12 5'	GCCCACACACCGCCTAATA
ERR8t12 3'	GCATCCGAGAGGAATGCTCA
B. lanceolatum ERR 5'	ATGAGCTGCAGACTTCAGTGG
B. lanceolatum ERR 3'	TCTGGAGCAGGCTCATTTGG
P. vulgata ERR 5'	TCGCGATGGGTTAGGAATGA
P. vulgata ERR 3'	CAACGTCATGGGGGAGACAT

Supplementary appendix

1. Vertebrate ERR exon alignments

In the following figures we show alignments of the last three exons of *ERR* genes from different vertebrate species, accompanied by alignments of the encoded amino acid sequences. Exons are numbered with the last exon=n, and exons before that successively n-1 and n-2, as shown in Figure 5. This is because conservation of intron exon structure means these last exons are clearly homologous. The splice acceptor site is shown in black. Blue shading indicates the degree of conservation as follows:

Darkest blue	all or all but one conserved
Intermediate blue	>50% conserved
Lightest blue	50% conserved
White	<50% conserved

ERRγ exon n-2 (n=last exon) from different jawed vertebrates and *ERRa* DNA alignment above. Translated amino acid sequence alignment below corresponding to DNA alignment. The encoded amino acid sequence corresponds to the first part of the ligand binding domain (see full protein alignment below).

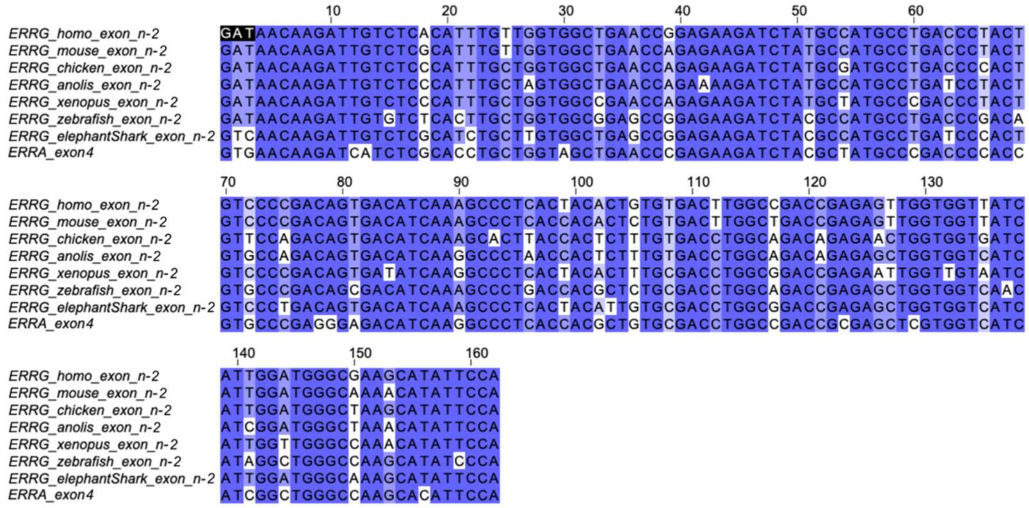

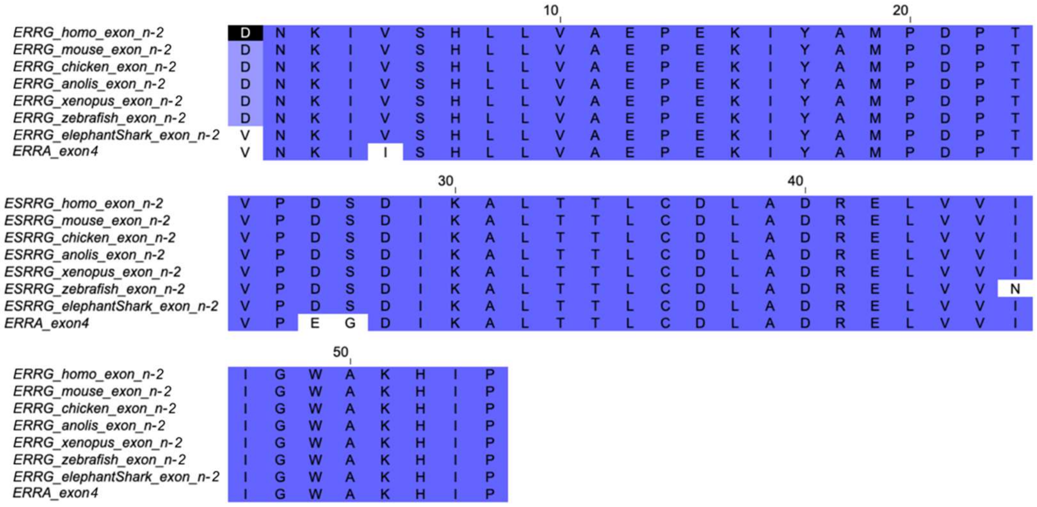

ERRγ exon n-1 (n=last exon) from different jawed vertebrates and *ERRa* DNA alignment above. Translated amino acid sequence alignment below corresponding to DNA alignment. The encoded amino acid sequence corresponds to the second part of the ligand binding domain (see full protein alignment below).

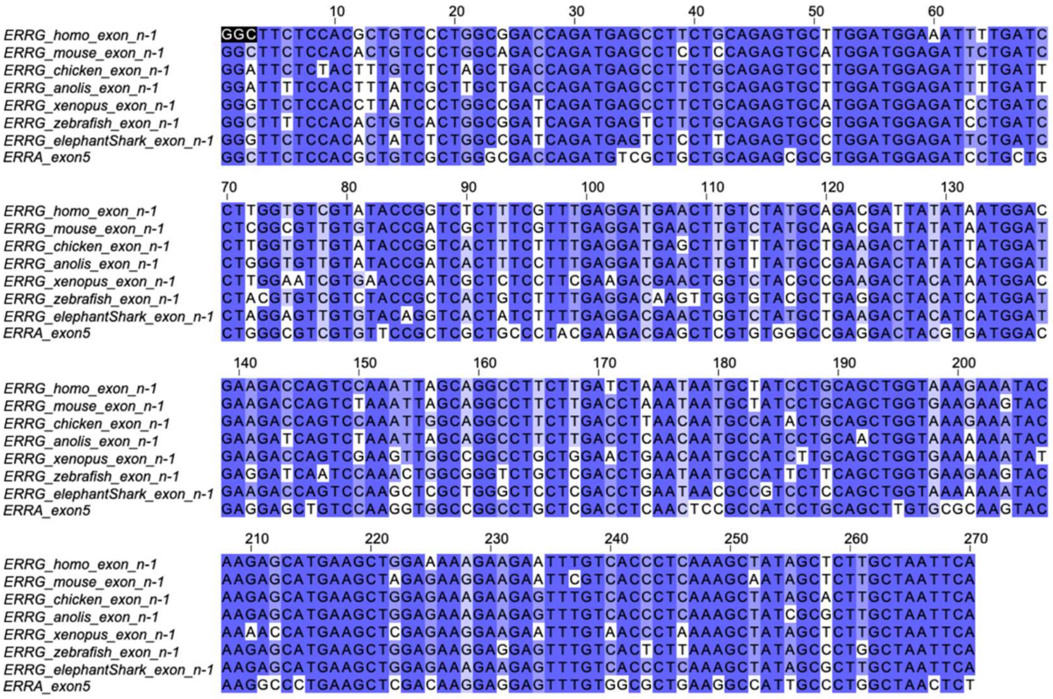

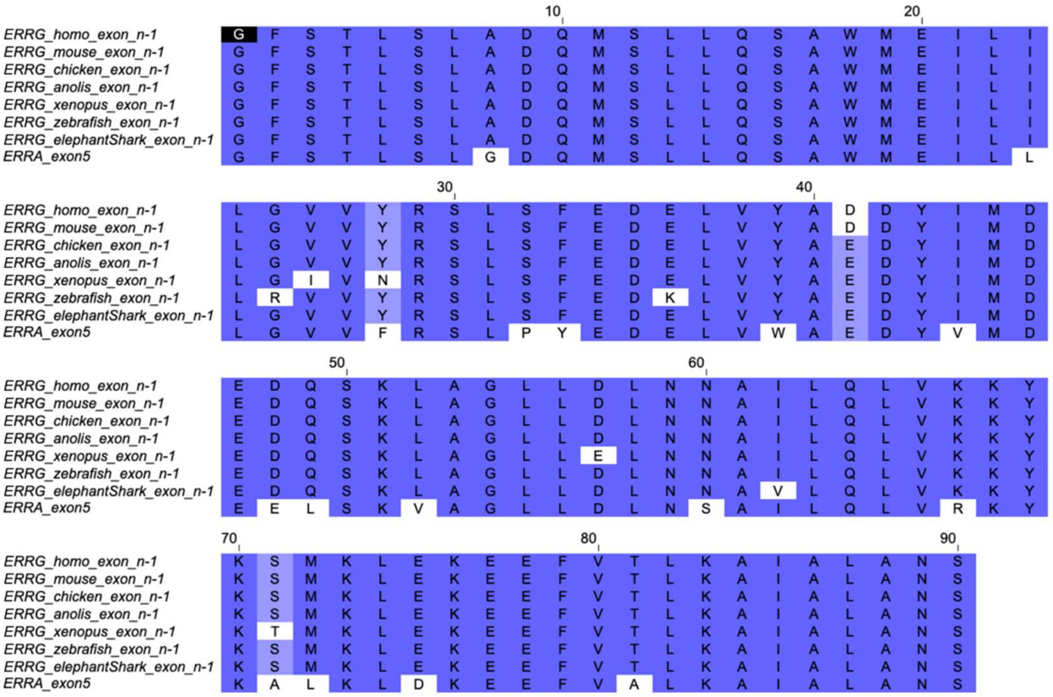

ERRγ exon n (n=last exon) from different jawed vertebrates and *ERRa* DNA alignment above. Translated amino acid sequence alignment below corresponding to DNA alignment. The encoded amino acid sequence corresponds to the third part of the ligand binding domain (see full protein alignment below).

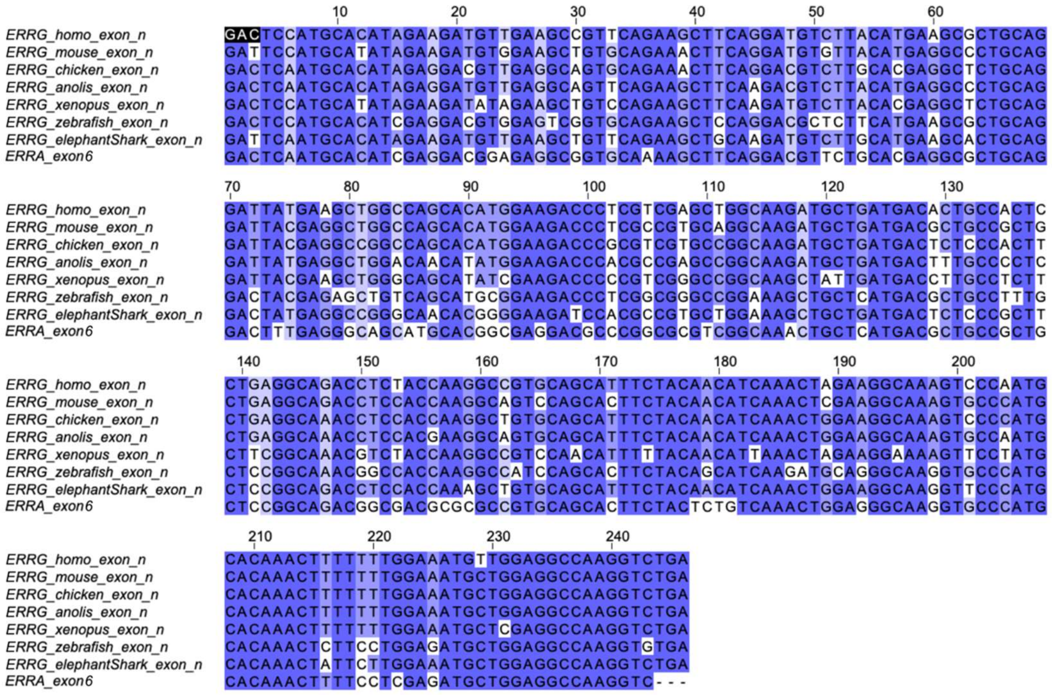

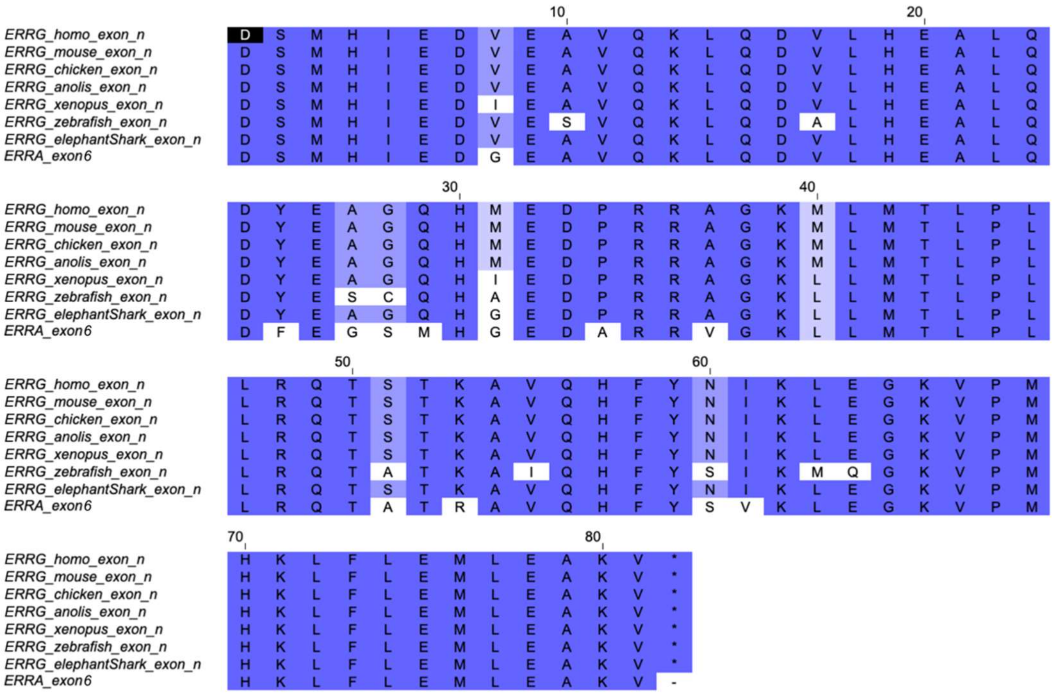

2. ERRγ and ERRa protein sequence alignment. The ligand binding domain is shaded grey and red lines in this region indicate exon boundaries, which split the ligand binding domain is split in three parts encoded by the last three exons (as shown individually above). Underneath the alignment is a consensus sequence with the black bars indicating the extent of conservation supporting this.

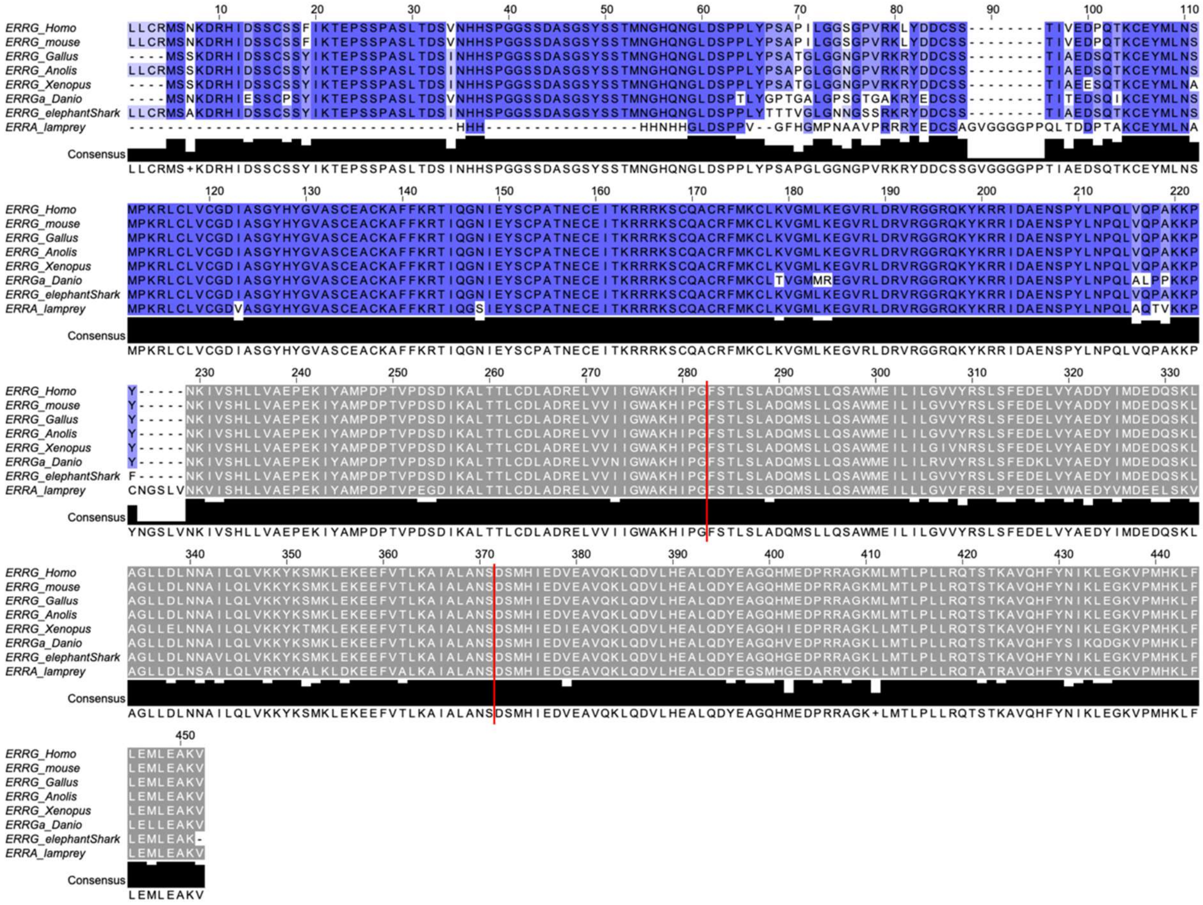

3**. CNE sequences as portrayed in Figure 4B, C**. Alignments are shown first. These were produced by TCOFFEE (https://tcoffee.crg.eu/apps/tcoffee/do:regular), with shading by Boxshade (https://junli.netlify.app/apps/boxshade/). In some cases only the conserved core of the alignment is shown, with numbering indicating where this is in the overall alignment Full sequences follow the alignments, in FASTA format.

CNE1

```
                241 .......250.......260.......270.......280.......290.......300
CNE1_Anolis     236 CCTACAGAAGAAATTTAGTTGTTCAGAGTTTGCATCATAAATGGCTTATAAACGAGCTTT
CNE1_Zebrafish   25 ----------------------------------------------------------TT
CNE1_Xenopus     35 ------------------------------------------------------------
CNE1_Chicken    169 CACACTGTTGAGATTTAGTCATTC----TCTGC--TATAAATGGTGTATAAACAAGCTTT
CNE1_Mouse      103 --TTTCCTTTAGCTTTAGTT----------T-----TCAAATAGTTTATAAAGAAGTTTC
CNE1_Human       77 --TTTGTTTGGAGTTGAATT----------T-----TCAGATAGTTTATAAACAAGTTTT

                301 .......310.......320.......330.......340.......350.......360
CNE1_Anolis     296 TGAATCATGAGCAATGTTTTTCTGGTA----CAGTTAACTTATTTCTG-TCTCCTTAAAC
CNE1_Zebrafish   27 -----------------------------------TGACTCTTTTCT----------GAC
CNE1_Xenopus     35 -----------CAATGTGTTTCAGCTG----TGGTTAACTTCTGTCTTTTTTCCCTAAAC
CNE1_Chicken    223 TGTATCACAAGCAGTGTTTTTCTGGTA----CAGTTAACTTATTTCTG-TCTCCTTAAAC
CNE1_Mouse      146 CAAGTGACAAGAGATGTTTTTCTGGCACACACAGTTAGCTTATTTCTG-TCTCCTTAAAC
CNE1_Human      120 TGAATGATGAGCAATGTTTTTCTGGTA----CAGTTAACTTATTTCTG-TCTCCTTAAAC

                361 .......370.......380.......390.......400.......410.......420
CNE1_Anolis     351 TAGTTTGTCTTTATGCTTTGAATGCTACAGTAATTT-CTTTAATCAAATTGATGGAAGTC
CNE1_Zebrafish   42 TAGTTTGTCTCTGCGGTTTCTATGGTACAGTAATTTTTTTTAATCAAATTGATGAAGGCC
CNE1_Xenopus     80 TACTTTGTCTTTATGCTTTGAATGCTACAATAATTT-CTTTAATCAAATTGATGGAGGTC
CNE1_Chicken    278 TAGTTTGTCTTTATGCTTTGAATGCTACAGTAATTT-CTTTAATCAAATTGATGGAAGTC
CNE1_Mouse      205 TAGTTTGTCTTTATGCTTTGAATGCAACAGTAATTT-CTTTAATCAAATTGATGGAAGTC
CNE1_Human      175 TAGTTTGTCTTTATGCTTTGAATGCTACAGTAATTT-CTTTAATCAAATTGATGGAAGTC

                421 .......430.......440.......450.......460.......470.......480
CNE1_Anolis     410 CCTTTCCCAGCCCAC-ACTGAAACCAATTTATTGGTCATGATACGCCAGCTATGTTACTA
CNE1_Zebrafish  102 TCCATTCTCCCCGAGAGCCCAAACCAATTTATGGCTCATGATACGGCGGCTATGTTGCTA
CNE1_Xenopus    139 CCTTTCCCAGCCCCA-ACTGAAACCAATTTATTGGTCATGATACGCCGGCTATGTTGCTA
CNE1_Chicken    337 CCTTTCCCAGCCCAC-AGTGAAACCAATTTATTGGTCATGATACGCCAGCTATGTTGCTA
CNE1_Mouse      264 CCTTTCCCAGCCCAC-ACTGAAACCAATTTATTGGTCATGATACGCCAGCCATGTCGCTA
CNE1_Human      234 CCTTTCCCAGCCCAC-ACTGAAACCAATTTATTGGTCATGATACGCCAGCTATGTTGCTA

                481 .......490.......500.......510.......520.......530.......540
CNE1_Anolis     469 ACAGTCGTAACGAACTGTCAGGCAATATCGCAGTGAAGCTAATTTGAAGTCATTAGGTGT
CNE1_Zebrafish  162 ACAGACGTAACAAACTGTCAGCC--TCTCGCACAGAAGCTAATTTGAAGTCATTAGGTGT
CNE1_Xenopus    198 AGAGTCGTAACGAACTGTCAGGCAATATCGCAGTGGAGCTAATTTGAAGTCATTAGGTGT
CNE1_Chicken    396 ACAGTCGTAACGAACTGTCAGGCAATATCGCAGTGAAGCTAATTTGAAGTCATTAGGTGT
CNE1_Mouse      323 ACAGTCGTAACGAACTGTCAGGCGGTATCGCAGTGAAGCTAATTTGAAGTCATTAGGTGT
CNE1_Human      293 ACAGTCGTAACGAACTGTCAGGCGATATCGCAGTGAAGCTAATTTGAAGTCATTAGGTGT

                541 .......550.......560.......570.......580.......590.......600
CNE1_Anolis     529 GCAGCGTTCAGGCAACAATTTGTTACAGTGGTTAGGAACACAGCTGACATTTCTGAATGG
CNE1_Zebrafish  220 GCAGCGTACGGGCAACAATTTGTTACGGTGGTTAGGAGCGCCGCTGACATTTCTGAGCGC
CNE1_Xenopus    258 GCAGCGTTCAGACAACAATTTGTTACAGTGGTTAGGAGCGCAGCTGACATTTCTGAATGG
CNE1_Chicken    456 GCAGCGTTCAGGCAACAATTTGTTACAGTGGTTAGGAACACAGCTGACATTTCTGAATGG
CNE1_Mouse      383 GCAGCGTTCAGGCAACAATTTGTTACAGTGGTTAGGGACACAGCTGACATTTCTGAATAG
CNE1_Human      353 GCAGCGTTCAGGCGACAATTTGTTACAGTGGTTAGGAACACAGCTGACATTTCTGAATGG

                601 .......610.......620.......630.......640.......650.......660
CNE1_Anolis     589 CTTTGTTCA-GAGC-AGGTCTAGATGAGC-CCAAGCAAATTGTAAACTAATTTAAACATC
CNE1_Zebrafish  280 CTTCGCTTACGATC-TGTCCTAGGCGAGGAGCAGGCAAATTGTAAACTAATTTAAACATC
CNE1_Xenopus    318 CTTTGTTCA-GAGCTAGAACTATATGAGC-CCAAGCAAATTGTAAACTAATTTAAACATC
CNE1_Chicken    516 CTTTGTTCA-GAGC-AGTTCTAGATGAGC-CCAAGCAAATTGTAAACTAATTTAAACATC
CNE1_Mouse      443 CTTTGTTCA-GGGC-AGTTCTAGATGAGA-CCAAGCAAATTGTAAACTAATTTAAACATC
CNE1_Human      413 CTTTGTTCG-TTGT-AGTTCTAGATGAGG-CCAAGCAAATTGTAAACTAATTTAAACATC

                661 .......670.......680.......690.......700.......710.......720
CNE1_Anolis     646 ACTCTGTATTATACTA-----GCTACTTTGTCTTACA------CTGGTTGCTAATAAAGC
CNE1_Zebrafish  339 ACTCAGTATTAC--------------------------------TG----------GAGC
```

```
CNE1_Xenopus     376 ACTCTGTATTATACCACCCCGGCTACTTTTTTTCCACCGACGCCGCTTTCTAATAAAGC
CNE1_Chicken     573 ACTCTATATTATACTA-----GCTACTTTGTCTTACA------CTGGTTGCTAACAAAGC
CNE1_Mouse       500 GCTCTGTATTATACCA-----GCTACTTTGTCTTACA------CTGTGTGCTAATAAAGC
CNE1_Human       470 ACTCTGTATTATACCA-----GCTACTTTGTCTTACA------CTGTTTGCTAATAAAGC

                 721 .......730.......740.......750.......760.......770.......780
CNE1_Anolis      695 ACCCCAGATTAAGTCAAAGCTAGTTAAATAGGTGAGTTTGAGTTTCATCTCTAGACAATG
CNE1_Zebrafish   357 --------------------TG--------------------------------------
CNE1_Xenopus     436 GCCCCGAATTAAGTCAAAGCTGGTTAAATAGGTGAGTG------TATTATTTT-------
CNE1_Chicken     622 ACCCCAAATTAAGTCAAAATGAGTTAAACAGGTGAGTTTGAGCTTCATCTTTGGAACATA
CNE1_Mouse       549 TCTCCAAATTAAGTCAGAACTGGTTAAATAGGTAAC-TCGAATGTATGCTTTGGCAAGCA
CNE1_Human       519 CCCCCAAATTAAGTCAAAACTAGTTAAATAGGTGAGCTTGTA--TATTCTTTAGAAGACA
```

CNE2

```
CNE2_Anolis          146 AAGGGGTTAT------CAGCATATAGAATTAG-CTT--------ACTTGCATT--AA---
CNE2_ElephantShark    85 ATTACTTTAT------CAGTAAATAGAATTAG-GCT--------TGCCTCGCT--AA---
CNE2_Zebrafish        20 -----TTTAG------CATG--ACAGGA-----------------CCCTCACT--AT---
CNE2_Xenopus          79 TGGCCTTTAT------CAGCAAATAGAATTAG-CCT--------AGTAAGATT--TA---
CNE2_Chicken         136 AGGCCTTTAT------CAGTAAATAGAATTAG-TCG--------CCTTGCATT--TA---
CNE2_Mouse           149 --CACACTATCTACAGAAGGAAAAAGAAAGAAAGAAAGAAAGAAAGAAAGAAAGAAAGAA
CNE2_Human            65 TTTTTTTTAT------CAGTAAATAGAATTAG-TTG--------ACTTACATT--AA---

                     241 .......250.......260.......270.......280.......290.......300
CNE2_Anolis          186 -TAAAT-------CAAACAAATGA-AAAGTA--TT-------------------------
CNE2_ElephantShark   125 -TAAAG-------CGGCTGAGCAA-AAAGCAG-GC-------------------------
CNE2_Zebrafish        45 -TA---------------------------------------------------------
CNE2_Xenopus         119 -TAAAT-------CAAATAAATGG-AAGGTATTTT-------------------------
CNE2_Chicken         176 -TAAAT-------CAAATAAATGG-AAAGTGTGTT-------------------------
CNE2_Mouse           207 AGAAAGAAAGAAAGAAAGAAA-GG-AAGGAAGGAAGGAAGGAAGGAAGGAAGGAAGAAAG
CNE2_Human           105 -TAAAT-------CATATAAATGGGAAAGTGTGTT-------------------------

                     301 .......310.......320.......330.......340.......350.......360
CNE2_Anolis          210 --------------GTT----GTGATACA-TGACCACATCTTATCTG--GCG-ATGA--A
CNE2_ElephantShark   150 --------------GTT----ATGAAATT-TGAACAGTCCTTATCTG--AC---------
CNE2_Zebrafish        47 ---------------------------CA-GGCC----CTTTATT---------------
CNE2_Xenopus         145 --------------GTT----GTGAGGCA-TGATGGCGGCTTATCTA--GGGGATGG-GG
CNE2_Chicken         202 --------------GTT----ATGAAACA-TGACCATGTCTTATCTG--AAG-AAGA--A
CNE2_Mouse           265 AAAGAAAGAAAGAAAGAAAGAAAGAAAGAGAGAGAAAGAGAGAGAAAGAGAG-A-GA-AA
CNE2_Human           132 --------------GTT----ATGAAACA-TGACCATACTTTATCTC--TAG-AAGAAAA

                     361 .......370.......380.......390.......400.......410.......420
CNE2_Anolis          246 AATGATCAGAGTGGCATTAGG-AAA-TTCTTGACTTTAGTT----------ACAGGAGCG
CNE2_ElephantShark   180 AAAGAGCACAATGGAATTAGA-CAG-CACTTGACTTCAGTCATTTCAAGTTAGAGCAGAA
CNE2_Zebrafish        60 ------CA----GGCT----G-AAATTGC-----------T-------------------
CNE2_Xenopus         183 ACCGATCACAATGGCATTAGGGAAA-TGCTTGACTTTAGTT----------AGAGAAGCG
CNE2_Chicken         238 AAAGATCACAATGGCATTAGG-AAA-TGCTTGACTTTAGTT----------ACAGGAGCG
CNE2_Mouse           322 GAGGAGCACAAGGGCTTCA-G-AAA-TGCTTGACTTTAGCT----------GGAGGAGTG
CNE2_Human           170 AAAGATCACAAGGGCTTTAGA-AAA-TGCTTGACTTTAGTT----------GGAGGAGCA

                     421 .......430.......440.......450.......460.......470.......480
CNE2_Anolis          294 TAAA--GGCACAAGAACAGATGATGGT-GCCCTAATAGCCCTTTTTAAGAATGACAGGGC
CNE2_ElephantShark   238 TGAGTGGGTACAAGAGATGGTGGCTAT-GCCCTAATAGCCCTTT---AGCGTGTCAGGCC
CNE2_Zebrafish        75 --AG--GGCT----------------T-CTCCTATT-------CTT----------GG--
CNE2_Xenopus         232 TAAG--GGCATAAGAACAGATGATGTTGGCCCTAATAGCCCTTCCTAAGAATGACAGCAC
CNE2_Chicken         286 TAAG--GGCACAAGAACAGATGATGGT-GCCCTAATAGCCCTTTTTAAGAATGACAGGGC
CNE2_Mouse           369 TAAG--GGCACAAGAACAGATGACGGT-GCCCTAATAGCCCTTTTTAAGAATGACAGG-C
CNE2_Human           218 TAAG--GGCACAAGAACAGATGATGGT-GCCCTAATAGCCCTTTTTAAGAATGACAGGGC

                     481 .......490.......500.......510.......520.......530.......540
CNE2_Anolis          351 C--CCACCATTAGCATCCTTTTATATCCTTGGAAATT-GCTCATGCCTCGCTTTTCTGGT
CNE2_ElephantShark   294 CCCCCGCCATTAGCATCCTTTTATATTCTTCAAAATT-GCCCATGCT-GCCTATTCTGGC
CNE2_Zebrafish        95 ---------------TCC---T--ATC-TCAGAGACTTT-------CTCTC---------
CNE2_Xenopus         290 C-CCGGCCATTAGCATTCTTTTCTACGCCTGGAAATT-GCTCATGCCTCGCTTTCCTGCT
CNE2_Chicken         343 C--CCACCATTAGCATCCTTTTATATCCTTGGAAATT-GCTCATGCCTCGCTTTTCTGGT
CNE2_Mouse           425 C--CTGCCATTAGCATCCCTTTATACCCTTGGAAATT-GCTCATGCCTTGCTTTTCTGCT
CNE2_Human           275 C--CAGCCATTAGCATCCTTTTATATCCTTGGAAATT-GCTCATGCCTCGCTTTTCTGGT

                     541 .......550.......560.......570.......580.......590.......600
CNE2_Anolis          408 TCCTATCTGCCTGGC-----TCTTTTTTA----TTTCTCTTTCAATTTCGT-CCGCGCTC
CNE2_ElephantShark   352 TCCTATCTGCCTGCCTGCCCTCTTTTTTTTCTCTCTCCCTTTCAATTCTGT-ACGTGCTT
CNE2_Zebrafish       118 ---TTT--------C-----TTTCTTTTA----TTTCCCTTTCAATTTTCCCTGGCACGC
CNE2_Xenopus         348 CCCTATCTGCCCGAG-----TCTTTTTTA----TTTCTCTTTCAATTTTGT-CCGCGCTC
CNE2_Chicken         400 TCCTATCTGCCTGAC-----TCTTTTTTA----TTTCTCTTTCAATTTCAT-CCGCGCTC
CNE2_Mouse           482 TCCTATCTGCCTGGC-----TCTTTTTTA----TTTCTCTTTCAATTTCGT-CCGCGCTC
CNE2_Human           332 TCCTATCTGCCTGAC-----TCTTTTTTA----TTTCTCTTTCAATTTCGT-CCGCGCTC

                     601 .......610.......620.......630.......640.......650.......660
CNE2_Anolis          458 GGATAAGCGCATGCTAACACAATATGATTGAGCTGTAAAATGGAACGCTGTCGCCTCTAA
CNE2_ElephantShark   411 GGATAAGCGCGTGCTAACACAATATGATTGAGCTGTAAAATGGAATCCTGTCGGCTTTAA
CNE2_Zebrafish       158 GGATAAGCGCGTGCTAACACAATATGATTGAGCTGTAAAATGGAACCCTGTCGACTCTAA
CNE2_Xenopus         398 GGATAAGCGCACGCTAACACAATATGATTGAGCTGTAAAATGGAACGCTGTCGCCTCTAA
CNE2_Chicken         450 GGATAAGCGCATGCTAACACAATATGATTGAGCTGTAAAATGGAACGCTGTCGCCTCTAA
CNE2_Mouse           532 GGATAAGCGCGTGCTAACACAATATGATTGAGCTGTAAAATGGAACGCTGTCGCCTCTAA
CNE2_Human           382 GGATAAGCGCATGCTAACACAATATGATTGAGCTGTAAAATGGAACGCTGTCGCCTCTAA

                     661 .......670.......680.......690.......700.......710.......720
CNE2_Anolis          518 TCTCTTTTATGTAGATATGGAGGTTC--TTCCACACTCCACTTCATTCTTTCTCCATTGT
CNE2_ElephantShark   471 TCTCTTTTATGTAGATACTGTAGTAC--TTCCACACTCTGCTTCATC--CAGTCTATTGT
CNE2_Zebrafish       218 TCTCTTTTATGTAGATACTGAGGTAC--TGCCACACTCCACTTCA-----------TTC-
```

```
CNE2_Xenopus        458 TCTCTTTTATGTAGATATGGCGGCCCCCTTCCAC----------ACTCCCTGCCCATTGT
CNE2_Chicken        510 TCTCTTTTATGTAGATATGGAGGTAC--TTCCACACTCCACTTCATTCTCTCTCCATTGT
CNE2_Mouse          592 TCTCCTTTATGTAGATATGGAGGTTC--G-GCACACTCCACT-----CCCTCTCCATTGT
CNE2_Human          442 TCTCTTTTATGTAGATACGGAGGTAC--T-GCACACTCCACTTCACTCCCTCTCCATTGT

                    721 .......730.......740.......750.......760.......770.......780
CNE2_Anolis         576 TGGCCTTT-ATAGAATGGGTTGCAGAGTAA------TGGAAGCTCACACTGTCAAATAT-
CNE2_ElephantShark  527 AAGA-TGC-ACAGAATGGGCCAGAGAGTAA------TGTAGGCTGA----ATCAAATAT-
CNE2_Zebrafish      264 ----CT-----C--------TGCC------------------------------------
CNE2_Xenopus        508 GGTCTCGC-CT---------TAGCGAG-AA------GGGA--C-----------------
CNE2_Chicken        568 TGGCCTTT-TTAGAATGTGTTGCCGAGTAA------TGGAGGCTCA----GTCAAATAT-
CNE2_Mouse          644 TGGCCTTTCTTAGAACAGGCTGCCGAGGAACGAGAAAGGAGGCTCC----CACAGACAGC
CNE2_Human          499 TGGCCTTTTTTCGAATGGGTTGCCAAGTAA------TGGAGGCTCT----GTCAAATAT-

                    781 .......790.......800.......810.......820.......830.......840
CNE2_Anolis         628 --TAGAGCTAGGTTTCACAGGGTGTGATAAGAAGATTATCAGCCCAGCA--GTCAGATCT
CNE2_ElephantShark  574 --TGAAGCTAGCTTTCAC-GCATACGATAAAAAGATTATCAGTGCAGCA--GTCAGACGT
CNE2_Zebrafish      271 ------------------------------------------------------------
CNE2_Xenopus        532 ------------TTT---AGGGTA-----------TTGGCG-----GCAGTGTCAGAG--
CNE2_Chicken        616 --TAGAGCTAGGTTTCACAGGGTGTGATAAGAAGATTATCAGCCCAGCA--GTCAGATCT
CNE2_Mouse          700 CGAGGAGCTTGGTTTCACAGGGTGTGATAAGGCAATTATCAGCCCAACA--GGCAGATCT
CNE2_Human          548 --TAGAGCTAGGTTTCACAGGGTGTGATAAGACGATTATCAGCCCAACA--GTCAGATCT

                    841 .......850.......860.......870.......880.......890.......900
CNE2_Anolis         684 ATTGTTGTTCTTTGTGTCAGATTAAAATATTCTTCTGTACCGTAACTTCGTTAGCAAAAT
CNE2_ElephantShark  629 ATTGTTGCACTTTGTGTTGGATTAGAATATCCACAT-----GTAACCTCGTTAACAAAAT
CNE2_Zebrafish      271 -------------------------------------------TCC----------
CNE2_Xenopus        559 --GAT----------G--GGATAAGGAGAT------------------------------
CNE2_Chicken        672 ATTGTTGTTCTTTGTGTCAGATTAAAATATTCCGATGTACTGTAACTTCGTTAGGAAAAT
CNE2_Mouse          758 GGGATTGTTCTTTGTGGCTGATTAGGATATTATTATGTCCTGGAATTTCCTTAGCAAAAT
CNE2_Human          604 ATTATTGTTCTTTTTGGTTGATTAAAATATTATTATGTACTGTAACTTTGTTAGCAAAAT

                    901 .......910.......920.......930.......940.......950.......960
CNE2_Anolis         744 ACTACAGGTATTTAAGAAGGCGCTAATGCTGATTTGTTTTTCA-C---TGAGATAATACC
CNE2_ElephantShark  684 GTTGCAGGTGCTTACTC------AGGCCCTGCT---------------------------
CNE2_Zebrafish      274 ------------------------------------------------------------
CNE2_Xenopus        575 ------------------------------------------------------------
CNE2_Chicken        732 ACTGAAGGTGCTTAGGGAGAGACTGATGCTGCTTCATCTCCTGCCTGATGAGACAAAACC
CNE2_Mouse          818 ACAGCGTAGGCTTAGC-------AGGTGGCACT---------------------------
CNE2_Human          664 GCAGAATGTGCTTAGTC------AGATGGTGCT---------------------------
```

CNE3

```
CNE3_Anolis          1 TCCCTATC----TGTTCCTCAAAACATCTGTGGATGTGTCCATTTTATACACACACAAGC
CNE3_ElephantShark   1 GAA----C------ACCCCAG---CAGCCAG--A--T-----------------------
CNE3_Zebrafish       1 CCC-----------A---------------T--A--T-----------------------
CNE3_Xenopus         1 CCC-----------ATC--------A----T--A--T-T-CAC--------CT--CA---
CNE3_Chicken         1 CACCCAT------GC-CCCCAACACAC------ATA-CTACACTGT-----CT-GCCGTT
CNE3_Mouse           1 GGTGA-CC-----GACCCTCGCTCTAACCCC--A--CTCCCTCTT----CCCA--TAGTG
CNE3_Human           1 TTTGCTCCTTTTTGACCCTCTCTCCAACCCT--A--ATCCCACTT----CCCT-TCAGTG

                    61 ........70........80........90.......100.......110.......120
CNE3_Anolis         57 -GATGGTTCTCTCCCCCCCCCCCAATGGTGTCCCCCTTTATCTGCTTCAACCAATCTCCA
CNE3_ElephantShark  21 ---------ACCCTTT------C---TGGCACCCCCTCCATCTGCTTCAACCAATATCCA
CNE3_Zebrafish       8 -------------------------------CCCC--------ACTCCAACCAATCTCCA
CNE3_Xenopus        19 ------------TTGA-C----C--------ATCCCTTTATCTCTCCCAGACAGTCTCCC
CNE3_Chicken        41 -GCACGTT-T--TTTTCCCTCCC---AGTGTCCCCCTTTATCTGCCTCAACCAATCTCCA
CNE3_Mouse          45 -GCTAGT-CACACTTT-C-CCTC---TGTGTTCCCCTTCATCTGCCTCAGCCAATCTCCA
CNE3_Human          52 GGCTGGT-CGCACTTTTT-CCCC---TGCGTCCCCCTTTATCTGCCTCAGCCAATCTCCA

                   121 .......130.......140.......150.......160.......170.......180
CNE3_Anolis        116 AATGAATGCATGCCTATCATGGCCTTAGCTTTGACACTCTGGATATATTGATTAATAGTT
CNE3_ElephantShark  63 AATGCATCACTGTCTATCAGGGTCTCAGCACTGACACTCTGGATGTATTGATTAATAGTT
CNE3_Zebrafish      29 AATGTGGCCTTGTCTGTCACCGCCCTCAGCCTGACACACTGCAAATATTGATTAATAGAT
CNE3_Xenopus        54 AGTGATAGAATGTCTGTCATCTCCTTGGATCTGACACTCTACAGATATTGATTAATAGTC
CNE3_Chicken        94 AATGAATGCATGTCTATCATGGCCTTAGCTTTGACACTCTGGATATATTGATTAATAGTT
CNE3_Mouse          98 AATGAATGCGTGTCTATCCCGGCCTTGGCTTTGACACTCTGGATATATTGATTAATAGTT
CNE3_Human         107 AATGAATGCGTGTCTATCCTGGCCTTGGCTTTGACACTCTGGATATATTGATTAATAGTT

                   181 .......190.......200.......210.......220.......230.......240
CNE3_Anolis        176 GCCCTAAGCTCCTTTTAGCAAATCAAATTTTCACTTTTAGCAAGGATTTCGCCTCTGATT
CNE3_ElephantShark 123 GCCCTAAGCTCCTTTTAGCAAATCAAATTTTCACTTTTAGCGAGGATTTCGGTTCTGATT
CNE3_Zebrafish      89 GCCCTAAGCTCCTTTTAGCAAATCAAATTTTCACTTTTAGCAGGGGTTTCACTCCTGATA
CNE3_Xenopus       114 GCCTGAAGCTCCTTTTAGCAAATCAAATTTTCACTTGTAGCAAGGAACTCACCTCTGATT
CNE3_Chicken       154 GCCCTAAGCTCCTTTTAGCAAATCAAATTTTCACTTTTAGCAAGGATTTCGCCTCTGATT
CNE3_Mouse         158 GCCCTAAGCTCCTTTTAGCAAATCAAATTTTCACTTTTAGCAAGGATTTCGCCTCTGATT
CNE3_Human         167 GCCCTAAGCTCCTTTTAGCAAATCAAATTTTCACTTTTAGCAAGGATTTCGCCTCTGATT

                   241 .......250.......260.......270.......280.......290.......300
CNE3_Anolis        236 TATTCAGCAAAGTGAAATGCACTAAATTGACTTTGACATTCAGGGGAAATGGCTGAAAAA
CNE3_ElephantShark 183 TATTCAGCACAGTGAAATGCAGTAAATTGACTTTGACACTCTGGAAAAACGTCGGAAAAA
CNE3_Zebrafish     149 CATTCAGCAAAGTGAGACACACTAAATTAAGCCTGACACTCAGAGAAAAAGGCTGG----
CNE3_Xenopus       174 TATTCAGCAAAGTGCAATGCACCAAATTGACTTTGACATTCAAG-----TGG--------
CNE3_Chicken       214 TATTCAGCAAAGTGAAATGCACTAAATTGACTTTGACATTCAGGGAAAATGGCTGAAAAA
CNE3_Mouse         218 TATTCAGCAAAGTGAAATGCACTAAATTGACTTTGACATTCAGGGAAAATGGCAGAAAAA
CNE3_Human         227 TATTCAGCAAAGTGAAATGCACTAAATTGACTTTGACATTCAGGGAAAATGGCTGAAAAA

                   301 .......310.......320.......330.......340.......350.......360
CNE3_Anolis        296 CAAAGA--------GGAGAGAAAAGAGATATAA-AT-TGTGTGGCATCTGGATTCACAGA
CNE3_ElephantShark 243 CAATGCTTTCAAGTGGAGAGGAAATAGATATAAAA-TGTGTGGCAGCTTGTTTG----T
CNE3_Zebrafish     205 ---------------------------A--------------------TGGA--------
CNE3_Xenopus       221 ---------------------AAACAGTAACAA-ACATGC--------------------
CNE3_Chicken       274 CAAAGA--------GGGGAGGAAAGAGATATAA-AT-TGTGTGGCAGCTGGAGAG----T
CNE3_Mouse         278 CAAAGA--------GCCCAGGAAAGAGATATAA-AT-TGTGTGGCAGCTGGAGAC----T
CNE3_Human         287 CAAAGA--------GCAGAGGAAAGAGATATAA-AT-TGTGTGGCAGCTGGAGAC----A

                   361 .......370.......380.......390.......400.......410.......420
CNE3_Anolis        346 AGAGGCACCGTTTTCTTTTTCTTTTTTAAAGAGAGATCATTGCTTA-CTTGAAAACATGA
CNE3_ElephantShark 298 TCAGG-------------------------------------------------------
CNE3_Zebrafish     210 -C----------------------------------------------------------
CNE3_Xenopus       239 ----------G-------------------------------------------------
CNE3_Chicken       320 TCAAGCATAAAAGAG--GCAGTTTTCTATAGA-ATATCACTGGTTAATTTGGAAACAT--
CNE3_Mouse         324 TCAGGCACCAGAGAA--ATGGTTTTCTAGAGA-ATTCCA-TG-TCA-TTTAAAAACACTT
CNE3_Human         333 TCAGGCATCCGAGAA--ACAGTTTTCTATAGA-ATACA---G-TCA-TTTAAAAGCACTT
```

CNE4

```
                   181 .......190.......200.......210.......220.......230.......240
CNE4_Anolis        146 ACTGGATTTAGAGCAGACAGAGTCTATACCTACATGACTAGGTCAGCGGAATGA-ACCAG
CNE4_ElephantShark  17 ------------------------------------------------------------
CNE4_Xenopus        20 -------TCTTA---------------------------GAATC--------CAGCC---
CNE4_Chicken        84 -----TTTCCTA---------------------------AAGTCAGCTG----AGACC-G
CNE4_Mouse         138 -----CTTCAGG---------------------------GAATCATGTG----AGCCC-A
CNE4_Human         114 -----TTTCAGA---------------------------GAATCATCTG----AGCCC-A

                   241 .......250.......260.......270.......280.......290.......300
CNE4_Anolis        205 TCTAGTTGCTTGAGCTAAGCTGTCCCCAATTAAATA-CTTTAAAGAGCTTCTACTTTCTC
CNE4_ElephantShark  17 ------------------------------------------------------------
CNE4_Xenopus        35 --------------------------TGAAA--ATGACTCC--------------TTTTC
CNE4_Chicken       107 GCTAAATGCTG----TAA-----TTCTGAGG--ATGCCT--------CCTCTACTTTCTC
CNE4_Mouse         161 ATTAAATCCCA----TCA-----CTCCAGGG--GCATCTCCAAAGAGCCGCTACCTCCTC
CNE4_Human         137 ATTAAATACCA----TAA-----CACTAGGG--GTGTCTCCAAAGAGCAGCCACCTCCTC

                   301 .......310.......320.......330.......340.......350.......360
CNE4_Anolis        264 TCTGGGTGCTGCAGGTTCCCCACCTGCTTAACCTAGTTTGGAATA--GGATTAATTACTT
CNE4_ElephantShark  17 -------------------------ACTTAGCGTGCATTAGAA-G--GGATTAATCACCT
CNE4_Xenopus        53 TT--TAT-AG-TTGTCTTCACTCCCACTTAAGCCACATTGGAAAAAGGGATTAATTACAC
CNE4_Chicken       148 TCTGGATGAGTTAGGCTTCACACCTGCTTAACCTACATTAGAATA--GGATTAATTACCT
CNE4_Mouse         210 TCTATATGAGGAAGGCTTCCCACCTGCTTAACCTGCATTAGGAAA--GGATTAATTACCT
CNE4_Human         186 TCTGTATGAGGGAGGCTTCACACCTGCTTAACCTACATTAGGAAA--GGATTAATTACCT

                   361 .......370.......380.......390.......400.......410.......420
CNE4_Anolis        322 GAGTTACAAGGCCCCCA-CAGGCCATCAGCACGTTTCCAGCAGGG-CACATGCCTTGACC
CNE4_ElephantShark  49 GTACTGAGGAG-TGTTA-AAACACGTCAGCACGTTCCTAACAAGG-TACATGT--TGACC
CNE4_Xenopus       109 GGATAAAGTAGCTGCTAGTGGCCAGTCAGCATGTTTCTGCCAGGGTCACGCAG--TGCCC
CNE4_Chicken       206 GAATTGCGAGGCCCCCT-TAGCCAGTCAGCACGTTTCTGGCAGGG-CACATGG--TGACC
CNE4_Mouse         268 GGGGTGT--GGGCCCCT-CTGCCAGTCAGCAGGTTTGTGACAGGG-CACGTGG--TGGCC
CNE4_Human         244 GAAGTGCTGGGCCTCCT-CCACCAGTCAGCACGTTTGTGCCAGGG-CACATGG--TGGCC

                   421 .......430.......440.......450.......460.......470.......480
CNE4_Anolis        380 TAGGTGC-TGGCACAGGGAAATGGAGCAGAGGGAGATCTAGGTGCTCCAGTCTAAGCAGA
CNE4_ElephantShark 104 TGG---C-T---------------------------------------------------
CNE4_Xenopus       167 CCG--AG-T---------------------------------------------------
CNE4_Chicken       262 TGG--GCCTG-------G------------------------------------------
CNE4_Mouse         322 TGC--AC-TG-CTGAGGGA--TGGAA-GGAGGGAGGC--AGGAGGGGAAGA-GAAGCAGC
CNE4_Human         300 TGG--GC-TG-CAGAGGGA------G-AGAGGGAGAA---------------GGAGCAGC
```

CNE5

```
                   121 .......130.......140.......150.......160.......170.......180
CNE5_Anolis        102 ACTAGCCCTTTTAATGTATCTGAACCATTCTGAAGT-GCCTGCTCTATGGTACATCCTTA
CNE5_ElephantShark  13 ---------------G----------------AAAT-----G------------------
CNE5_Zebrafish      17 -------------------------A--TC--A-------GGCCCTGTG-----TC----
CNE5_Xenopus        42 -------------------------A----AAAGCT-ATAATCTTCATG-TGCTTCCCTA
CNE5_Chicken        48 ACTT--CCAT-TGATACATTTGTACAGTGCTGAGGT-ACAGGCTCCATGTTACATCCATA
CNE5_Mouse         101 TTTTGTCCCTTTGCGG----GGCTCTGCTC--AGATGGTGGGCTCCATGTTGCATCCGCA
CNE5_Human          52 TTTTGTTCTC-TCCGGCAACAGCACAGTTCAGAGGT-GTGGGCTCCATGTTGCGTCCATA
CNE5_Lamprey         9 -------------------------------GA--------GCC----------------

                   181 .......190.......200.......210.......220.......230.......240
CNE5_Anolis        161 CTTGGCTTGTTATCCTCTTATCA-GCCTCTTTGCTTCCCTTTGTTTATTTGAGGAGACAG
CNE5_ElephantShark  19 --------------------------CTCTTT----------------CTG---------
CNE5_Zebrafish      32 -------TG---TCC-------------C---------------------------ACAG
CNE5_Xenopus        71 GTAGGTTTGGGATCCTCTTATCAAAGCTCTTTGCATCCTTTTGTTTATTGAAGAAGACAG
CNE5_Chicken       104 CTTGGCTCGTTATCCTCTTATCA-AGCTCTTTGCATCCCTTTGTTTATTTGAGGAGACAG
CNE5_Mouse         155 CTTGGCTTGTTACCCTCTTCTCA-GGCTCTTAGCTTCCCTTTGTTTATTTGAGGCGACAG
CNE5_Human         110 CTTGGCTTGTTATCCTCTTATCA-AGCTCTTAGCATCCCTTTGTTTATTTGAGGTGACAG
CNE5_Lamprey        14 ----------------------------CT------CC----G------T-----GACAA

                   241 .......250.......260.......270.......280.......290.......300
CNE5_Anolis        220 GAAGACTGTGTGAAGGCTGCTTAGTCAGGGATATCAGCGTTGTTGGCAGGGGAGTGATTG
CNE5_ElephantShark  28 -------------AGGCTGCTTAGTCAGGGATATCACC-CTGTTGGCAG-GCAGTGATTG
CNE5_Zebrafish      42 CCAG-CGG----GGGG------------------CGGCCCTG----------TGTGATTG
CNE5_Xenopus       131 AAAGACGGTGTCGAGGCTGCTTACTCAGGGATATCATGCTTGTTGGCAGAGAAGTGATTG
CNE5_Chicken       163 GAAGACTGTGTGAAGGCTGCTTAGTCAGGGATATCAGCGTTGTTGGCAGGGGAGTGATTG
CNE5_Mouse         214 GAAGACGGCGTAAAGGCTGCTTAGTCAGGGATATCAGCCTTGTTGGCAGGGGAGTGATTG
CNE5_Human         169 GAAGACTGTGTAAAGGCTGCTTAGTCAGGGATATCAGCCTTGTTGGCAGCGGAGTGATTG
CNE5_Lamprey        25 ---------------------------------------------------GAGCGATTG

                   301 .......310.......320.......330.......340.......350.......360
CNE5_Anolis        280 CCAGTTGAA--AAACAAGGCATTATTGCTCATTTTTTCCCTCTTATTAGTTTGATTACAT
CNE5_ElephantShark  73 CCAGGCGAAGAAAACAAGGTATTATTGCACATTTTTTCCCTCTTATTAGTTTGATTACAT
CNE5_Zebrafish      69 CCCGTTGAA-AAAACAAGGCATTATTGCTCATTTT-TCCCTCTTATTAGTTTGATTACAT
CNE5_Xenopus       191 CCAGTTGAA-AAAACAAGGCATTATTGCTCATTTTTCCCCTCTTATTAGTTTGATTACAT
CNE5_Chicken       223 CCAGTTGAA--AAACAAGGCATTATTGCTCATTTTTTCCCTCTTATTAGTTTGATTACAT
CNE5_Mouse         274 CCAGTTGAA--AAACAAGGCATTATTGCTCATTTTTTCCCTCTTATTAGTTTGATTACAT
CNE5_Human         229 CCAGTTGAA--AAACAAGGCATTATTGCTCATTTTTTCCCTCTTATTAGTTTGATTACAT
CNE5_Lamprey        34 CCGCTCGCA--AAACACGGCGTTATTGCGCATTTT--CCTCGTGGTTAGTTCGATCGCAT

                   361 .......370.......380.......390.......400.......410.......420
CNE5_Anolis        338 TTGCAAATCAAATCAATCCATCAGACAT--GATC-A-GGCCTCGTCC-GCATT-TT-A-A
CNE5_ElephantShark 133 TTGCAAATCAAATCAATCCATCAGTCGT--GATC-A-G-CTGTGTTC-AGAAT-TC-A-A
CNE5_Zebrafish     127 TTGCAAATCAAATCAATCCATCAGGCAGTTTGTGAAGGGCCGCGTCCTGCAAT-CTGAAA
CNE5_Xenopus       250 TTGCAAATCAAATCAATCCATCAGACAT--GATC-A-GGCCGCGTCC-GCGTT-TC-A-A
CNE5_Chicken       281 TTGCAAATCAAATCAATCCATCAGACAT--GATC-A-GGCCTCGTCC-GCATT-TT-A-A
CNE5_Mouse         332 TTGCAAATCAAATCAATCCATCAGACAT--GATC-A-GGCCTCGTCC-GCATT-TT-A-A
CNE5_Human         287 TTGCAAATCAAATCAATCCATCAGACAT--GATC-A-GGCCTCGTCC-GCATT-TT-A-A
CNE5_Lamprey        90 TTGCAAATCAAATCAATTCATCAGTTTC--TATC-A-T-CGTCATTA-ATAACACT-C-G

                   421 .......430.......440.......450.......460.......470.......480
CNE5_Anolis        390 GG----AATAGTAATCTCTGTCTAATAAGATGCAAATGTGTCACATCGGTAAGTAACCAA
CNE5_ElephantShark 184 GG----AATGGTAATCTTCTTCTAATAAGATGCAAATGTGTCACATCAGTAAGTAACCAA
CNE5_Zebrafish     186 GGGCCTCCTAGTAATCTCCATCTAATAAGATGCAAATGTGTCACATCAGTAAGTAACCAA
CNE5_Xenopus       302 GC----AATAGTAATCTCCGTCTAATAAGATGCAAATGTGTCACATCGGAAAGTAACCAA
CNE5_Chicken       333 GG----AATAGTAATCTCCGTCTAATAAGATGCAAATGTGTCACATCGGTAAGTAACCAA
CNE5_Mouse         384 GG----AATAGTAATCTCCGCCTAATAAGATGCAAATGTGTCACATCGGTAAGTAACCAA
CNE5_Human         339 GG----AATAGTAATCTCCGTCTAATAAGATGCAAATGTGTCACATCGGTAAGTAACCAA
CNE5_Lamprey       142 CG----GTTTGCAATCTTCTTGTAATAAGATGCAAATGTGTGTTGAAAGCGAGTTACCTA

                   481 .......490.......500.......510.......520.......530.......540
CNE5_Anolis        446 TAAATCATCGTCTTGTCTTTGGCAATCATTAAATATCAGAACCAACAGTCAATTTTTAGT
CNE5_ElephantShark 240 TAAATCATTGGTATGTCATTGGCAATCATTAAATATCAGAATGAACAGTCAATTTTTAGT
CNE5_Zebrafish     246 TAAATCATCGGCGCGTCTACGCCAATCATTAACCATCAGAATGAACAGTCAATTTTTAGT
CNE5_Xenopus       358 TAAATCACGGCCTTGTCTTTGGCAATCATTAAATATCAGAACCAACAGTCAATTTTTAGT
CNE5_Chicken       389 TAAATCATCGTCTTGTCTTTGGCAATCATTAAATATCAGAACCAACAGTCAATTTTTAGT
CNE5_Mouse         440 TAAATCATGGTCTTGTCTTTGGCAATCATTAAATATCAGAACCAACAGTCAATTTTTAGT
CNE5_Human         395 TAAATCATCGTCTTGTCTTTGGCAATCATTAAATATCAGAACCAACAGTCAATTTTTAGT
CNE5_Lamprey       198 TAAATCACCATCCTGTCGGTGGCAATCATTAAATATCAGAATGCACAGTCAATTTTTAGT

                   541 .......550.......560.......570.......580.......590.......600
CNE5_Anolis        506 ATTTTCAATTTGCGATATGCC-GCTGGAATATAAAGATAGATGGACGTAATTACACAA-A
CNE5_ElephantShark 300 ATTTTCAATTTGCGATATGCC-TCTGGAATATAAAGATAGATGGACGTAATTACACAA-A
CNE5_Zebrafish     306 ATTTTCAATTTGTGATATGCT-AGTGGAATATAAAGATAGATGGACGTAATTATACAA-A
CNE5_Xenopus       418 ATTTTCAATTTGCGATATGCC-GCTGGAATATAAAGATAGATGGACGTAATTACACAAAA
```

```
CNE5_Chicken        449 ATTTTCAATTTGCGATATGCC-GCTGGAATATAAAGATAGATGGACGTAATTACACAA-A
CNE5_Mouse          500 ATTTTCAATTTGCGATATGCC-GCTGGAATATAAAGATAGATGGACGTAATTACACAA-A
CNE5_Human          455 ATTTTCAATTTGCGATATGCC-GCTGGAATATAAAGATAGATGGACGTAATTACACAA-A
CNE5_Lamprey        258 ATTTTCAATTTACCGTATGCCTTCTGGAATATAAAGATAGATGGATGTAATTACACAA-A

                    601 .......610.......620.......630.......640.......650.......660
CNE5_Anolis         564 CCAAACACTATTTCAGTTC----AT-TCCAGACAGCAAATTTAGTGGAAGGTATAACTGG
CNE5_ElephantShark  358 CCAAACACTATTTCAGTTC----AT-TCCAGACAGCAAATTTACTGTAAGGAATAACTTG
CNE5_Zebrafish      364 CCAAACTCTATTTCAATCCTCCCATCTCCAGA-AGCAGATTTGCTGGGAGGTAGAACAGG
CNE5_Xenopus        477 CCAAACACTATTTCAGTTC----AT-TCCAGACAGCAAATTTAGTGGAAGGTATAACTGG
CNE5_Chicken        507 CCAAACACTATTTCAGTTC----AT-TCCAGACAGCAAATTTAGTGGAAGGTATAACTGG
CNE5_Mouse          558 CCAAACACTATTTCAGTTC----AT-TCCAGACAGCAAATTTAGTGGAAGGTATAACTGG
CNE5_Human          513 CCAAACACTATTTCAGTTC----AT-TCCAGACAGCAAATTTAGTGGAAGGTATAACTGG
CNE5_Lamprey        317 CCAAACACTATTTCAATTC----AC-CTTGGACAGCAAATTTAGTGTAAGGAATAACATT

                    661 .......670.......680.......690.......700.......710.......720
CNE5_Anolis         619 CTTGTCATCACACTACATTATTTCTGGC------------------------------GG
CNE5_ElephantShark  413 GCTGTCATCACAGTACATTATTCCTCAC------------------------------AG
CNE5_Zebrafish      423 CCTGTCATCTTATTATAGTATTTTTCTTTTCTTTTTTTTTTCTTTTTGGTGGTCTGGAGG
CNE5_Xenopus        532 CTTGTCATCACACTACATTATTTCTGCC------------------------------GG
CNE5_Chicken        562 CTTGTCATCACACTACATTATTCCTGGC------------------------------GG
CNE5_Mouse          613 CCTGTCATCTCCCCGCATTATTCCTGCC------------------------------GG
CNE5_Human          568 CTTGTCATCACACTACATTATTCCTAGC------------------------------GG
CNE5_Lamprey        372 CCTGTCATCACGGCCCATTATTTCATGT------------------------------GG

                    721 .......730.......740.......750.......760.......770.......780
CNE5_Anolis         649 TTCATGCAAAGTTCACAGACTAGCTTCAAATGGACACTGCTATGCAC----------AC-
CNE5_ElephantShark  443 TTCATGCAAAGTTCACAGACAAGCATGAAATGGCCATTTCTTT-----------------
CNE5_Zebrafish      483 TTCAAGCAAAGTTCACAGACAGCCTTGA--------------------------------
CNE5_Xenopus        562 TTCATGCAAAGTTCACAGACAAGCTTCAAATGGACATCGGATTTTTT----------ATT
CNE5_Chicken        592 TTCATGCAAAGTTCACAGACAAGCTTCAAATGGACATTGCTTTTTCT----------A--
CNE5_Mouse          643 TTCATGCAAAGTTCACCGACAAGCTTCAGATGGACATTGCTTTTCCCCCCCCCCCCAACT
CNE5_Human          598 TTCATGCAAAGTTCACAGACAAGCTTCAAATGGACATTGCTTTTTTT--------CAACT
CNE5_Lamprey        402 TTCAGGCAAAGTTCACAAGCAGGCACA-GA------------------------------
```

CNE6

```
                     1 ........10........20........30........40........50........60
CNE6_Anolis          1 TCTTCTAGCCTTCAATTGC-CTCATTAGAAATCTATAGGATGACAGTGGCTGA-AACTGA
CNE6_ElephantShark   1 GCTAACG-----------G-----------------AT------AGC---TG--GA--G-
CNE6_Zebrafish       1 AGC-C-------------G---------------------------------C-TA--A-
CNE6_Xenopus         1 GGAACAAGA---------G-----------------AG-------GA----GC-TA--T-
CNE6_Chicken         1 GAAAAGA-----------GC--------G-------AG-------AGA----GA-AA--G-
CNE6_Mouse           1 TAAATGAGAC--------GCTCTC-TT-GG--GCT---T---G--GGC---TTTTCTCT--
CNE6_Human           1 TAAAATCC----------AC-CCCAT------TCTGGAG--TGAATGA----GC-GA--G-
CNE6_Lamprey         1 CTAAT-------------------------------------------------------

                    61 ........70........80........90.......100.......110.......120
CNE6_Anolis         59 AAAAA-AAGATTGTTAGTCATAATATCCCTACTTAATTTTATGTTGAGTTAGAGGAAAAT
CNE6_ElephantShark  19 --A----GG-----------GAT-GTC-T---------AATGCACAG----------CT
CNE6_Zebrafish      10 --A---------------------------------------------------------
CNE6_Xenopus        20 --A----TG-----------GAA-GCCCT---------AATGC-AGA----------CG
CNE6_Chicken        21 --AGG-AAG-----------GATTTCCTT---------TCTGC-GAG----------CC
CNE6_Mouse          37 ----CTTTT--------TTATAACACCCGA-GCCA---TTGTG--GAGC-AGGAGAAGCC
CNE6_Human          35 -AAGCACAGG---------A-GGTGGCCTT---------CGTGG-GAG----------CA
CNE6_Lamprey         6 ----------------------A-------------------------------------

                   121 .......130.......140.......150.......160.......170.......180
CNE6_Anolis        118 GTTGAATTCTTACTCCTGCAA----AGCTTTCCCTGGTGTCTCATCCTCTCTCCT--CTA
CNE6_ElephantShark  40 G-------CT---------------GTCTCCCGAGACAGCTCATCCTCTC-CTT--TTG
CNE6_Zebrafish      11 ---------------------------------------CAG---CAT----------TT---
CNE6_Xenopus        41 G-------CT--------------CTCTTCCCCAACAGTTTCATCCTTTTTCCTTTCTG
CNE6_Chicken        46 G-------CC---------------AGCTTTCCCAAGTGCCTCATCCTCTCCCCT--CTG
CNE6_Mouse          78 C-AGGAGGCCGCCTCCGTGGGAGCGGCCACTCCCAAGCGTCCCATCCTCT--CCT--CTG
CNE6_Human          64 A-------CT---------------ACCTTTCCCAAGTGTCTCATCCTCTCTCCT--CTG
CNE6_Lamprey         7 --------------------------------------------T---TT------AC--

                   181 .......190.......200.......210.......220.......230.......240
CNE6_Anolis        172 TCATCCTGAGGCAATGTGCCTGCATTTGTG--CGTAGATTTATAGTTTATCTTTATCTGT
CNE6_ElephantShark  75 TCAATTCAAGGCATTGTGGTTGCATTTGTG--CAGGGATTTATAGTTTATCTTTATCTAT
CNE6_Zebrafish      19 ---------------------GGGTTTGTGC-TGTGGATTTATAGTTTATCTTTATCTGT
CNE6_Xenopus        79 TCATCCTGAGGCAATGCGCCTGCGTTTGTG--TGTAGATTTATAGTTTATCTTTATCTAT
CNE6_Chicken        82 TCATCCTGAGGCAATGTGCCTGCATTTGTG--TGTAGATTTATAGTTTATCTTTATCTAT
CNE6_Mouse         133 TCATCCCCAGGCGACGCA-CTGCATTTGTGTGTGCAGATTTATAGTTTATCTTTATCTCT
CNE6_Human         100 TCATCCCCAGGCAATGTGCCTGCATTTGTGTGTGTAGATTTATAGTTTATCTTTATCTAT
CNE6_Lamprey        12 ---------------------------TGT----GAA-----AT----------------

                   241 .......250.......260.......270.......280.......290.......300
CNE6_Anolis        230 GGCTTCATCGGTAGGTAGCTATTATAGCCGCCTTGCCTGGATATTGTTTGGGCCTGATAG
CNE6_ElephantShark 133 TGCTTCATCTGTAGGTAGCTATTATAGATGCCTGGGCTGGTTATTGTTTGGGGGTGATAG
CNE6_Zebrafish      57 TGCTTCATCTGTGGGTAGCTATTACGCCAGCCTGGCCAGCTTATTGTTTGGGCCTGATAG
CNE6_Xenopus       137 GGCTTCATCTGTAGGTAGCTATTATAGCTGCCTTGCTTGGATATTGTTCGGGCCTGATAG
CNE6_Chicken       140 GGCTTCATCTGTAGGTAGCTATTATAGCCGCCTTGCCTGGATATTGTTTGGGCCTGATAG
CNE6_Mouse         192 GGCTTCATCTGTAGGTAGCTATTAGAGCCGCCTCGCCCGGATATTGTTTGGGCCTGATAG
CNE6_Human         160 GGCTTCATCTGTAGGTAGCTATTATAGCTGCCTCGCCTGGATATTGTTTGGGCCTGATAG
CNE6_Lamprey        20 --------------------AT--------------------------------------

                   301 .......310.......320.......330.......340.......350.......360
CNE6_Anolis        290 ATCTGTCTGTAGTTCATCAAAGGGAAGCTGAGTGGCT-AAAAGCCACTGGGTCCAGCTGA
CNE6_ElephantShark 193 ATCTGTCTGTACTTCATCAAAG-AAACTGGAGTAGCT-AAAAGCCATTGGGTGAAGCTTA
CNE6_Zebrafish     117 ATCTGTGTGCAGTCCATCACTT-GGAGCCGTGCGCCGGAAAGCCATCG-GCCGCCTGA
CNE6_Xenopus       197 ATCTGTCTGTAGTTCATCAAAGGGAAGCAGAGTGGCT-AAAAGCCGTTGGGTCCAGCTGA
CNE6_Chicken       200 ATCTGTCTGTAGTTCATCAAAGGGGAGCTGAGTGGCT-AAAAGCCATTGGGTCCAGCTGA
CNE6_Mouse         252 ATCTGTCCGTAGTTCATCAAAGGGGAGCCGAGTGCCT-AAAAGCCATCGAGTCCAGCTGA
CNE6_Human         220 ATCTGTCTGTAGTTCATCAAAGAGGAGCCTAGTGCCT-AAAAGCCATTGGGTCCAGCTGA
CNE6_Lamprey        22 ------------------------G---------------AGCCGTC------------

                   361 .......370.......380.......390.......400.......410.......420
CNE6_Anolis        349 GGATGAAATACGAGCCATCAGCCTTGGTAATGGCTGTAAAATTTCATAATTACACGGCCT
CNE6_ElephantShark 251 TGATGAAATACGAGCCATCAGGCTCAGTAATGGCTGTAAAATTTCATAATTACATGTCTT
CNE6_Zebrafish     175 TGATGAAATACGAGCTATCAGCCTCAGTAATGGCTGTAAAATTTCATAATTACACGGCCT
CNE6_Xenopus       256 TGATGAAATACGAGCCATCAGCCTTGGTAATGGCTGTAAAATTTCATAATTACACGGCCT
CNE6_Chicken       259 GGATGAAATACGAGCCATCAGCCTTGGTAATGGCTGTAAAATTTCATAATTACACGGCCT
CNE6_Mouse         311 GGATGAAATACGAGCCATCAGCCTTGGTAATGGCTGTAAAATTTCATAATTACACGGCCT
CNE6_Human         279 GGATGAATTACGAGCCATCAGCCTTGATAATGGCTGTAAAATTTCATAATTACATGGCCT
CNE6_Lamprey        30 -------------GCCATC------TGTAATGGCTGTAAAATTTCATAATTACATGTCCT

                   421 .......430.......440.......450.......460.......470.......480
CNE6_Anolis        409 TTATTACATTGCATAATGATCCTGAAGCAGTATGGAACAATTAATTAAGATTTTAAACCA
CNE6_ElephantShark 311 TTATTACATTGCATAATGATCCTGAAGCAGTATGAAACAATTAATTAAGATTTTGAACCA
CNE6_Zebrafish     235 TTATTACATTGCATAATGACCCTGAAGCAGTATGGAACAATTAATTAAGATTTTCAGCCG
CNE6_Xenopus       316 TTATTACATTGCATAATGATCCTGAAGCAGTATGGAACAATTAATTAAGATTTTCAAGCG
```

```
CNE6_Chicken        319 TTATTACATTGCATAATGATCCTGAAGCAGTATGGAACAATTAATTAAGATTTTCAACCG
CNE6_Mouse          371 TTATTACGTTGCATAATGATCCTGAAGCAGTATGGAACAATTAATTAAGATTTTCAACCG
CNE6_Human          339 TTATTACATTGCATAATGATCCTGAAGCAGTATGGAACAATTAATTAAGATTTTCAACCG
CNE6_Lamprey         71 TTATTGCATTGCGTAATGATCCTGAAGCGGCGCAAAAGAA-TAATTAA--TT--------

                    481 .......490.......500.......510.......520.......530.......540
CNE6_Anolis         469 TGGCTCTTTCAGAAATTAAAAGGTGCTAGTGGTTGAAAATGAGGGGAAACGTCAGGTCC
CNE6_ElephantShark  371 TGGCTTTTT-TGAAATTAAAAGGTACTCG-GTCTGTGAAATG--GGGGAGAGGCTTCCTC
CNE6_Zebrafish      295 TGGCTTTTGCAGAAATTAAAAGGTGCTCATTGCCGTGGAATG--GGGTAATGTCTTGTCT
CNE6_Xenopus        376 TGGCTCTTTCAGAAATTAAAAGGTGCCAGCGACTGAGAAACA--GGGAAATGTCTGTCTC
CNE6_Chicken        379 TGGCTCTTTCAGAAATTAAAAGGTGCTAGTGGCTGAGAAATG--GGGAAGTGTCTTGTCC
CNE6_Mouse          431 TGGCTCTTTCAGAAATTAAAAGGTACTGGGAGCTGAGAAATG--GGGATCTGTCTTGCCC
CNE6_Human          399 TGGCTCTTTCAGAAATTAAAAGGTGCTAGTAGCTGAAAAATG--G-GATCTGTCTTGTCC
CNE6_Lamprey        120 ---------AGGAGATTATGA----------GCCGTA----------------------

                    541 .......550.......560.......570.......580.......590.......600
CNE6_Anolis         529 TAAAAGCATTAGGGATGCTTAAGAA-GTACTTCTAGGGTACTCTCTCTGGAAAGTCACAA
CNE6_ElephantShark  427 TA-AAGCATGAAGGAGACGG--------G--T----------------------------
CNE6_Zebrafish      353 CGGAAGCATTAAGG-CGCGTTT--------------------------------------
CNE6_Xenopus        434 TAAAAGCATTAGCAATGCTCTTGAA--TATGTGCC--GTG--------------------
CNE6_Chicken        437 TAAAAGCATTAGGGATGCTTAAGAA-GTACTT----------------------------
CNE6_Mouse          489 TGGAAGCATTAGGGATGCTAAAGGAAGGCTTG---GCTG--------------------
CNE6_Human          456 TAAAAGCATTAGGGATGCTTAGGAA-ATACTTTCCAATTG--------------------
CNE6_Lamprey        138 ------------------------------------------------------------

                    601 .......610.......620.......630.......640.......650.......660
CNE6_Anolis         588 ATACCAAGAAGTACTTCTTAGGAGAGCTAATGTTAGGGGT-A-A-CTATGAACTGGCTTT
CNE6_ElephantShark  448 -------------------------------GTTCAGGG--A-A-------TC-------
CNE6_Zebrafish      374 ---------------------------------TAAGCGCGAGA-C-----TCC------
CNE6_Xenopus        470 -----------CCCCTCT------G----T-CTTTACAGTATTA-C--------------
CNE6_Chicken        468 ---------------C--------------------------------------------
CNE6_Mouse          526 -----------CATACTT------ATTTTTCCTTAAGAGGAA-AGC----ACCT------
CNE6_Human          495 -----------CACTTTT------ATTTTTTGTTAAGAGG-A-A-C----ATCC------
CNE6_Lamprey        138 ------------------------------------------------------------

                    661 .......670.......680.......690.......700.......710.......720
CNE6_Anolis         645 GATCTGGGTTT-TAAGTTTCAATCAGG-GTTTGAAAGTGATCAGAA-CAGATGCCAGCAG
CNE6_ElephantShark  460 ----TGA-TT--------------------T------------A------TTG-------
CNE6_Zebrafish      389 ---CTG--TC--------------------T------------G------TCT-------
CNE6_Xenopus        493 ---ATGG-TC------------------A-T-----------AG------TTG---G---
CNE6_Chicken        469 ---CTG-------------------------------------G------------G---
CNE6_Mouse          558 C--TTGG-TTGCGGAG-------CACGCATT-----------AGTATCCCTTGC--CT--
CNE6_Human          525 G--CTA----------------------GTT-----------AG--------G---G---
CNE6_Lamprey        138 -----GA-TT-------------------C----------------------G---G---

                    721 ......
CNE6_Anolis         702 CTTTTA
CNE6_ElephantShark  470 --AGCC
CNE6_Zebrafish      399 -GCGTG
CNE6_Xenopus        507 -ATAAT
CNE6_Chicken        474 --TACT
CNE6_Mouse          593 -ATTTC
CNE6_Human          536 -AACAT
CNE6_Lamprey        145 -AGAAT
```

CNE7

```
                   121 .......130.......140.......150.......160.......170.......180
CNE7_Anolis         87 GAC---TAGTCACATAATGTGTTATGCTTTTGAGTGTTTAGTGGCATAGTAAGTGTTCTC
CNE7_ElephantShark  10 --------------------TC--------------------------------------
CNE7_Zebrafish      21 ---------------------------------------------AAAATG---------
CNE7_Xenopus        28 -----------------------------------------AGAGAAAATAAGA------
CNE7_Chicken        50 GACCGGTTATCATGTAAGGTCCTATCAGTCTAAATATTTTATGAGAAGCTAAGGTTTCCC
CNE7_Mouse         115 GAC-AATCATCACATAACGTTTTACAGTTCTAAATATTTCATGGGAGACTAAAACTTCCC
CNE7_Human         103 GAC-AATCATCATATAATGTTTTACGGATCTAAATATTTCATGGGAGACTAAAACTTCCC
CNE7_Lamprey        29 -------------G----------------TAAATGTT----G--A---T---A-----T

                   181 .......190.......200.......210.......220.......230.......240
CNE7_Anolis        144 TTGGGTGTCTTTTAATTATAAACTTTTGCCAGAAAATTTGGAACCGATTATGTGCTTCAT
CNE7_ElephantShark  12 TT--------------------------GCA----------AAAG---TCTGTG------
CNE7_Zebrafish      27 ------------------TAAATAT-----------T---------GATATTT-------
CNE7_Xenopus        41 --------------A-AAAAAACAT-----------TTTGAAAATGAATATTTGTGT---
CNE7_Chicken       110 CTGGGCACCT-CTAATAATGAACTTTCGGCAGGAAATTTAGAAAGGATTATGTGTTT---
CNE7_Mouse         174 TTGGGCCTCC-CCAATAATAAACTTTCAGCAGAAGAGTCAAAAAGGCTGATGTACTT---
CNE7_Human         162 TTGGGCCTCC-CCAATAATAAACTTTCAGCAGAAAATTTGGAAAGGATTATGTGCTT---
CNE7_Lamprey        43 TTGGGCAC---------ATA-------------AAAGTC--------------GCT----

                   241 .......250.......260.......270.......280.......290.......300
CNE7_Anolis        204 TCAAGGAGGACCTGGCTGCTTCAAGTCAATTCTTTTGTTGAGTCATTTATTCCCTTAATA
CNE7_ElephantShark  27 --------------------------------TCTCGTTGA-------------------
CNE7_Zebrafish      42 -CA-------------------------------TTGTT---------------------
CNE7_Xenopus        72 -CAAGAGGGGATTAGCTTTGTAAAGTCAATTTCCTTTTCGGGAAATGTATTCCCTTAATA
CNE7_Chicken       166 -CAAGGAGGAGCTGGCTGCTCGAAGTAAATTCTCTTGTTGAGTCATTTATTCCCTTAATA
CNE7_Mouse         230 -CAAGGAGGAGTCTGCTGCTCCCAGAGAATTCTCTCACTGAGGCATTTAGCCCCTTAATA
CNE7_Human         218 -CAAGGAGGAGCTGG-TGCTCAGAGTAAATTCTCTTGTTGAGGCATTTAGTCCCTTAATA
CNE7_Lamprey        63 ------------------------------TCCC-CG-TGTGCCACTTACCGCCC-----

                   301 .......310.......320.......330.......340.......350.......360
CNE7_Anolis        264 GGAATTTTCTGATCGGCTGTCATTCTTGTTCACACACTGGGTGTCACTGCCTCTAAAACA
CNE7_ElephantShark  36 ---C---TCTGA---------------------------------------------TCA
CNE7_Zebrafish      49 GGAAA--------TGGCTGTGGT-------------------------------------
CNE7_Xenopus       131 GGACTTTTCCGATCCACTGTCATTCTTGTTCACACACTGGGTGTCACTGCCTTTAAAACG
CNE7_Chicken       225 GGAATTTTCTGATCTGCTGTCATTCTTGTTCACACACTGGGTGTCACTGCCTCTAAAACA
CNE7_Mouse         289 TGAATTTTCTGATCGGCTGTCACCCTCACGGGTGCGCCGAGCGTCACTGCCTCCGAAGCA
CNE7_Human         276 GGAATTTTCTGATCTGCTGTCATTCTTACTCACACACCGAGTGTCACTGCCTGTAAAACA
CNE7_Lamprey        86 -----TCCCTGA---------------------------------------------ACG

                   361 .......370.......380.......390.......400.......410.......420
CNE7_Anolis        324 CTTGTCATTCTTTAATGGAAACAAAATAGTCATTGCTGTCAGAGCTGCAATGTCATTCCC
CNE7_ElephantShark  45 CTTGGCATTCCTTAATGGAAACAAAATAGTCCTCCCAGTAAGAAGTCCAATGTCATTTCA
CNE7_Zebrafish      64 -----------------------------------------------TAATGTCACTCCA
CNE7_Xenopus       191 CTTGTCACTCTTTAATGGAAACAAAACAGTCTTTGCAGTCGGTGCTGCAATGTCATTCCC
CNE7_Chicken       285 CTTGTCATTCTTTAATGGAAACAAAATAGTCATTGCAGTCAGAGCCGCAATGTCATTCCA
CNE7_Mouse         349 CTTGTCATTCTTTAATGGAAACAAAACAGTCATTGCAGCCAGAGCCACGGTGTCATCTCA
CNE7_Human         336 CTTGTCATTCTTTAATGGAAACAAAATAGTCATTGCGGCCAGAGCCGCAATGTCATTCCA
CNE7_Lamprey        96 CT---C--------------ACAA-------------------GCTGCGA-----TTC--

                   421 .......430.......440.......450.......460.......470.......480
CNE7_Anolis        384 CTACCCCTTGCTGCGTCGGCAATAGTGAAAATGACAGAGCGGCTCTCAGTAGGGGTAATT
CNE7_ElephantShark 105 CTACCCCATGCTGTCTTAGCAATAGTCA-AATGACAGAGTGATTCTCAGTAGGAGTAATT
CNE7_Zebrafish      77 T---------CTG------------TG---------GAGC--CTCT--G-----------
CNE7_Xenopus       251 TTACCCCTTGCTGCGTCGGCAATCGTGAAAATGACAGAGCGCCTCTCGGTAGGGGTAATT
CNE7_Chicken       345 CTACCCCTTGCTGCGTCGGCAATAGTGAAAATGACAGAGCGGCTCTCAGTAGGGGTAATT
CNE7_Mouse         409 CAGCCCCTTGCTGCGTCGGCAATAGTGG-AATGACAGAGAGGCCCTCGGTAGGGGTAATT
CNE7_Human         396 CTACCCCTTGCTGCGTCGGCAATAGTGAAAATGACAGAGCAGCTCTCAGTAGGGGTAATT
CNE7_Lamprey       113 ---------------------------------------------GCGG------TGCTT

                   481 .......490.......500.......510.......520.......530.......540
CNE7_Anolis        444 AAAATGTAAATATTGATATTTTATTATTTGAAATTACTTTCCAGTGCGGCATTTAATATC
CNE7_ElephantShark 164 AAAATGTAAATATTGATATTTTATTATTTGAAATTACTTTCCAGTACAGCATTTAATGTT
CNE7_Zebrafish      92 ---------CCATGGATGCTTTATT----------ACAGTCCGGGG--------AGCAGC
CNE7_Xenopus       311 AAAATGTAAATATTGATATTTTATTATTTGAAATTACTTTCCAGTGCGGCATTTAATATC
CNE7_Chicken       405 AAAATGTAAATATTGATATTTTATTATTTGAAATTACTTTCCAGTGCGGCATTTAATATC
CNE7_Mouse         468 AAAATGTAAATATTGATATTTTATTATTTGAAATTACTTTCCAGTGCGGCATTTAATATC
CNE7_Human         456 AAAATGTAAATATTGATATTTTATTATTTGAAATTACTTTCCAGTGCGGCATTTAATATC
CNE7_Lamprey       122 ----------------------------CAAAATTAG-TGCCAGCG--------------

                   541 .......550.......560.......570.......580.......590.......600
CNE7_Anolis        504 TCTCCATCTGTGCGGCTCTGTTTAGCGGTCACTTTAGTGCAGCTCTGGGATGCCAAGTGG
CNE7_ElephantShark 224 GCTCCATCTGTGCAGCTCTGTTTAGTGGTCACTTAAGTGCAGCTCTGGGACATGAAGCAG
CNE7_Zebrafish     125 CGCCCTTCT-------------CCGCCGCCACC--AG-----------------------
CNE7_Xenopus       371 TCTCCATCTGTGCCGCTCTGTTTTGTGGTCACTTTAGTGCAGCTCGGGGATGGCGGGGAA
```

```
CNE7_Chicken          465 TCTCCATCTGTGCGGCTCTGTTTAGCGGTCACTTTAGTGCAGCTCTGGGATGCCAAGCAG
CNE7_Mouse            528 TCTCCATCTGTGCGACTCTGTTTAGCGGTCACTTTACTGCAGCTCTGGGAGTCAGAGCGT
CNE7_Human            516 CCTCCATCTGTGCAGCTCTGTTTAGCGGTCACTTTAGTGCAGCTCTGGGATACCAAGCAG
CNE7_Lamprey          139 ----------------------TAGCGATCGC--------CG----------CCAAA---

                      601 .......610.......620.......630.......640.......650.......660
CNE7_Anolis           564 CGGCTTGATCC-CCGTTATGTCAAAATGCTTGTTGCTGCTTAATTTTGATATCTAATTAA
CNE7_ElephantShark    284 CAGTTCAATC--CTGTCATGTCAAAATGCTTGTTCCTGCTTAATTTTGATATCTAATTAA
CNE7_Zebrafish        147 -GGCA-----------C------ATGTGCTTGTTGCTGCCTCGTTTCAATACGCAATTAA
CNE7_Xenopus          431 CGGCTTGATCCCCCGTTATGTCAAAATGCTTGTTGCTGCTTAATTTTGATATCTAATTAA
CNE7_Chicken          525 CGGCTTGATCC-CCGTTATGTCAAAATGCTTGTTGCTGCTTAATTTTGATATCTAATTAA
CNE7_Mouse            588 AGGCTTGATCC-CCGTTATGTCAAAATGCTTGTTGCTGCTTAATTTTGATATCTAATTAA
CNE7_Human            576 CGGCTTGATCC-CCGTTATGTCAAAATGCTTGTTGCTGCTTAATTTTGATATCTAATTAA
CNE7_Lamprey          156 --ACCCAAACT-C-------------TGTTTCTCCCAGC--AATTCTGGCCTCTAATTAG

                      661 .......670.......680.......690.......700.......710.......720
CNE7_Anolis           623 GTGGAAAAGTACAGTCCACACTGTAATCCATGGCATCTTAACCAGCTGCTTGGGTATATT
CNE7_ElephantShark    342 GTGGAAAAGTGCAGTCCATACTGTAATCCA-GGCATCTTAAACAGCTGCTTGGGTATATT
CNE7_Zebrafish        189 GTGGAAAAGTACAGTCCGAACAGTAATCCATGCTATCTTAAGCAGCTGTCTGGGTATATT
CNE7_Xenopus          491 GTGGAAAAGTACAGTCCACACAGTAATCCATGGCATCTTAACCAGCTGCTTGGGTATATT
CNE7_Chicken          584 GTGGAAAAGTACAGTCCACACTGTAATCCATGGCATCTTAACCAGCTGCTTGGGTATATT
CNE7_Mouse            647 GTGGAAAAGTACAGTCCGCACTGTAATCCATGGCATCTTAACCAGCTGCTTGGGTATATT
CNE7_Human            635 GTGGAAAAGTACAGTCCACACTGTAATCCATGGCATCTTAACCAGCTGCTTGGGTATATT
CNE7_Lamprey          198 GC---AA---ACAGGCGA-AC---GATCCAA-GCATCCTGAACAGCTGCTCCAGTATATT

                      721 .......730.......740.......750.......760.......770.......780
CNE7_Anolis           683 TAGAATTAATCTCCACTATGGAATCAT-CCTAATGTATGCAATTAAGAGTCTGCCGATGG
CNE7_ElephantShark    401 TAGAATTAATCTTGGCTATG-AATTGT-CTTAATGTATGCAATTAAGAGTCTGTGCATTC
CNE7_Zebrafish        249 TAGAATTAATCTCCACCACC-AATCGGCCTTAATGTATGCAATTAAGAGTCTGCCGATTG
CNE7_Xenopus          551 TAGAATTAATCTCCACTATGGAATCAA-CCTAATGTATGCAATTAAGAGTCTGCCGATGG
CNE7_Chicken          644 TAGAATTAATCTCCACTATGGAATCAT-CCTAATGTATGCAATTAAGTGTCTGCCGATGG
CNE7_Mouse            707 TAGAATTAATCTCCACTATGGAATCAT-CCTAATGTATGCAATTAAGGTTCTGCCGATGG
CNE7_Human            695 TAGAATTAATCTCCACTATGGCATCAT-CCTAATGTATGCAATTAAGAGTCTGCCGATGG
CNE7_Lamprey          247 TAGAATTAATCTCGGCTCAG-AATAG--TCTAATTTATGCAAT----GGTCTGG------

                      781 .......790.......800.......810.......820.......830.......840
CNE7_Anolis           742 CTAATTAGGTGTATTAGAATCAGGCA-GCTTCCACCCCC-----CCCC--TTTCT-TTCT
CNE7_ElephantShark    459 CTAATTAGGTATATTAGAATCATGCA-GCTTACCA-----------------T-CC-CAGT
CNE7_Zebrafish        308 CTAATTAGGTGCATTAGAATCAAGAA-GCC----------------------A-TGC-
CNE7_Xenopus          610 CTAATTAGGTGTATTAGAACCAGGCA-GCTTTCTAT-----------C--TTTCA-TTCT
CNE7_Chicken          703 CTAATTAGGTGTATTAGAATCAGGCA-GCTTCCTT------------T--TTTTT-CTCT
CNE7_Mouse            766 CTAATTAGGTGTATTAGAATCAGGCA-GTGTTTTTTTCTCCCCCTCCCCCTCTCT-CTCT
CNE7_Human            754 CTAATTAGGTGTATTAGAATCAGGCA-GCTTTTTTTTTT-----TTTC--CTCCATTTCT
CNE7_Lamprey          294 --AATTACG--------------GCATGC-------------------------------
```

CNE8

```
                    181 .......190.......200.......210.......220.......230.......240
CNE8_Anolis         179 GGAAAAAATGTTGACAGGGTGGGGGAGAG-AGACAT-AACC-AGCAAATCAATGCCATTG
CNE8_ElephantShark    9 ------------------------------------------------------------
CNE8_Zebrafish       20 ------------------------------------------------------------
CNE8_Xenopus         25 ------------------------------------------------------C-----
CNE8_Chicken        176 GAAAGAAAGAAAAAAAGGAAGG--GAGAAAATCTGT-AACC-AGCAAATCAATGCTATTG
CNE8_Mouse           41 G-AGAAA----CTATAC-------TTCAAAACCCGAGAACG-AGCAAATCAATACCGTGA
CNE8_Human          155 G-AAAAA----CTACAG-------TTCAAAAACTGT-GATCAGGCAAGTCAATGCCATGA

                    241 .......250.......260.......270.......280.......290.......300
CNE8_Anolis         236 TTTATGGCTATTTACAAGAAACTGCGTGGATTTACAGTGCCTTGCATTCGGCAATGTA-G
CNE8_ElephantShark    9 ------------------------------------------------------------
CNE8_Zebrafish       20 ------------------------TGAGGCC----------TGGTGG------------T
CNE8_Xenopus         26 -------------------TACC-------------------------------------
CNE8_Chicken        232 TTAACTGATATTA-TGAGACACCCTGTAGATTTACAATGC-TTGCATGCAGCCACAT--A
CNE8_Mouse           88 TTAACTGATACTA-ACAGATACCCTGAGGATTTAA-ATGT-TGGCGTTCTGTTTCGAGCT
CNE8_Human          202 TTAACTGATATTA-ACAGATGCCCTGAAGATTTAAAATGC-TTGCTCTCTATTTCAAG-A

                    301 .......310.......320.......330.......340.......350.......360
CNE8_Anolis         295 AAACGGTACAGAAAAGAATGCCTAGCAGGGGTTAAGTTCTTATGCCTGCACTGCAGCCTA
CNE8_ElephantShark    9 ------------------------------------------------------------
CNE8_Zebrafish       34 GAGCAGTC---------CTGCCCGGTAGGGGTTAAGTTCGCATGGTTGCACCGCAGCCTC
CNE8_Xenopus         30 ------------GGAGAATGCCTGAGAGGGGTTAAATTCATATGCCTTCATTGCAGCCTA
CNE8_Chicken        288 AACCCGTGCTGAAAAGAATGCCTACCAGGGGTTAAATTCATGCGCCTGCACTGCAGCCTA
CNE8_Mouse          145 AAGCAGCTGCCAGGCGAATGCCTAGCAGGGGTTAAACCCAAATGCCTGTGCCGCAGCCTA
CNE8_Human          259 AAGCAGCCGAGAAGAAAATGCCTAGCAGGGGTTAAACCCAAATGCCTGCACTGCAGCCTA

                    361 .......370.......380.......390.......400.......410.......420
CNE8_Anolis         355 TTTCTGTCAACACTGGACTTGGGAAACAAACATTTATGCTTTGTGAAATGAAAGTGTATT
CNE8_ElephantShark    9 TT----TCAG------------------------------------AATGAAAGTGCC-T
CNE8_Zebrafish       85 TTTCTGTCAGC-CTGT---------------------GCT--------------------
CNE8_Xenopus         78 TTTCTGTCAACACTCGGCTACAAAAATAAACATTTATGTTTTGTGAAGTGAAAGTGTATT
CNE8_Chicken        348 TTTCTGTCAACACTCAGCTCAGGAAATAAACATTTATGCTTTCTGAAATGAAAGTGTATT
CNE8_Mouse          205 TTTCTGTCAGCACTCCACTCAGGAAATAAACATTTATGCTTTCTGAAATGAAAGTGTATT
CNE8_Human          319 TTTCTGTCAACACTCTGTTCAGGAAATAAACATTTATGCTTTCTGAAATGAAAGTGTATT

                    421 .......430.......440.......450.......460.......470.......480
CNE8_Anolis         415 TTTGAAGACTAGCCGTCAGATATT------TTTT------GCCCTTCACACTTTCAAATG
CNE8_ElephantShark   28 TTTAAAGACTAACTGTCATATATATATTTTTTTT------GCCCTTCACACAGTCAAATA
CNE8_Zebrafish      103 -------ACTCGCT--------------------------G--CT--------TC-AATG
CNE8_Xenopus        138 TTTGAAGACTACCCATCAGATATT------TTTTTTTTTTGTCCTTCACACTTTCCCG--
CNE8_Chicken        408 TTTGAAGACTAGCCGTCAGATATT------TTTT------GCCCTTCACACTTTCAAATG
CNE8_Mouse          265 TTTGAAGACCAGCCGTCAGATAGT------TTTT------GCCCTTCACACTTTCAAATG
CNE8_Human          379 TTTGAAGACTAGCCGTCAGATATT------TTTT------GCCCTTCACACTTTCAAATG

                    481 .......490.......500.......510.......520.......530.......540
CNE8_Anolis         463 TTTAGCCTTT---TTTGTAAGTTGACTGCAAAGTGCATCAGTTTGCCAGCATCCCTTTTT
CNE8_ElephantShark   82 TTTA--------------------------------------------------------
CNE8_Zebrafish      119 CTTATT---T---CTT---------CTGAAAAGC--------------------------
CNE8_Xenopus        190 ------------------------------------------------------------
CNE8_Chicken        456 CTTAGCCTTT---TTTGTAAGTTGACTGCAAAGTGCATCGGTTTGCTAGGC---------
CNE8_Mouse          313 CTTAGCCTTTTTTTTTGTACATTGACTGTAAAGTGCATAAGTTTGCCAGGC---------
CNE8_Human          427 CTTAGCCTTT---TTTGTAAGTTGACTGCAAAGTGCATCAGTTTGCCAGGC---------
```

CNE9

```
                     181 .......190.......200.......210.......220.......230.......240
CNE9_Anolis           39 GATTTTATTGCCTTTTGCTTAATTAAATGCCTCAGAAGTGTGTGAAACATTGTTATAGTT
CNE9_ElephantShark     7 ---------------------------------GG--------CA---------------
CNE9_Zebrafish        12 ----------------GAT--------------GAAAAGGAGGGA------------G--
CNE9_Xenopus          38 CCCCCCCCAG---TGAGGTTTTT-TTATTTTTCGGAAAGGTCCG------------CGGA
CNE9_Chicken          40 GATTTTATTGCTTTTTGCTTAATTAAACTCCTCGGAAGAGTGTGAAACATTGTTATACCT
CNE9_Mouse           162 AATTTTATTGC-TTTTGCTTAATTAAACCCCAAAGAAATGTGGCAGACATTGTTGTTGTT
CNE9_Human           161 AATTTTATTGTTTTTTGTTCAATTAAACCCCAAAGAAATGTGCGAAATGTTGTTATGGCT
CNE9_Lamprey          13 ----CGGCAGCCTTTTGC------------------------------------------

                     241 .......250.......260.......270.......280.......290.......300
CNE9_Anolis           99 GAAA-AAATGTTACTTTGTAAATCGTAGGGACAAAAG-GGCTCCACTGCAACTTTAAAGT
CNE9_ElephantShark    11 G-------------------------------------------------ACTTTGGAGT
CNE9_Zebrafish        28 GAAA-A---------------------------AAA---------ATGCATCTTTAAAGT
CNE9_Xenopus          82 GAGA-AATTGTTCATTTGTAAATCAC--GGACAAAAGAGGCCGCGAAGAATCTCTAAAGT
CNE9_Chicken         100 GAAA-AATTGTTAATTTGTAAATCACAGGGACAAAAG-GGCTCCACTGCAACTTTAAAGT
CNE9_Mouse           221 GAAAAAAATGTTAATTTGTAAATCACAGGGACAAAGG-GGCCCC-GTGCAACTTTAAAGT
CNE9_Human           221 GTAA-CATTGTTAATTTGTAAATCACAGGGACAAAGG-GGGCCCGGTGCAACTTTAAAGT
CNE9_Lamprey          27 ----------------------------------------------------------CG

                     301 .......310.......320.......330.......340.......350.......360
CNE9_Anolis          157 TTCCGTGCACGTAAATGTCAATA-CAGTGCCTTCTCCATCATCAGCACTAAATTCACATT
CNE9_ElephantShark    22 TTCTGCGCACGTAAATGTCAATA-CATTGCCTTCTCCATCATCAGCACTAAATTCACTTT
CNE9_Zebrafish        51 TTGCCCTCGCGTAAATGTCAATA-GAGAACCTTTTCCATCATCAGCAGTAAATTCACATT
CNE9_Xenopus         139 TTCCGCGCACGTAAATGTCAATA-TAGTGTCTTCTCCATCATCAGCACTAAATTCACATT
CNE9_Chicken         158 TTCTATGCACGTAAATGTCAATA-GAGTGCCTTCTCCATCATCAGCACTAAATTCACATT
CNE9_Mouse           279 TTCCGCACACGTAAATGTCGATA-GCGCGCCTTCTCCATCATCAGCACTAAATTCACATT
CNE9_Human           279 TTCCGTGCACGTAAATGTCGCTA-GCGTGCCTTCTCCATCATCAGCACTAAATTCACATT
CNE9_Lamprey          29 TTCTGCACACGTAAACATTGACGGGGGCGTCCTCTCCATCATCCAAAGTAAATTCACTCT

                     361 .......370.......380.......390.......400.......410.......420
CNE9_Anolis          216 GATG-CCTCTT-TTCTTCTCTGTATTGATCCTTCCATGAGAACGTAGATTTGTATCTGTT
CNE9_ElephantShark    81 GATG-CCTCTT-TTCTTCTCTTTATTGATCCCTTCATGAGAACGTAGATTTGTATCTGTT
CNE9_Zebrafish       110 GATG-GCTCTT-TTCTTCTCTTTATCGATCCGGCCATGAGAACGTAGATTTGTATCTGTT
CNE9_Xenopus         198 GATG-CCTCTT-TTCTTCTACGTATTGATCCTTCCATGAGAACGTAGATTTGTATCTGTT
CNE9_Chicken         217 GATG-CCTCTT-TTCTTCTCTGTATTGATCCTTCCATGAGAACGTAGATTTGTATCTGTT
CNE9_Mouse           338 GACG-CCTCTT-TTCTTCTCGGTATTGATCCTTCCATGAGAACGTAGATTTGTATCTGTT
CNE9_Human           338 GATG-CCTCTT-TTCTTCTCCGTATTGATCCTTCCATGAGAACGTAGATTTGTATCTGTT
CNE9_Lamprey          89 GAAGGCCTATTCTTCCCCCCCGTATTGATCTCTTCATGTGAATGTAGATTTGTATCTGTT

                     421 .......430.......440.......450.......460.......470.......480
CNE9_Anolis          274 AATTAATGCGTCCTGAAACAGCGTTTGTATTAATCAGGCAGATAAGAAAAATTGGATGCC
CNE9_ElephantShark   139 AATTAATGCATCCTGAAACAGCGTTTGTATTAATCAGTCAGATAAGAAAAATTGGATGCC
CNE9_Zebrafish       168 AATTAATGCGTCCTGAAACAGCATTTGTATTAATCAGGGAGATAAGAAAAATTGGATGCC
CNE9_Xenopus         256 AATTAATGCGTCCTGAAACAGCGTTTGTATTAATCAGGCAGATAAGAAAAATTGGATGCC
CNE9_Chicken         275 AATTAATGCGTCCTGAAACAGCGTTTGTATTAATCAGGCAGATAAGAAAAATTGGATGCC
CNE9_Mouse           396 AATTAATGCGTCCTGAAACAGCGTTTGTATTAATCAGGCAGATAAGAAAAATTGGATGGC
CNE9_Human           396 AATTAATGTGTCCTGAAACAGCGTTTGTATTAATCAGGCAGATAAGAAAAATTGGATGCC
CNE9_Lamprey         149 AATTAATGCAGTGGGAAGCAGCCCTTGTGTTAATCAGCGAGATAAGAAAAATTGGACACT

                     481 .......490.......500.......510.......520.......530.......540
CNE9_Anolis          334 TGCACCCTCATGACAACCGTAAAAGTGCTGGC--CCTGATAAAAATCATGGACGGACCTC
CNE9_ElephantShark   199 TGCACCCACATGACAACCGTAAAAGTGCTG-C--CCTGATAA-AATCATTGATAGGCCTC
CNE9_Zebrafish       228 TGCACTGGCATGACAACCGTAAAAGCGCGAGCCCCCTGATAA-AATCATTGATGGACCTC
CNE9_Xenopus         316 GGCACCCTCGTGACAGCCACAAAAGTGCTGGT--CCTGATAA-AATCGTGGACGGACCTC
CNE9_Chicken         335 TGCACCCTCATGACAACCGTAAAAGTGCTGGC--CCTGATAA-AATCGTGGATGGACCTC
CNE9_Mouse           456 TGTACCCCTGTGACAACCGTAAAAGTGCTGGC--CCTGATAA-AATCATGGATGGACCTC
CNE9_Human           456 TGCACCCCCCTGACAACCGTAAAAGTGCTGGC--CCTGATAA-AATCGTGGATGGACCTC
CNE9_Lamprey         209 GGGATGGGGCTGACAACAGTAAAAGTGTCAG---GCTGATAA-CATCACCAATATCCCGT

                     541 .......550.......560.......570.......580.......590.......600
CNE9_Anolis          392 AATAA--------------AAAAGTTCCTCCTTCCACTCAATAAACTAATCATCCTGCTT
CNE9_ElephantShark   255 AATAAA-------------AAAAGTTCCTCCTTTCACTCAATAAACTAATCATCCTGCTT
CNE9_Zebrafish       287 AATAAAGAGGGAGAAAAAAAAAAGTTCTTCCTTCCACTCAATAAACTAATCATCCGGCTT
CNE9_Xenopus         373 AATAAAAA----AAAAAAAAAAAGTTCCTCCTTCCACTCAATAAACTAATCATCCCACTT
CNE9_Chicken         392 AATAA--------------AAAAGTTCCTCCTTCCACTCAATAAACTAATCATCCTGCTT
CNE9_Mouse           513 AATAA--------------AAAAGTTCCTCCTTCCACTCAATAAACTAATCATCCTGCTT
CNE9_Human           513 AATAA--------------AAAAGTTCCTCCTTCCACTCAATAAACTAATCATCCTGCTT
CNE9_Lamprey         265 AGATA--------------AAAAGTTCTGCC-AGCGCTCAATAGACCAATCATCCCGGTT

                     601 .......610.......620.......630.......640.......650.......660
CNE9_Anolis          438 TTAATTATTCGGCAGAAAGATGTGTGGAGATTGGAGAAT-GTGTAAATCTA----TTTAC
CNE9_ElephantShark   302 TTAATTATTAGGCCGA-AGATGTGTGTAGATTGG-GAAT-GTGTAAATCTAATTATTTAC
CNE9_Zebrafish       347 TTAATTATTCTGCGGAGAGATGTGTGGAGATTGGGGAAT-GTGTAAATCTAATTATTTAC
CNE9_Xenopus         429 TTAATTATGCCGTAGAAAGATGTGCGGAGATTGGAGAAT-GTGTAAATCTAATTATTTAC
```

```
CNE9_Chicken        438 TTAATTATTCGGCAGAAAGATGTGTGGAGATTGGAGAAT-GTGTAAATCTA----TTTAC
CNE9_Mouse          559 TTAATTATTCGGCAGGAAGATGTGTGGAGATTGGAGGACGGTGTAAATCTA----TTTAC
CNE9_Human          559 TTAATTATTCGGCAGAAAGATGTGTGGAGATTGGAGAAT-GTGTAAATCTA----TTTAC
CNE9_Lamprey        310 TTAATTATTGGCCCGA-AGA----------T-----------------------------

                    661 .......670.......680.......690.......700.......710.......720
CNE9_Anolis         493 TAACAGCAGTGTCTGGGAGGGAGAATAGGG-CTGTCACAGGACAGTGCTGCAGGCGACGT
CNE9_ElephantShark  359 CAACAGCAGTGTCTGGTGGAGGGGG-AGGG-G-G--ATAGAGAGAAAATA-AAACTA---
CNE9_Zebrafish      406 TAACAGCAGTGTCTGAGAGGGAGGGCTGGGAGTGGCACAGGG-GGAGCTCTAGGCGATGG
CNE9_Xenopus        488 TAACAGCAGTGTCTGGGAGGGAGAATTG-G-CTGTCACCGAAAGGTGCTGCGGGCTATAT
CNE9_Chicken        493 TAACAGCAGTGTCTGGGAGGGAGAATAGGG-CTGTCACAGGACGGTGCTGCAGGCTATGG
CNE9_Mouse          615 TAACAGCAGTGTCTGGGAGGGAGGACAGGG-CTGTCTCAGGACGATGCTCCGGGCTATGA
CNE9_Human          614 TAACAGCAGTGTCTGGGAGGGAGAATAGGG-CTGTCACAGGAAGGTGCTGCGGGCTACAC
CNE9_Lamprey        330 ------------------------------------------------------------

                    721 .......730.......740.......750.......760.......770.......780
CNE9_Anolis         552 TCTCTGTCCATCTGCTGTAGCAGAAAAACTGGTTCTTGAGAG-A-GAAA--GGGCAGAGA
CNE9_ElephantShark  410 ------GCTCTCTGCCGTCGCACGAA-GGTG---CT-G----------------------
CNE9_Zebrafish      465 GCTCCGTCCATCTGCCACTCCAC-------------------------------------
CNE9_Xenopus        546 TCTCTGCCCATCTGCTCCTGCAGAACAACAA---C----------AAAA-A---------
CNE9_Chicken        552 TCTCTGTCCATCTGCTGCTGCAGAAAAACTGGTTCTTGAAAG-ACGGGAAGGAACAGAAA
CNE9_Mouse          674 GGTCCCTCCATCTGCTTCGGCA-ACAGACTGCTGTTTGGGATCCTGAAGCGGAAGAATTA
CNE9_Human          673 GCTCTGTCCATCTGCTGCTGCAGAGAAACTGCGACTTGGGAGCATGAAAAGGAACAAGAA
CNE9_Lamprey        330 --TCTGACCA--------------------------------------------------
```

CNE10

```
                      1 ........10........20........30........40........50........60
CNE10_Anolis          1 AATGAGCAGTGCAGCCTGTTCTTTCTGCAGGAT---------------------------
CNE10_ElephantShark   1 CACA--C-A---------------------------------------------------
CNE10_Zebrafish       1 CATG--CCA-----C-----CTTTCATT--GAG---------------------------
CNE10_Xenopus         1 TGCC---A-C--------------------G-G---------------------------
CNE10_Chicken         1 ATGG--CAAA-AAGGATGTTCTTGCAGCAGGAG---------------------------
CNE10_Human           1 TCTC--TAAG--AGGATATTTATTAATT-TAACCCCAAATGCAATGACAAAAGCAGAATA

                     61 ........70........80........90.......100.......110.......120
CNE10_Anolis         34 ----------------------------------CCTTGTCAAAGCACATGTAAGCAGCT
CNE10_ElephantShark   7 ------------------------------------CAGTT-------------------
CNE10_Zebrafish      20 ----------------------------------GCCAGTT--------TGTT-------
CNE10_Xenopus         9 ----------------------------------CCAAGTCA------ATGT--------
CNE10_Chicken        31 ----------------------------------CCCTGTCAGAGAACGTGTAAGCAGCT
CNE10_Human          56 AGAAACAAGACTTACACAGCCAGTCCAAAAGAAATCCTGTCAAATAACATGTAAGCACCA

                    121 .......130.......140.......150.......160.......170.......180
CNE10_Anolis         60 CCATAAACAACTGAATGAAAGC--TTTAGACATGTCTTTAAGACCCCCTTAAGAAAAGCT
CNE10_ElephantShark  12 -------------------------------------------------T--G-AAAGCT
CNE10_Zebrafish      31 -----------TGCAAGAATGC--CGCAGTCTTGCATT--AGACCCCCTTAAGAAATTCT
CNE10_Xenopus        21 -----------------AAAGC--CAC-------------AGACCCCCTTAAGAAAAGCT
CNE10_Chicken        57 CCATAAACAACTAAATGAAAGC--CTCAGACATGCCTT-CAGA-CCCTTTAAGAAAGGCT
CNE10_Human         116 TGATAAACAACGAAAGAAATGCATTTTACACATATCTTAAAGATTCCCTTAAGAAAAGCT

                    181 .......190.......200.......210.......220.......230.......240
CNE10_Anolis        118 TGAC-TAATCCCCTTTGATGATTTTATTAAGTACTTGGTGGTTGCTGGTGCCAACTGGCA
CNE10_ElephantShark  20 TGGC-TAATCCCCTTTGATGATTGTATTAAGTGCTTAA-GGCGAGCTCTGGCAACTGGCA
CNE10_Zebrafish      76 TGGCTTAATCCCTTTTGATGATTTTATTAAGTAGTAGA-AGATGGCCCTGCCAACTGGCA
CNE10_Xenopus        49 GCAC-TAATCCCCTTTGATGATTTTATTATGT----------------------------
CNE10_Chicken       113 TGAC-TAATCCCCTTTGATGATTTTATTAAGTAGTCGT-GGTTGCTGGTGCCAACTGGCA
CNE10_Human         176 TGAC-TCATCTCTTCTGATGATTTTATTAAGCACTTAT-AATTAATGGTACCAAATGGCA

                    241 .......250.......260.......270.......280.......290.......300
CNE10_Anolis        177 AACCACTATGTTTT--ACCAACAGGCCAGGAAATCACT--CCTTC--AC-----------
CNE10_ElephantShark  78 TTCCACTCTGCGTCAAACCGACAGCCTTGTTTTTT-CA--AAAA----A-----------
CNE10_Zebrafish     135 TTGGGCACACTTGC--ACCAGCAGGCCAGGAGAGG-CA--CCAA----C-----------
CNE10_Xenopus        80 ------------------------------------------------------------
CNE10_Chicken       171 AACTCATGCAGCTT--ACCAACAGGCCAGGAAATCACA--TCCTCTCCCCTCTTGTTCCA
CNE10_Human         234 AACCACTGTTTTTC--ACCAATAGGTCAGGAAATC-CTTTCCTT----T-----------

                    301 .......310.......320.......330.......340.......350.......360
CNE10_Anolis        220 TGGCGCCTTATGCGAGGGTGGATAGTAACAGTCAGAGATAATAAATTAACAGCTGCTGTA
CNE10_ElephantShark 120 A----TCTCA--------------------------------------AAA---CCTGTA
CNE10_Zebrafish     175 T----GCTCA--------------------------------------CCA---GCTCTT
CNE10_Xenopus        80 ------------------------------------------------------------
CNE10_Chicken       227 TAGCTCCTAATGTGATTG-GGATATTAGCAGTTTGATATCAGAAATTAACAGGTACTGTA
CNE10_Human         276 T----CTTTA---------------------------------------CTGT-GCTG-T
```

EL161

```
                        361 .......370.......380.......390.......400.......410.......420
el161_Anolis            347 TTAAGAAATCAAGGCAGGA-TTC-T--TCTCTGCATTTGAACTTAAAAGTGCTGAAACAG
el161_ElephantShark      12 -------------------------------------------GAATCA-----------
el161_Zebrafish          10 ------------------------------------------------------------
el161_Xenopus            44 --------------C-A--------------------------TAAATGTGCTGAAGCAG
el161_Chicken           309 TTAAAA-ATCTCAGAAAGA-TTTTTTTTTCCTGCATTTGAATTTAAAAGTGCTGAAGCAG
el161_Mouse              29 -------------GACAGGAGTG----------------GGCTAAAATAGG-------GG
el161_Human              83 TTAAGA-ATTATGGCAATAACTG-G--TCTTTACATTCGAACTGAAACATGCTAAAATAG
el161_Lamprey             8 ------------------------------------------------------------

                        421 .......430.......440.......450.......460.......470.......480
el161_Anolis            403 TGAACCACTGTATGATAAGTAAAAAATCGTTTGTTCTTTGTTGTTT-TTTCTTTTGTGCT
el161_ElephantShark      18 ---------------TGAAT-A-AAATCGTTTGTGCTTTGTTGTTT-TTTCCCT----TC
el161_Zebrafish          10 ------------------------A---GTCTCTGCT-----------------------
el161_Xenopus            63 TGAC-CGGGATCAGATAAGTAA-AAATCGTTTGGGCTTTGTT-----TTCCCTTTGTGTC
el161_Chicken           367 TGAA-TACTGCCAGATAAGTAA-AAATCGTTTTTGCTTTGTTGTTT-TTCCTTTTGTGTC
el161_Mouse              53 CATA-C----TCTGGTAATTCA-AAATGGTTGGTATTTTGTTATATTTCCCCTTTGTGCC
el161_Human             139 CAAC-C----TCTAATAAGTAA-AAATCGTTGGTGCTTTGTTGTTT-TTCCCTTTGTGCC
el161_Lamprey             8 ------------------------------------------------------------

                        481 .......490.......500.......510.......520.......530.......540
el161_Anolis            462 TC-CCTAATGCTTT-ATTGTGGT--CTTAAGTCCCTTTTAGCATTTGCATAAAGTTCATG
el161_ElephantShark      56 TC-CCCAATGCTTT-ATCGCAGT--CTTTAGACACT-TTAGCATTTGCATAAAGTTCATG
el161_Zebrafish          20 ----CAGATGCC----------T--CTTAAGT----------------------------
el161_Xenopus           116 AC-CTTAATGCTTTTCCTGTGGT--CTTAAGTCCCTTTTAGCATTTGCATATAGTTGATG
el161_Chicken           424 TC-CTTAATGCTTT-ATTGTGGT--CTTAAGTCCCTTTTAGCATTTGCATAAAGTTCACG
el161_Mouse             107 TTCTCTAAGGCTAG-ATTGTAGT--ATTAAGTCTCTTTTAACATTTGCATAAAGTTCAGG
el161_Human             192 TCTCTTAATGCTTT-ATTATGGT--CTTAAGTCTCTTTTAGCATTTGCATAAAGTTCAGG
el161_Lamprey             8 --------GGCCA--------GCGTCTT--------------------------------

                        541 .......550.......560.......570.......580.......590.......600
el161_Anolis            518 ATAGATCCCCCTTTAATGACGTGCCGATCATCAGATACCTGTGTGTCAATAACATGCTCT
el161_ElephantShark     111 ATAGATCCCTCTTTAATAACATACTGATCGTTAGCTACCTGTGTGTCAATAACA-GCTCT
el161_Zebrafish          36 -----------------AAGGAGCCCATCACTATGCACCTGCATGTCAATAACAGGCTCT
el161_Xenopus           173 ATAGATCAGTGCTTAATAACATGCTGATCATTAGATACCTGTATGTCAATAACACGCTCC
el161_Chicken           480 ATAGATCCCTCTTTAATGACGTGCCGATCATTAGATACCTGTGTGTCAATAACATGCTCT
el161_Mouse             164 ATAGATCCCTCC-TAATGACGAGCTGATCATCAGATACCTGCGTGTCAATAACG-GCTCT
el161_Human             249 ATAGATCCCTCCTTAATGACGTGCCGATCATTAGATACCTGCGTGTCAATAACGTGCTCT
el161_Lamprey            20 ------------------------------------------------------G-GTCT

                        601 .......610.......620.......630.......640.......650.......660
el161_Anolis            578 GATGCTATGTACCATTGACAGTCACACCGTGCCC-CTCCATC----------TGCTTTTG
el161_ElephantShark     170 GATGTTGTGTACCGTTGACAATCACGCCATGTCC-CTCCATTTCCCCCTGCCTCCTTTTG
el161_Zebrafish          79 GATGTTGTCCACCATCGACAGTCACACCATGGCC-CTCTGGC----------TCCCTTTG
el161_Xenopus           233 CATGCTATGTGCCATTGACAGTCACACCGTGCCC-CTCCATC----------TGCTTTTG
el161_Chicken           540 GATGCTATGTTCCATTGACAGTCACACCGTGCCC-CTCCATC----------TGCTTTTG
el161_Mouse             222 GAAACCATGTACCATTGACAGTCACACGACACTCGTTCCAGC----------TGCTTTTG
el161_Human             309 GATGCTATGTACCACTGACAGTCACACCGTGCCC-CTCCATC----------TGCTTTTG
el161_Lamprey            25 GAGGC-------C--------------------------AGC----------T-------

                        661 .......670.......680.......690.......700.......710.......720
el161_Anolis            627 TTTTGACCCTATCTTTGAACTTTATCTTGGTTGCTGGCCAGTAGTTTTCCCTTTAATTAC
el161_ElephantShark     229 TTTTGACCTAATCTTTGAACTTTATCTTGGTTGCCGGCCAGTAGTTTTCTCTTTAATTAC
el161_Zebrafish         128 TGTTGACCCCATCTTTGAACTTTATCTTGGTTGCTGGCCAGTAGTTTTCCCTTTAATTAC
el161_Xenopus           282 TTTTGACCCTTTCTTTGAACTTTATCTTGGTGGCTGGCCAGTCATTTTCCCTTTAATTAC
el161_Chicken           589 TTTTGACCCTATCTTTGAACTTTATCTTGGTTGCTGGCCAGTAGTTTTCCCTTTAATTAC
el161_Mouse             272 TTTTGACCCTATCTTTGAACTTTATCTTGGTTGCCGGCCAGTAGTTTTCCCCTTAATTAC
el161_Human             358 TTTTGACCCTATCTTTGAACTTTATCTTGGTTGCCGGCCAGTAGTTTTCCCTTTAATTAC
el161_Lamprey            35 -----------------------------------GGC------TTTTCTCGTTAATTAC

                        721 .......730.......740.......750.......760.......770.......780
el161_Anolis            687 AA-GAAGGAAACTGTCAATAAAATCTGTTAAATGGGATGCAATGAGTGGCTTGAATGGGC
el161_ElephantShark     289 AA-GAAGGAAAC--TCAATAAAATCTGTTAAATGGAATGCAATGAGTAGC-TGAATGGGA
el161_Zebrafish         188 AA-GAAGGAAACTGTCAATAAAATCTGTTAAATGGGACGCAATGAGCAGCTTGAATGGGA
el161_Xenopus           342 AA-GAAGGAAACTGTCAATAAAATCTGTTAAATGGGATGCAATGAGTGGCTTGAATGGGC
el161_Chicken           649 AA-GAAGGAAACTGTCAATAAAATCTGTTAAATGGGATGCAATGAGTGGCTTGAATGGGC
el161_Mouse             332 AA-GAAGGAAACTGTCAATAAAATCTGTTAAATGGGATGCAATGAGTGGCTTGAATGGGC
el161_Human             418 AA-GAAGGAAACTGTCAATAAAATCTGTTAAATGGGATGCAATGAGTGGCTTGAATGGGC
el161_Lamprey            54 AACGGGGGAAAATGTCAATAAAATGTGTTAAATGAAATGTAATGAGTGGC-CGTATGGGA

                        781 .......790.......800.......810.......820.......830.......840
el161_Anolis            746 GGACGGCTATTTGTTCAAATTCTGCAGCATTCAAGGTATTGACAGTTTGTTTTCTGGGGC
el161_ElephantShark     345 GGACAGCCATTTGTTCAAATTCTGCAGCACTCAAGGTATTGACAGTTTGTTTTCTGAGGC
el161_Zebrafish         247 GGGCGGCTATTTGTTCAAATTCTCCAGCGCTCAAGGTATTGACAGTTTGTTTTCTGGGGC
el161_Xenopus           401 AGACGGCTATTTGTTCAAATTCTGGAGCATTCAAGGTATTGACAGTTTGTTTTCTGGGGC
```

```
el161_Chicken        708 GGACGGCTATTTGTTCAAATTCTGCAGCATTCAAGGTATTGACAGTTTGTTTTCTGGGGC
el161_Mouse          391 GGATGGCTATTTGTTCAAATTCTGCAGTATTCAAGGTATTGACAGTTTGTTTTCTGGGGC
el161_Human          477 GGACGGCTATTTGTTCAAATTCTGCAGCATTCAAGGTATTGACAGTTTGTTTTCTGGGGC
el161_Lamprey        113 GGGCAGTCATTTGTTCGCATTGTTCGCTCTTCAAGGTATTGACAGTTTGTTTT--GGGAG

                     841 .......850.......860.......870.......880.......890.......900
el161_Anolis         806 CATATGTGACGATCAATCTCAGGCTGG-GCTCAGCCGCGCTGAAAAATGAAAAACCAGAT
el161_ElephantShark  405 CATATGTGTTGATCAATCTCAGGCTGG-GCTCAGCCACGCTGAAAAATGAAAAACCAGAT
el161_Zebrafish      307 CATATGTGACGATCAATCTCCGACTGG-GCCTGGCCATGCTGAAAAATGAAAAAGCAGAT
el161_Xenopus        461 CATATGTGGCGATCAATCTCAGGCTGG-GCTCAGCCGCGCTGAAAAATGAAAAACCAGAT
el161_Chicken        768 CATATGTGACGATCAATCTCAGGCTGG-GCTCAGCCGCGCTGAAAAATGAAAAACCAGAT
el161_Mouse          451 CATATGTGACGATCAATCTCAGGCTGG-GCTCAGCCGTGCTGAAAAATGAAAAACCAGAT
el161_Human          537 CATATGTGACGATCAATCTCAGGCTGG-GCTCAGCCGCGCTGAAAAATGAAAAACCAGAT
el161_Lamprey        171 CA-GCGCGTTGATCAATGGT-GGCTGCGAATCGGTCAAACTGAAAAATGAAAAAGCAGAT

                     901 .......910.......920.......930.......940.......950.......960
el161_Anolis         865 TGCTTTTGCTTACTTGTGGATGACGACTT-GTGATTTGGTACGGTCCTAGTGAGAT---G
el161_ElephantShark  464 TGCTTTTGCTTATTTTCGGATGACAGCTC-TTGATTTGGTG-GGTCC----GAGAT---G
el161_Zebrafish      366 TGCTCTTGCTCGCTTGTGGATGACGGCTAGCTGCATTGGCCCTAGCCTGGAGACACTGGG
el161_Xenopus        520 TGCTTTTGCCGACTTGTGGATGACGACTT-GTGATTTGGTGCAACCCTACAGAGCG---G
el161_Chicken        827 TGCTTTTGCTTACTTGTGGATGACGACTT-GTGATTTGGCGCGGTCCTAGTGAGAT---G
el161_Mouse          510 TGCTTTTGCTTACTTGTGGATGACGACTTTGTGATTTGGCGCGGTCTGGGCAAGAT---G
el161_Human          596 TGCTTTTGCTTACTTGTGGATGACGACTTTGTGATTTGGCGCGGTCCTAGTGAGAT---G
el161_Lamprey        229 TGGCTTTGCTCAGTTTCAGCGGAGGCATTAGAGGC----TGCGGTAAGCCACAGAC---G

                     961 .......970.......980.......990......1000......1010......1020
el161_Anolis         921 AGACCTTCTGCCCAAATTGCCCCCCCGAGAG--CCAG--GGTGCGTGACTGTTTTTAGAA
el161_ElephantShark  515 A-ACCTTCTGC-CAAATTGTACCCCTGAGAA--TCA----AAGCGTGACTGTTTTGAAAA
el161_Zebrafish      426 GGCCGTTCTGCCCAAAGTGCACCCCTGAGAGAACGAGCAAGCGCGTGACTCTTTTGATAA
el161_Xenopus        576 AGACCTTCTGCCCAAATTGCCCCCCAGAGAG--ATGG-AAATGTGTGACGGTTTATAGAA
el161_Chicken        883 AGACCTTCTGCCCAAATTGCCCCCCTGAGAG--CCAG--GACGCGTGACTGTTTTTAGAA
el161_Mouse          567 AGACCTTCTGCCCAAATTGCCCCCATGAGAG--CCAG-GGATGCGTGACTGTTTTTAGAA
el161_Human          653 AGACCTTCTGCCCAAATTGCCCCCATGAGAG--CCAG-GGACGCGTGACTGTTTTTAGAA
el161_Lamprey        282 AGG-CTTTTGTC-TAATCGTGCCCCCGAGAA--CTA----AAACGTGACTCCTTTC-GAA

                    1021 ......1030......1040......1050......1060......1070......1080
el161_Anolis         977 ATAAT--TTTTAATTGATTTTGTAATTAACA-CTTCATCTACAATATTGATGCACAGATC
el161_ElephantShark  567 TTATT--TTTTAATTGATTTTGTAATTAACA-GTTCATCTCGTATATTGATGTGCAAATT
el161_Zebrafish      486 ATTCT-TTTTTAATTGATTGCACTATTAGCA-GTTCATCTCA--TCTTGT----------
el161_Xenopus        633 ATATTGTTTTTAATTGATTTTGTGATTAACA-GTTCATCTATAGCATTGT----------
el161_Chicken        939 ATAAT-TTTTTAATTGATTTTGTAATTAACA-GTTCATCTACAATATTGATGCATGAATC
el161_Mouse          624 ATAAT--TTTTAATTGATTTTGTAATTAACA-GTTCATCTAAAATATTGATGCATGAATC
el161_Human          710 ATAAT--TTTTAATTGATTTTGTAATTAACA-GTTCATCTACAATATTGATGCATGAATC
el161_Lamprey        333 ATTAT--TTTTAATTGATTTTGTAATTAACGGGTTCAGCCCTTCCATCGACGTAGGG-CC

                    1081 ......1090......1100......1110......1120......1130......1140
el161_Anolis        1034 TTCAGACGGATAGGAGGAAAATTGTTTTCAACAATGAAGTTGTACTTTCCT-ATTTGTCT
el161_ElephantShark  624 CCTAGATTGATGGGAGAGAAATTGTT-TCAACAAA-AAGTTGCACTTTTTTCATTTGTCT
el161_Zebrafish      532 -------TGACG-----------------------------CACA----------CGTC-
el161_Xenopus        682 -------CGATG---------------------------------------------GTCT
el161_Chicken        997 CTCAGATTGATAGGAGGGAAATTGTTTTCAACAATGAAGTTGTACTTTCCT-ATTTGTCT
el161_Mouse          681 CTTGGACTGATGGAAGGGAAATTGTTTTCAACAATGAAGTTGTACCTTCCT-ATTTGTCT
el161_Human          767 CTTAGACTGATAGGAGGGAAATTGTTTTCAACAATGGAGTTGTACTTTCCT-ATTTGTCT
el161_Lamprey        390 TGTTGACTGATGGGAAGGAAATTGTT-TCACCAA---AGTAACACCGTGCTCATTTGTCT

                    1141 ......1150......1160......1170......1180......1190......1200
el161_Anolis        1093 TCATGATGGGATCTAGCAGTTTGAATATTTAATTGGG-ATTTAATTTTTT-TTTTA----
el161_ElephantShark  682 C-------------AGC-------------------------------------------
el161_Zebrafish      545 --------------GGC-------------------------------------------
el161_Xenopus        691 TT--GATAGAAT----CA-------GTTT----------------TT--------TA----
el161_Chicken       1056 TTGTAATGTGATTTAGCACTCCGCATTTTTAATTGGAAATATAATT--------CAA--A
el161_Mouse          740 TTATAATGGGTGTTAGCATTTCACAGTTTTAAATGTGGATATAACCCACTT-ATTACCAC
el161_Human          826 TCATAATGCAGTTTAGCATTTCACAGTTTTAATTGTGAATATAACCCTCTTCCTCACCAC
el161_Lamprey        446 TCT-------------C-------------------------------------------

                    1201 ......1210......1220......1230......1240......1250......1260
el161_Anolis        1147 ----------------------A-T----TAGGAAGC---AAAA--C------C----A-
el161_ElephantShark  686 ----------------------------------------A-AC--T-G----C---T--
el161_Zebrafish      548 ------------------------G----TG-----CC--A-A-----------------
el161_Xenopus        711 -------------------------------C----------AG--T-G----C------
el161_Chicken       1106 A---------------------ACA----TGCAAAGC--CAAAA--T-G----CTTGTT-
el161_Mouse          799 ACAATTCACCCTACTTTCCCTCACTTTCCTGGGAACTT--AAATTG-----------TG-
el161_Human          886 ACCATGCACCTCTCTTCTTCT-ACGCTCCTGTGAAATTATAAAATAT-GATTTCTTATGA
el161_Lamprey        450 ----------------------------------------A-AG--TAG----C------
```

4. ERR CNE sequences in FastA format

CNE1

```
> CNE1_Anolis
TACAGTAGTTCGTTACCACTGCAGCTTATCTTCTGAGACATGTTTTGCTTTATAAAGGCTTTCCTTAACT
CAGGCCTTGTCTCTACCAGTAGAGCCGAACAATATAAAGTGCAATTTTGAATCATTCTAATTACTTCTCC
AAACCAAATAATAAGCAACAATCAATATGCATCTAAATCATAATTTGCTTAGAAAGGAGGACAGCAACAC
ACATTCTGAATTGCTCCATATTTTCCCTACAGAAGAAATTTAGTTGTTCAGAGTTTGCATCATAAATGGC
TTATAAACGAGCTTTTGAATCATGAGCAATGTTTTTCTGGTACAGTTAACTTATTTCTGTCTCCTTAAAC
TAGTTTGTCTTTATGCTTTGAATGCTACAGTAATTTCTTTAATCAAATTGATGGAAGTCCCTTTCCCAGC
CCACACTGAAACCAATTTATTGGTCATGATACGCCAGCTATGTTACTAACAGTCGTAACGAACTGTCAGG
CAATATCGCAGTGAAGCTAATTTGAAGTCATTAGGTGTGCAGCGTTCAGGCAACAATTTGTTACAGTGGT
TAGGAACACAGCTGACATTTCTGAATGGCTTTGTTCAGAGCAGGTCTAGATGAGCCCAAGCAAATTGTAA
ACTAATTTAAACATCACTCTGTATTATACTAGCTACTTTGTCTTACACTGGTTGCTAATAAAGCACCCCA
GATTAAGTCAAAGCTAGTTAAATAGGTGAGTTTGAGTTTCATCTCTAGACAATGCCTACATCAGTCTTAC
CAGTATGGGGGCATTCGTCCTAGAAGTCAGTCAATAACACATCATAGCGATTGCCAAATTTCTTTTATCA
GTTTGATTTGTGCTGTGAGTAGAACGCGTGGCATAGACTTTCTTATGGTTGTGGAATAAAGCAAGAAATT
GAGAGGAGAGAGGGAAGGAGACAATGAACACAAATAATGGTTCAGTGCCTGGTGTTAGCAATGAAAGCTG
CTGGGTGCTTTAGACTGGCATTCTAAATTACAGACCAGGCATTTGAACCAGTTGT
> CNE1_Zebrafish
CTGTTAACCTCCTCTAACCGCGCATTTGACTCTTTTCTGACTAGTTTGTCTCTGCGGTTTCTATGGTACA
GTAATTTTTTTTAATCAAATTGATGAAGGCCTCCATTCTCCCCGAGAGCCCAAACCAATTTATGGCTCAT
GATACGGCGGCTATGTTGCTAACAGACGTAACAAACTGTCAGCCTCTCGCACAGAAGCTAATTTGAAGTC
ATTAGGTGTGCAGCGTACGGGCAACAATTTGTTACGGTGGTTAGGAGCGCCGCTGACATTTCTGAGCGCC
TTCGCTTACGATCTGTCCTAGGCGAGGAGCAGGCAAATTGTAAACTAATTTAAACATCACTCAGTATTAC
TGGAGCTGCCGCTGCGGAGAGCTGGCGG
> CNE1_Xenopus
ATTCAGATGTTAGGAGTGCATTTGATAGTAAAATCAATGTGTTTCAGCTGTGGTTAACTTCTGTCTTTTT
TCCCTAAACTACTTTGTCTTTATGCTTTGAATGCTACAATAATTTCTTTAATCAAATTGATGGAGGTCCC
TTTCCCAGCCCCAACTGAAACCAATTTATTGGTCATGATACGCCGGCTATGTTGCTAAGAGTCGTAACGA
ACTGTCAGGCAATATCGCAGTGGAGCTAATTTGAAGTCATTAGGTGTGCAGCGTTCAGACAACAATTTGT
TACAGTGGTTAGGAGCGCAGCTGACATTTCTGAATGGCTTTGTTCAGAGCTAGAACTATATGAGCCCAAG
CAAATTGTAAACTAATTTAAACATCACTCTGTATTATACCACCCCGGCTACTTTTTTTTCCACCGACGCC
GCTTTCTAATAAAGCGCCCCGAATTAAGTCAAAGCTGGTTAAATAGGTGAGTGTATTATTTTATTCTGCT
TTTATCTTTGCAAGGCAGCTCCGCCGGGCAAATTTAAATGGAACAATAATGTTAGA
> CNE1_Chicken
ATGTTAACCAGTCAAGGTCTCAGATGTTCTGGTAGGTGCTTCTAGGTAATCTCTGGTAATAAGAGACACT
AACAAATAGTAATAATAAAATAATGTGTGTGTTTGTGGAAACTTATTTTATTTTAGATGAAAGATAACAG
CGTACGGATTGTAAAATAGTCTGTAGGCCACACTGTTGAGATTTAGTCATTCTCTGCTATAAATGGTGTA
TAAACAAGCTTTTGTATCACAAGCAGTGTTTTTCTGGTACAGTTAACTTATTTCTGTCTCCTTAAACTAG
TTTGTCTTTATGCTTTGAATGCTACAGTAATTTCTTTAATCAAATTGATGGAAGTCCCTTTCCCAGCCCA
CAGTGAAACCAATTTATTGGTCATGATACGCCAGCTATGTTGCTAACAGTCGTAACGAACTGTCAGGCAA
TATCGCAGTGAAGCTAATTTGAAGTCATTAGGTGTGCAGCGTTCAGGCAACAATTTGTTACAGTGGTTAG
GAACACAGCTGACATTTCTGAATGGCTTTGTTCAGAGCAGTTCTAGATGAGCCCAAGCAAATTGTAAACT
AATTTAAACATCACTCTATATTATACTAGCTACTTTGTCTTACACTGGTTGCTAACAAAGCACCCCAAAT
TAAGTCAAAATGAGTTAAACAGGTGAGTTTGAGCTTCATCTTTGGAACATACCCACATCACCTTAGCCAA
AATAGAATCATTCTCAAAGAAGTCAGTTACGGTCCATAGTAGTTGCAAGGAAACTTTTGTCAGCTTGATT
TGTGAACATGTTGCATAGCTTTAGTTTGGCTGGAGGGGTAGGAAGTGCAGAGAGATGAGTCTG
> CNE1_Mouse
TTTCTTTCTTAATCCATCCTATAACAGCAAAAACATATACCAGGCCATATCACCAAAGGAAACATTTTGA
AAATGTTCGCAATGTGTTTGAATTCATTTTCATTTCCTTTAGCTTTAGTTTTCAAATAGTTTATAAAGAA
GTTTCCAAGTGACAAGAGATGTTTTTCTGGCACACACAGTTAGCTTATTTCTGTCTCCTTAAACTAGTTT
GTCTTTATGCTTTGAATGCAACAGTAATTTCTTTAATCAAATTGATGGAAGTCCCTTTCCCAGCCCACAC
TGAAACCAATTTATTGGTCATGATACGCCAGCCATGTCGCTAACAGTCGTAACGAACTGTCAGGCGGTAT
CGCAGTGAAGCTAATTTGAAGTCATTAGGTGTGCAGCGTTCAGGCAACAATTTGTTACAGTGGTTAGGGA
CACAGCTGACATTTCTGAATAGCTTTGTTCAGGGCAGTTCTAGATGAGACCAAGCAAATTGTAAACTAAT
TTAAACATCGCTCTGTATTATACCAGCTACTTTGTCTTACACTGTGTGCTAATAAAGCTCTCCAAATTAA
GTCAGAACTGGTTAAATAGGTAACTCGAATGTATGCTTTGGCAAGCACCCACACAAGAATAACAATATCT
AGAAGAATACAGTTTGGCCCTGTGATATATGTGGATGGGTCAACTTAGATTATAATCACACTTACATGGA
TTTTATAAAAAAGGCATACAAAGGAAGGATTTAAAAGAACCAGATGGCTAGGTTTGATGATCCTAGCGTT
TGTGAGGCTGAGGCAGGAAGATG
> CNE1_Human
CATCCTGTAATATGAACAAATATGTACATGCTTGTAAGTTTTGAGAATTTTAGTATTCTACTTGAATTAT
TTTTTATTTGTTTGGAGTTGAATTTTCAGATAGTTTATAAACAAGTTTTTGAATGATGAGCAATGTTTTT
CTGGTACAGTTAACTTATTTCTGTCTCCTTAAACTAGTTTGTCTTTATGCTTTGAATGCTACAGTAATTT
CTTTAATCAAATTGATGGAAGTCCCTTTCCCAGCCCACACTGAAACCAATTTATTGGTCATGATACGCCA
GCTATGTTGCTAACAGTCGTAACGAACTGTCAGGCGATATCGCAGTGAAGCTAATTTGAAGTCATTAGGT
GTGCAGCGTTCAGGCGACAATTTGTTACAGTGGTTAGGAACACAGCTGACATTTCTGAATGGCTTTGTTC
GTTGTAGTTCTAGATGAGGCCAAGCAAATTGTAAACTAATTTAAACATCACTCTGTATTATACCAGCTAC
TTTGTCTTACACTGTTTGCTAATAAAGCCCCCCAAATTAAGTCAAAACTAGTTAAATAGGTGAGCTTGTA
TATTCTTTAGAAGACACCTACACAAGGATTACTACATATAAAAGAAGAATAAGAATTTGATTGTGTGGAT
ATTTGATATTGCAGTTGGATTGTAATCACATCTGTATGACTTTTAGAAAAATAAAAAGAAAGGATTTACT
GCAAGATGTA
```

CNE2

> CNE2_Anolis
TTCACTCTTCAATTATTTTCTATTTGGGTACACTCTTAAATTCCGTGCTACTGGAACTGATCAAAACAGT
TCTACAGAAATGCATACACTACTAGTACTAATACATACATGGGGAGCCACATACACATACTCATATATGG
ATAGAAAGGGGTTATCAGCATATAGAATTAGCTTACTTGCATTAATAAATCAAACAAATGAAAAGTATTG
TTGTGATACATGACCACATCTTATCTGGCGATGAAAATGATCAGAGTGGCATTAGGAAATTCTTGACTTT
AGTTACAGGAGCGTAAAGGCACAAGAACAGATGATGGTGCCCTAATAGCCCTTTTTAAGAATGACAGGGC
CCCACCATTAGCATCCTTTTATATCCTTGGAAATTGCTCATGCCTCGCTTTTCTGGTTCCTATCTGCCTG
GCTCTTTTTTATTTCTCTTTCAATTTCGTCCGCGCTCGGATAAGCGCATGCTAACACAATATGATTGAGC
TGTAAAATGGAACGCTGTCGCCTCTAATCTCTTTTATGTAGATATGGAGGTTCTTCCACACTCCACTTCA
TTCTTTCTCCATTGTTGGCCTTTATAGAATGGGTTGCAGAGTAATGGAAGCTCACACTGTCAAATATTAG
AGCTAGGTTTCACAGGGTGTGATAAGAAGATTATCAGCCCAGCAGTCAGATCTATTGTTGTTCTTTGTGT
CAGATTAAAATATTCTTCTGTACCGTAACTTCGTTAGCAAAATACTACAGGTATTTAAGAAGGCGCTAAT
GCTGATTTGTTTTTCACTGAGATAATACCTGCATTAGCTGACCTAGATAAAGTGGTGAGTTAGATTTACA
GAACCTAACTTGGATTCTGAAAAGCATTTGGTGGTTTCATTGTGCACTGCACTTGGAGAAGAGTCAGTTT
TGCTTTTGAACATCTTAAAAGAATATAGGCTATTTTTTCCATGAGATGAGATTCTGCTGATGTCTGACCA
CATTTTCAAAGTCACTTAGGCATTGTTTCTGTAATGCAACTGTCTCATTGGCATTCTACAAAAATGTGCA
ATGCTCTCCTTTTACTAAGTGGAAGCAGGAAACATTAAGCTCTTTAAATGTGCATAACAGGAAGCTTAAG
AGACAAAATGTTCTCTTGTCATTTTGTTTTGCAGACACAAAAGCCCATCAACTAGCCACTGCAACTTTTT
ACACTTAAGATTAGTTTTAATTGGTATGAAAACATTATATCTAGGTTTTTCTGAATTTTTCTCCTCCCCA
AATGTTTTATTTATACATTGTAAATAAACTAAATTGTTTTCAAAGGCTTCCAGGCATGCCTTATTATTTA
TCTTAATATTGCTTATTGGTAGATTTAGGATACCTTTGATTTCAACCAGTTAAGCAAGGTGAAGACTTGC
AGTTATT
> CNE2_ElephantShark
AAATTGCTTTGATTAATTCCTCGTCGCAAAGCACTCAACCCGGCTGTGTCCACAACAGAGGCAAATATTT
AAAGAAAAAATAAAATTACTTTATCAGTAAATAGAATTAGGCTTGCCTCGCTAATAAAGCGGCTGAGCAA
AAAGCAGGCGTTATGAAATTTGAACAGTCCTTATCTGACAAAGAGCACAATGGAATTAGACAGCACTTGA
CTTCAGTCATTTCAAGTTAGAGCAGAATGAGTGGGTACAAGAGATGGTGGCTATGCCCTAATAGCCCTTT
AGCGTGTCAGGCCCCCCCGCCATTAGCATCCTTTTATATTCTTCAAAATTGCCCATGCTGCCTATTCTGG
CTCCTATCTGCCTGCCTGCCCTCTTTTTTTTCTCTCTCCCTTTCAATTCTGTACGTGCTTGGATAAGCGC
GTGCTAACACAATATGATTGAGCTGTAAAATGGAATCCTGTCGGCTTTAATCTCTTTTATGTAGATACTG
TAGTACTTCCACACTCTGCTTCATCCAGTCTATTGTAAGATGCACAGAATGGGCCAGAGAGTAATGTAGG
CTGAATCAAATATTGAAGCTAGCTTTCACGCATACGATAAAAAGATTATCAGTGCAGCAGTCAGACGTAT
TGTTGCACTTTGTGTTGGATTAGAATATCCACATGTAACCTCGTTAACAAAATGTTGCAGGTGCTTACTC
AGGCCCTGCTGCTGCAGTTCTGGGTGCTCCATCTTCATGTTGAAAGAGCTTGTATCTCTTCTTTTGCCCC
TATGTGTCACCGGCATTTAACAACTTGGA
> CNE2_Zebrafish
CACGGACCGGCACTGGCTTTTTAGCATGACAGGACCCTCACTATTACAGGCCCTTTATTCAGGCTGAAAT
TGCTAGGGCTTCTCCTATTCTTGGTCCTATCTCAGAGACTTTCTCTCTTTCTTTCTTTTATTTCCCTTTC
AATTTTCCCTGGCACGCGGATAAGCGCGTGCTAACACAATATGATTGAGCTGTAAAATGGAACCCTGTCG
ACTCTAATCTCTTTTATGTAGATACTGAGGTACTGCCACACTCCACTTCATTCCTCTGCCTCCCTCCCAC
CTCCATCTCTCC
> CNE2_Xenopus
GTGTATGCCAAGGGGCCGGTGCGTTACAGTTTCTTTTGTCTTTTTCCAATCCAAAGGGAGAGCAAAATAC
ATATCTAGTGGCCTTTATCAGCAAATAGAATTAGCCTAGTAAGATTTATAAATCAAATAAATGGAAGGTA
TTTTGTTGTGAGGCATGATGGCGGCTTATCTAGGGGATGGGGACCGATCACAATGGCATTAGGGAAATGC
TTGACTTTAGTTAGAGAAGCGTAAGGGCATAAGAACAGATGATGTTGGCCCTAATAGCCCTTCCTAAGAA
TGACAGCACCCCGGCCATTAGCATTCTTTTCTACGCCTGGAAATTGCTCATGCCTCGCTTTCCTGCTCCC
TATCTGCCCGAGTCTTTTTTATTTCTCTTTCAATTTTGTCCGCGCTCGGATAAGCGCACGCTAACACAAT
ATGATTGAGCTGTAAAATGGAACGCTGTCGCCTCTAATCTCTTTTATGTAGATATGGCGGCCCCCTTCCA
CACTCCCTGCCCATTGTGGTCTCGCCTTAGCGAGAAGGGACTTTAGGGTATTGGCGGCAGTGTCAGAGGA
TGGGATAAGGAGATTACCAGCCAGGGAACATATCTATTGCTCCT
> CNE2_Chicken
TATACAAAGTTTGAATGCTTTCATAAAACCATTAGTATTTTTGTGCTATTGGAAACATTTTCAATAGTTG
CTTTTATTGAATAAAAAGTCTATGCCAACAACAAAAGGAAATATTGAAAGAAAAATATATATGTAAGGCC
TTTATCAGTAAATAGAATTAGTCGCCTTGCATTTATAAATCAAATAAATGGAAAGTGTGTTGTTATGAAA
CATGACCATGTCTTATCTGAAGAAGAAAAAGATCACAATGGCATTAGGAAATGCTTGACTTTAGTTACAG
GAGCGTAAGGGCACAAGAACAGATGATGGTGCCCTAATAGCCCTTTTTAAGAATGACAGGGCCCCACCAT
TAGCATCCTTTTATATCCTTGGAAATTGCTCATGCCTCGCTTTTCTGGTTCCTATCTGCCTGACTCTTTT
TTATTTCTCTTTCAATTTCATCCGCGCTCGGATAAGCGCATGCTAACACAATATGATTGAGCTGTAAAAT
GGAACGCTGTCGCCTCTAATCTCTTTTATGTAGATATGGAGGTACTTCCACACTCCACTTCATTCTCTCT
CCATTGTTGGCCTTTTTAGAATGTGTTGCCGAGTAATGGAGGCTCAGTCAAATATTAGAGCTAGGTTTCA
CAGGGTGTGATAAGAAGATTATCAGCCCAGCAGTCAGATCTATTGTTGTTCTTTGTGTCAGATTAAAATA
TTCCGATGTACTGTAACTTCGTTAGGAAAATACTGAAGGTGCTTAGGGAGAGACTGATGCTGCTTCATCT
CCTGCCTGATGAGACAAAACCCCTATTAAGTGACCCAAATAAATTGTAAATATGTTTCTGAAGCCTCTGC
AAGGCTTTTGTTGTACATTGTTTTGTTTTATTTCATTTCTACTTTATTATTTAAGTGATAAATAAAAATA
GAGACCTAATTAATTCTATATTCACTGATGGTTGAGCTTCCTATCTTTTGTTTTGTATTTTTGAGAGGAT
CCATTTTATTTGAAATCCGTTGTATTTAAACGCTCTTCTGATATGGGTAGAATTCTACTAATATCCAGCC
ACAGTTTCAGAGTCGTTTAGACACAATCTCTGTAATGTAACTATCTCATTGACATTCAGCAAAAATGTGC
AATGTTCTTTTCATATTAAGTGGAGGCAGGAAACATTAAGCTCTTAAAAATGTGCATAACAGGAAGCTAA
AGACAAAATGTTCTCTAAAAAATTTGCATGCATGAAAAAGCATCAACCAGACCATAGAAATCAACTTTTT
GCACTTAAAATGAAAAATTTGTTTTGAAACAGTAAATCAGACTAATACATCAGAAATAACTAGTTACATT
ATTTAGTAAAACAAGCCTGCTTGTCTTCA
> CNE2_Mouse

TTGAGCCTTCATGGAAAGTGAGAGATTCTACCAAAGAAAATATTAAACAAAAATACATAGGGGGTGAGGG
GTTTTATCAGTAAATAGAGTTAGTTGGTTTGCATTAATAGATCACACAGATGGGACATGAGCTGCTATGA
AACACAGCCACACTATCTACAGAAGGAAAAAGAAAGAAAGAAAGAAAGAAAGAAAGAAAGAAAGAAAGAA
AGAAAGAAAGAAAGAAAGGAAGGAAGGAAGGAAGGAAGGAAGGAAGGAAGAAAGAAAGAAAGAAAGAAAG
AAAGAAAGAAAGAGAGAGAAAGAGAGAGAAAGAGAGAGAAAGAGGAGCACAAGGGCTTCAGAAATGCTTG
ACTTTAGCTGGAGGAGTGTAAGGGCACAAGAACAGATGACGGTGCCCTAATAGCCCTTTTTAAGAATGAC
AGGCCCTGCCATTAGCATCCCTTTATACCCTTGGAAATTGCTCATGCCTTGCTTTTCTGCTTCCTATCTG
CCTGGCTCTTTTTTATTTCTCTTTCAATTTCGTCCGCGCTCGGATAAGCGCGTGCTAACACAATATGATT
GAGCTGTAAAATGGAACGCTGTCGCCTCTAATCTCCTTTATGTAGATATGGAGGTTCGGCACACTCCACT
CCCTCTCCATTGTTGGCCTTTCTTAGAACAGGCTGCCGAGGAACGAGAAAGGAGGCTCCCACAGACAGCC
GAGGAGCTTGGTTTCACAGGGTGTGATAAGGCAATTATCAGCCCAACAGGCAGATCTGGGATTGTTCTTT
GTGGCTGATTAGGATATTATTATGTCCTGGAATTTCCTTAGCAAAATACAGCGTAGGCTTAGCAGGTGGC
ACTTAAAAAGCCGTAGCCCGTATTAGCTGACCCATATAAGTGGCTTGAGCGTATTTATGCATCTTGTCGG
CCGGCCTACTACTTTAAAGGAACAGAAAATGAAT
> CNE2_Human
TTCTTTATAAGAAAGTGAAGGATACAACAAAAGAAGCTATTGAAGAAAATACATATGGAATAGGTTTTTT
TTATCAGTAAATAGAATTAGTTGACTTACATTAATAAATCATATAAATGGGAAAGTGTGTTGTTATGAAA
CATGACCATACTTTATCTCTAGAAGAAAAAAAGATCACAAGGGCTTTAGAAAATGCTTGACTTTAGTTGG
AGGAGCATAAGGGCACAAGAACAGATGATGGTGCCCTAATAGCCCTTTTTAAGAATGACAGGGCCCAGCC
ATTAGCATCCTTTTATATCCTTGGAAATTGCTCATGCCTCGCTTTTCTGGTTCCTATCTGCCTGACTCTT
TTTTATTTCTCTTTCAATTTCGTCCGCGCTCGGATAAGCGCATGCTAACACAATATGATTGAGCTGTAAA
ATGGAACGCTGTCGCCTCTAATCTCTTTTATGTAGATACGGAGGTACTGCACACTCCACTTCACTCCCTC
TCCATTGTTGGCCTTTTTTCGAATGGGTTGCCAAGTAATGGAGGCTCTGTCAAATATTAGAGCTAGGTTT
CACAGGGTGTGATAAGACGATTATCAGCCCAACAGTCAGATCTATTATTGTTCTTTTTGGTTGATTAAAA
TATTATTATGTACTGTAACTTTGTTAGCAAAATGCAGAATGTGCTTAGTCAGATGGTGCTTTAAAAAGCA
TAACCCATGTTAGCTGACCTATATAAATGGCTTAAACATATTTATGGATCTTGCCAGAC

CNE3

> CNE3_Anolis
TCCCTATCTGTTCCTCAAAACATCTGTGGATGTGTCCATTTTATACACACACAAGCGATGGTTCTCTCCC
CCCCCCCCAATGGTGTCCCCCTTTATCTGCTTCAACCAATCTCCAAATGAATGCATGCCTATCATGGCCT
TAGCTTTGACACTCTGGATATATTGATTAATAGTTGCCCTAAGCTCCTTTTAGCAAATCAAATTTTCACT
TTTAGCAAGGATTTCGCCTCTGATTTATTCAGCAAAGTGAAATGCACTAAATTGACTTTGACATTCAGGG
GAAATGGCTGAAAAACAAAGAGGAGAGAAAAGAGATATAAATTGTGTGGCATCTGGATTCACAGAAGAGG
CACCGTTTTCTTTTTCTTTTTTAAAGAGAGATCATTGCTTACTTGAAAACATGATGCATTTAAAGAGAAT
TGCCAACTGCTTGCTGAAACTAAATGTACTGTATAGGCTGCATACTTTTGAC
> CNE3_ElephantShark
GAACACCCCAGCAGCCAGATACCCTTTCTGGCACCCCCTCCATCTGCTTCAACCAATA
TCCAAATGCATCACTGTCTATCAGGGTCTCAGCACTGACACTCTGGATGTATTGATTAATAGTTGCCCTA
AGCTCCTTTTAGCAAATCAAATTTTCACTTTTAGCGAGGATTTCGGTTCTGATTTATTCAGCACAGTGAA
ATGCAGTAAATTGACTTTGACACTCTGGAAAAACGTCGGAAAAACAATGCTTTCAAGTGGAGAGGAAATA
GATATAAAAATGTGTGGCAGCTTGTTTGTTCAGGTTAAAGAGACAATATTCCTTTTTTGTTTAATGGAAT
> CNE3_Zebrafish
CCCATATCCCCACTCCAACCAATCTCCAAATGTGGCCTTGTCTGTCACCGCCCTCAGCCTGACACACTGC
AAATATTGATTAATAGATGCCCTAAGCTCCTTTTAGCAAATCAAATTTTCACTTTTAGCAGGGGTTTCAC
TCCTGATACATTCAGCAAAGTGAGACACACTAAATTAAGCCTGACACTCAGAGAAAAAGGCTGGATGGAC
AGAGCAAAGAT
> CNE3_Xenopus
CCCATCATATTCACCTCATTGACCATCCCTTTATCTCTCCCAGACAGTCTCCCAGTGATAGAATGTCTGT
CATCTCCTTGGATCTGACACTCTACAGATATTGATTAATAGTCGCCTGAAGCTCCTTTTAGCAAATCAAA
TTTTCACTTGTAGCAAGGAACTCACCTCTGATTTATTCAGCAAAGTGCAATGCACCAAATTGACTTTGAC
ATTCAAGTGGAAACAGTAACAAACATGCGAGGGAACCGAAA
> CNE3_Chicken
CACCCATGCCCCCAACACACATACTACACTGTCTGCCGTTGCACGTTTTTTTCCCTCCCAGTGTCCCCCT
TTATCTGCCTCAACCAATCTCCAAATGAATGCATGTCTATCATGGCCTTAGCTTTGACACTCTGGATATA
TTGATTAATAGTTGCCCTAAGCTCCTTTTAGCAAATCAAATTTTCACTTTTAGCAAGGATTTCGCCTCTG
ATTTATTCAGCAAAGTGAAATGCACTAAATTGACTTTGACATTCAGGGAAAATGGCTGAAAAACAAAGAG
GGGAGGAAAGAGATATAAATTGTGTGGCAGCTGGAGAGTTCAAGCATAAAAGAGGCAGTTTTCTATAGAA
TATCACTGGTTAATTTGGAAACATGCATTTAAAGAGGAGTATAAA
> CNE3_Mouse
GGTGACCGACCCTCGCTCTAACCCCACTCCCTCTTCCCATAGTGGCTAGTCACACTTTCCCTCTGTGTTC
CCCTTCATCTGCCTCAGCCAATCTCCAAATGAATGCGTGTCTATCCCGGCCTTGGCTTTGACACTCTGGA
TATATTGATTAATAGTTGCCCTAAGCTCCTTTTAGCAAATCAAATTTTCACTTTTAGCAAGGATTTCGCC
TCTGATTTATTCAGCAAAGTGAAATGCACTAAATTGACTTTGACATTCAGGGAAAATGGCAGAAAAACAA
AGAGCCCAGGAAAGAGATATAAATTGTGTGGCAGCTGGAGACTTCAGGCACCAGAGAAATGGTTTTCTAG
AGAATTCCATGTCATTTAAAAACACTTTTTAAATACAACAGTGACTTATTTAATTTGTTTTTAACTGTTG
AGTTTTCTTTTAGGAAGAT
> CNE3_Human
TTTGCTCCTTTTTGACCCTCTCTCCAACCCTAATCCCACTTCCCTTCAGTGGGCTGGTCGCACTTTTTCC
CCTGCGTCCCCCTTTATCTGCCTCAGCCAATCTCCAAATGAATGCGTGTCTATCCTGGCCTTGGCTTTGA
CACTCTGGATATATTGATTAATAGTTGCCCTAAGCTCCTTTTAGCAAATCAAATTTTCACTTTTAGCAAG
GATTTCGCCTCTGATTTATTCAGCAAAGTGAAATGCACTAAATTGACTTTGACATTCAGGGAAAATGGCT
GAAAAACAAAGAGCAGAGGAAAGAGATATAAATTGTGTGGCAGCTGGAGACATCAGGCATCCGAGAAACA
GTTTTCTATAGAATACAGTCATTTAAAAGCACTTTTTAAACAGGCAGAGAAGTGATTTCTTAAAATTGTT
TTAACTATTCAGGTTTTTTTTTTTTTT

CNE4

```
> CNE4_Anolis
TTAACTGTAAAACTAACCAGGTCACAGCTAATCATGCAGTACTTTGTCTAACAGAAGTGGGTGGAGGATT
TAAGGTGAGCCTTAATGCCACAGGTTGATGGTATAAATCATGGAAAGCGGGGACAGGGACACAATATTTA
CTTAGACTGGATTTAGAGCAGACAGAGTCTATACCTACATGACTAGGTCAGCGGAATGAACCAGTCTAGT
TGCTTGAGCTAAGCTGTCCCCAATTAAATACTTTAAAGAGCTTCTACTTTCTCTCTGGGTGCTGCAGGTT
CCCCACCTGCTTAACCTAGTTTGGAATAGGATTAATTACTTGAGTTACAAGGCCCCCACAGGCCATCAGC
ACGTTTCCAGCAGGGCACATGCCTTGACCTAGGTGCTGGCACAGGGAAATGGAGCAGAGGGAGATCTAGG
TGCTCCAGTCTAAGCAGAGGAAGGTAATTGTGTATGCTTGGGATTTTGTAAGATTGACTTTTAAGGCAGT
TCTTTGGATCGTGTAGTAAACGTCTTGGGGGACTTCCTTCCACCAGAAAACAAATGTGGTTCCTGTTCCT
AGTGCTGATATTTCATTATTTCA
> CNE4_ElephantShark
GGGTAGATATGCAGCTACTTAGCGTGCATTAGAAGGGATTAATCACCTGTACTGAGGAGTGTTAAAACAC
GTCAGCACGTTCCTAACAAGGTACATGTTGACCTGGCTGGAAAGGAATTTGGT
> CNE4_Xenopus
AGACCTGTGCTTTGAGACTTCTTAGAATCCAGCCTGAAAATGACTCCTTTTCTTTATAGTTGTCTTCACT
CCCACTTAAGCCACATTGGAAAAAGGGATTAATTACACGGATAAAGTAGCTGCTAGTGGCCAGTCAGCAT
GTTTCTGCCAGGGTCACGCAGTGCCCCCGAGTGTATATAGTGCGTGTCAGGGAAAGACAGAGTGCCGGGG
TCACCC
> CNE4_Chicken
GAAGGAGAAGCAGTGCCCGGCCTGGATTTAGAGCAGACAGGTCTCCGCCTGTATGACTGGGTCAGCAGCA
CAGGATGGTCTAGTTTCCTAAAGTCAGCTGAGACCGGCTAAATGCTGTAATTCTGAGGATGCCTCCTCTA
CTTTCTCTCTGGATGAGTTAGGCTTCACACCTGCTTAACCTACATTAGAATAGGATTAATTACCTGAATT
GCGAGGCCCCCTTAGCCAGTCAGCACGTTTCTGGCAGGGCACATGGTGACCTGGGCCTGGTTATATATAG
GATAGAGATAGGCAGGGGGAGCATCTGG
> CNE4_Mouse
CACACATGATGTCCTCACCACAGGTTCTTATGTTAATATTATAGCACTTAGAAGGAGAGAAGAAACGATT
CTTCAGCTGGATTTAGGTGAAACGTGTTGGTAATTGAATGTGCAGGTCAGCATCAGAGCCTGCCCAGCTT
CAGGGAATCATGTGAGCCCAATTAAATCCCATCACTCCAGGGGCATCTCCAAAGAGCCGCTACCTCCTCT
CTATATGAGGAAGGCTTCCCACCTGCTTAACCTGCATTAGGAAAGGATTAATTACCTGGGGTGTGGGCCC
CTCTGCCAGTCAGCAGGTTTGTGACAGGGCACGTGGTGGCCTGCACTGCTGAGGGATGGAAGGAGGGAGG
CAGGAGGGGAAGAGAAGCAGCTGGGGCCTCAGAGATAGGCTGCCTCCTCTCATCCTGATCAGCACTTTAG
GAGAGACAAACGCAAAAGGAAGTCTGCTCTTACGTGGCGATTGTCTGTGTCATGGTGGGGGCTGTGACTG
G
> CNE4_Human
CACAAATTGTTAATATTAGAAAGTCCAGAAGGGGAAAAGACCAATTTCTTCAACTGGATTTCACTTAAGC
AAGTTGAAAACTGAATGTCCAGGTCAGCAACACACCTACCCAGTTTCAGAGAATCATCTGAGCCCAATTA
AATACCATAACACTAGGGGTGTCTCCAAAGAGCAGCCACCTCCTCTCTGTATGAGGGAGGCTTCACACCT
GCTTAACCTACATTAGGAAAGGATTAATTACCTGAAGTGCTGGGCCTCCTCCACCAGTCAGCACGTTTGT
GCCAGGGCACATGGTGGCCTGGGCTGCAGAGGGAGAGAGGGAGAAGGAGCAGCTGTGAGTTCCTGGAGAA
ACTAACTCTTCCTGACATGGTCAGCATTTTAGGAAAAAAAAAAAAATAAGTCAAGCTGCTCTTATTCTTGT
GATTTGTCTGTGTCATGGCAAA
```

CNE5

```
> CNE5_Anolis
GCAATTTATGTCTGCTTATTTTTTGTTCAGTTTTTTAAAAAAGAAAAAGAAAAAAATAAGAAGATTGTTG
GATTTGCAGCAGTTCCCATGCTGTTCCAAACACTAGCCCTTTTAATGTATCTGAACCATTCTGAAGTGCC
TGCTCTATGGTACATCCTTACTTGGCTTGTTATCCTCTTATCAGCCTCTTTGCTTCCCTTTGTTTATTTG
AGGAGACAGGAAGACTGTGTGAAGGCTGCTTAGTCAGGGATATCAGCGTTGTTGGCAGGGGAGTGATTGC
CAGTTGAAAAACAAGGCATTATTGCTCATTTTTTCCCTCTTATTAGTTTGATTACATTTGCAAATCAAAT
CAATCCATCAGACATGATCAGGCCTCGTCCGCATTTTAAGGAATAGTAATCTCTGTCTAATAAGATGCAA
ATGTGTCACATCGGTAAGTAACCAATAAATCATCGTCTTGTCTTTGGCAATCATTAAATATCAGAACCAA
CAGTCAATTTTTAGTATTTTCAATTTGCGATATGCCGCTGGAATATAAAGATAGATGGACGTAATTACAC
AAACCAAACACTATTTCAGTTCATTCCAGACAGCAAATTTAGTGGAAGGTATAACTGGCTTGTCATCACA
CTACATTATTTCTGGCGGTTCATGCAAAGTTCACAGACTAGCTTCAAATGGACACTGCTATGCACACACA
CACACACACACACACACTAAACTTCATGAGATGTCTTAAGTACAAATATGTATCTCATCTACTGATAGAC
AGAACTACATCAAAACAGGAACATGGTTACATTCTTGTACAATCACATGATGGCATTTACTAGTACAGTA
AAGTATTTGTTTAGAAAATGTTCACAATATTTGT
> CNE5_ElephantShark
ACGAAGCGACAAGAAATGCTCTTTCTGAGGCTGCTTAGTCAGGGATATCACCCTGTTGGCAGGCAGTGAT
TGCCAGGCGAAGAAAACAAGGTATTATTGCACATTTTTTCCCTCTTATTAGTTTGATTACATTTGCAAAT
CAAATCAATCCATCAGTCGTGATCAGCTGTGTTCAGAATTCAAGGAATGGTAATCTTCTTCTAATAAGAT
GCAAATGTGTCACATCAGTAAGTAACCAATAAATCATTGGTATGTCATTGGCAATCATTAAATATCAGAA
TGAACAGTCAATTTTTAGTATTTTCAATTTGCGATATGCCTCTGGAATATAAAGATAGATGGACGTAATT
ACACAAACCAAACACTATTTCAGTTCATTCCAGACAGCAAATTTACTGTAAGGAATAACTTGGCTGTCAT
CACAGTACATTATTCCTCACAGTTCATGCAAAGTTCACAGACAAGCATGAAATGGCCATTTCTTTCCCCA
ATAACTGTTCAAATGGGCCATCCTTT
> CNE5_Zebrafish
GCATTCGTACCAACCCATCAGGCCCTGTGTCTGTCCCACAGCCAGCGGGGGGCGGCCCTGTGTGATTGCC
CGTTGAAAAAACAAGGCATTATTGCTCATTTTTCCCTCTTATTAGTTTGATTACATTTGCAAATCAAATC
AATCCATCAGGCAGTTTGTGAAGGGCCGCGTCCTGCAATCTGAAAGGGCCTCCTAGTAATCTCCATCTAA
TAAGATGCAAATGTGTCACATCAGTAAGTAACCAATAAATCATCGGCGCGTCTACGCCAATCATTAACCA
TCAGAATGAACAGTCAATTTTTAGTATTTTCAATTTGTGATATGCTAGTGGAATATAAAGATAGATGGAC
```

GTAATTATACAAACCAAACTCTATTTCAATCCTCCCATCTCCAGAAGCAGATTTGCTGGGAGGTAGAACA
GGCCTGTCATCTTATTATAGTATTTTTCTTTTCTTTTTTTTTTCTTTTTGGTGGTCTGGAGGTTCAAGCA
AAGTTCACAGACAGCCTTGAAACGACCCTCCCATAAAGAAGGCAAC
> CNE5_Xenopus
ATATTACAGTCATTGGAACCCGTCAGCGGCAGGACCAGCGGAAAAGCTATAATCTTCATGTGCTTCCCTA
GTAGGTTTGGGATCCTCTTATCAAAGCTCTTTGCATCCTTTTGTTTATTGAAGAAGACAGAAAGACGGTG
TCGAGGCTGCTTACTCAGGGATATCATGCTTGTTGGCAGAGAAGTGATTGCCAGTTGAAAAAACAAGGCA
TTATTGCTCATTTTTCCCCTCTTATTAGTTTGATTACATTTGCAAATCAAATCAATCCATCAGACATGAT
CAGGCCGCGTCCGCGTTTCAAGCAATAGTAATCTCCGTCTAATAAGATGCAAATGTGTCACATCGGAAAG
TAACCAATAAATCACGGCCTTGTCTTTGGCAATCATTAAATATCAGAACCAACAGTCAATTTTTAGTATT
TTCAATTTGCGATATGCCGCTGGAATATAAAGATAGATGGACGTAATTACACAAAACCAAACACTATTTC
AGTTCATTCCAGACAGCAAATTTAGTGGAAGGTATAACTGGCTTGTCATCACACTACATTATTTCTGCCG
GTTCATGCAAAGTTCACAGACAAGCTTCAAATGGACATCGGATTTTTTATTTTTTTAACTTCCCCTATGT
CCCTATCGCGACCCAAACTTAAATAAATGACTTAATTGGGGCATCATTTTCATCGGTGCCAGGAAGGCAT
AAAGCAAGAAATATTACGGCGTTT
> CNE5_Chicken
GCTTACTGCTTTTTAAGGAGAGAAGGCACAATAAACCCTGTCCTGGCACTTCCATTGATACATTTGTACA
GTGCTGAGGTACAGGCTCCATGTTACATCCATACTTGGCTCGTTATCCTCTTATCAAGCTCTTTGCATCC
CTTTGTTTATTTGAGGAGACAGGAAGACTGTGTGAAGGCTGCTTAGTCAGGGATATCAGCGTTGTTGGCA
GGGGAGTGATTGCCAGTTGAAAAACAAGGCATTATTGCTCATTTTTTCCCTCTTATTAGTTTGATTACAT
TTGCAAATCAAATCAATCCATCAGACATGATCAGGCCTCGTCCGCATTTTAAGGAATAGTAATCTCCGTC
TAATAAGATGCAAATGTGTCACATCGGTAAGTAACCAATAAATCATCGTCTTGTCTTTGGCAATCATTAA
ATATCAGAACCAACAGTCAATTTTTAGTATTTTCAATTTGCGATATGCCGCTGGAATATAAAGATAGATG
GACGTAATTACACAAACCAAACACTATTTCAGTTCATTCCAGACAGCAAATTTAGTGGAAGGTATAACTG
GCTTGTCATCACACTACATTATTCCTGGCGGTTCATGCAAAGTTCACAGACAAGCTTCAAATGGACATTG
CTTTTTCTACTTCCCTTAACAACATTCCCACTACCACCACCAAACTTCGATAAATGATTTGCAGTGCTGT
AGGACATCACTCCCATCAGTGCACA
> CNE5_Mouse
AGATTCATTCACTGTGGTCCAATATGGGCCACCGAGCTGGCTATCTCTGTCCTTCTCAGGACTGCTATGG
TCCTGTTTCCTCCTGCTCTCAGCGGTTAACTTTTGTCCCTTTGCGGGGCTCTGCTCAGATGGTGGGCTCC
ATGTTGCATCCGCACTTGGCTTGTTACCCTCTTCTCAGGCTCTTAGCTTCCCTTTGTTTATTTGAGGCGA
CAGGAAGACGGCGTAAAGGCTGCTTAGTCAGGGATATCAGCCTTGTTGGCAGGGGAGTGATTGCCAGTTG
AAAAACAAGGCATTATTGCTCATTTTTTCCCTCTTATTAGTTTGATTACATTTGCAAATCAAATCAATCC
ATCAGACATGATCAGGCCTCGTCCGCATTTTAAGGAATAGTAATCTCCGCCTAATAAGATGCAAATGTGT
CACATCGGTAAGTAACCAATAAATCATGGTCTTGTCTTTGGCAATCATTAAATATCAGAACCAACAGTCA
ATTTTTAGTATTTTCAATTTGCGATATGCCGCTGGAATATAAAGATAGATGGACGTAATTACACAAACCA
AACACTATTTCAGTTCATTCCAGACAGCAAATTTAGTGGAAGGTATAACTGGCCTGTCATCTCCCCGCAT
TATTCCTGCCGGTTCATGCAAAGTTCACCGACAAGCTTCAGATGGACATTGCTTTTCCCCCCCCCCCCAA
CTCCTAACAACGTCCCCACAATGGCAGACAAACTTTGATAGAAGGGACATGAACTGAACTTCCTGATGGC
CCAAGTCGGATGCCAACATGACAAGAAGGAAGCATGTATTCTCATCCATCAGAGAGACTGCTGCCAGTGC
TGGCTCTCACTGCTTCTGTGCTCCCTCTCTAAGAAAATGAACTCACCATTGTAAAAATACAGGAAAACCA
CCGCAGCCAAACATGCTCGTGTGATTTATGCAGGCCTCGTCTGTTTTGATGGAATACTTTTCCTGGGGTG
CAGGGAATGAATAAACCCTGAATTTTTCTAAGTTTCACACTTTGGCACACCCTTGCATAGGTAAAGGGTT
CTTTTTACCTGTGCCATTCTCTGTGAGTATATGTAGATTTATAGTCTGCAACATGTACATTGAAGGGACA
GTTTATTCATGTTTGACATACGTGTGTCTGTGTGTTTTGCAGAGGCCAGAAGTGAAAAT
> CNE5_Human
CATTGTTTTTCTCAAGATTGTTAAGGTTGTGTTACATCTCCTGCCGTTGACTTTTGTTCTCTCCGGCAAC
AGCACAGTTCAGAGGTGTGGGCTCCATGTTGCGTCCATACTTGGCTTGTTATCCTCTTATCAAGCTCTTA
GCATCCCTTTGTTTATTTGAGGTGACAGGAAGACTGTGTAAAGGCTGCTTAGTCAGGGATATCAGCCTTG
TTGGCAGCGGAGTGATTGCCAGTTGAAAAACAAGGCATTATTGCTCATTTTTTCCCTCTTATTAGTTTGA
TTACATTTGCAAATCAAATCAATCCATCAGACATGATCAGGCCTCGTCCGCATTTTAAGGAATAGTAATC
TCCGTCTAATAAGATGCAAATGTGTCACATCGGTAAGTAACCAATAAATCATCGTCTTGTCTTTGGCAAT
CATTAAATATCAGAACCAACAGTCAATTTTTAGTATTTTCAATTTGCGATATGCCGCTGGAATATAAAGA
TAGATGGACGTAATTACACAAACCAAACACTATTTCAGTTCATTCCAGACAGCAAATTTAGTGGAAGGTA
TAACTGGCTTGTCATCACACTACATTATTCCTAGCGGTTCATGCAAAGTTCACAGACAAGCTTCAAATGG
ACATTGCTTTTTTTCAACTTCTAACAACGTCCCCACAATGGCAAGCAAACTTTGATGTAAGGACTTGAAC
TGACTTCCGCACGGCTGTTGCAGTCAGATGCCAACATGACAGAAAACATATGCTCTCTCCCATCAGAAAC
TGCTGGCAGTGCCAGCTCCAATCTTCTATATTTCCTCTCTTAAAAAAAATGAACTTACAAGTGTAAAAAT
AAAGAAAAACAAAAACAATCAAGAACTCTTCAGGTGATTTATGCCATCCTCATGTATTTGGATGGAATGG
TTTTCCTTCAGGGGGAAAATGTGAACAAAAACTTTATTTTTCTAAGTTTCACATTTTAGAGCTTCTCTGT
GTGGGCAATGTATTCTTTTGATCTAAGCCATTCTCTGTGAAGACGTGC
> CNE5_Lamprey
CGCGGAGTGAGCCCTCCGTGACAAGAGCGATTGCCGCTCGCAAAACACGGCGTTATTGCGCATTTTCCTC
GTGGTTAGTTCGATCGCATTTGCAAATCAAATCAATTCATCAGTTTCTATCATCGTCATTAATAACACTC
GCGGTTTGCAATCTTCTTGTAATAAGATGCAAATGTGTGTTGAAAGCGAGTTACCTATAAATCACCATCC
TGTCGGTGGCAATCATTAAATATCAGAATGCACAGTCAATTTTTAGTATTTTCAATTTACCGTATGCCTT
CTGGAATATAAAGATAGATGGATGTAATTACACAAACCAAACACTATTTCAATTCACCTTGGACAGCAAA
TTTAGTGTAAGGAATAACATTCCTGTCATCACGGCCCATTATTTCATGTGGTTCAGGCAAAGTTCACAAG
CAGGCACAGAGAGAGAGGGGCAGCATTGTC

CNE6

> CNE6_Anolis
TCTTCTAGCCTTCAATTGCCTCATTAGAAATCTATAGGATGACAGTGGCTGAAACTGAAAAAAAAGATTG
TTAGTCATAATATCCCTACTTAATTTTATGTTGAGTTAGAGGAAAATGTTGAATTCTTACTCCTGCAAAG
CTTTCCCTGGTGTCTCATCCTCTCTCCTCTATCATCCTGAGGCAATGTGCCTGCATTTGTGCGTAGATTT

ATAGTTTATCTTTATCTGTGGCTTCATCGGTAGGTAGCTATTATAGCCGCCTTGCCTGGATATTGTTTGG
GCCTGATAGATCTGTCTGTAGTTCATCAAAGGGAAGCTGAGTGGCTAAAAGCCACTGGGTCCAGCTGAGG
ATGAAATACGAGCCATCAGCCTTGGTAATGGCTGTAAAATTTCATAATTACACGGCCTTTATTACATTGC
ATAATGATCCTGAAGCAGTATGGAACAATTAATTAAGATTTTAAACCATGGCTCTTTCAGAAATTAAAAG
GTGCTAGTGGTTGAAAAATGAGGGGAAACGTCAGGTCCTAAAAGCATTAGGGATGCTTAAGAAGTACTTC
TAGGGTACTCTCTCTGGAAAGTCACAAATACCAAGAAGTACTTCTTAGGAGAGCTAATGTTAGGGGTAAC
TATGAACTGGCTTTGATCTGGGTTTTAAGTTTCAATCAGGGTTTGAAAGTGATCAGAACAGATGCCAGCA
GCTTTTA
> CNE6_ElephantShark
GCTAACGGATAGCTGGAGAGGGATGTCTAATGCACAGCTGCTGTCTCCCCGAGACAGCTCATCCTCTCCT
TTTGTCAATTCAAGGCATTGTGGTTGCATTTGTGCAGGGATTTATAGTTTATCTTTATCTATTGCTTCAT
CTGTAGGTAGCTATTATAGATGCCTGGGCTGGTTATTGTTTGGGGGTGATAGATCTGTCTGTACTTCATC
AAAGAAACTGGAGTAGCTAAAAGCCATTGGGTGAAGCTTATGATGAAATACGAGCCATCAGGCTCAGTAA
TGGCTGTAAAATTTCATAATTACATGTCTTTTATTACATTGCATAATGATCCTGAAGCAGTATGAAACAA
TTAATTAAGATTTTGAACCATGGCTTTTTTGAAATTAAAAGGTACTCGGTCTGTGAAATGGGGGAGAGGC
TTCCTCTAAAGCATGAAGGAGACGGGTGTTCAGGGAATCTGATTTATTGAGCC
> CNE6_Zebrafish
AGCCGCTAAACAGCATTTGGGTTTGTGCTGTGGATTTATAGTTTATCTTTATCTGTTGCTTCATCTGTGG
GTAGCTATTACGCCAGCCTGGCCAGCTTATTGTTTGGGCCTGATAGATCTGTGTGCAGTCCATCACTTGG
AGCCGTGCGCCCGGAAAGCCATCGGCCCGCCTGATGATGAAATACGAGCTATCAGCCTCAGTAATGGCTG
TAAAATTTCATAATTACACGGCCTTTATTACATTGCATAATGACCCTGAAGCAGTATGGAACAATTAATT
AAGATTTTCAGCCGTGGCTTTTGCAGAAATTAAAAGGTGCTCATTGCCGTGGAATGGGGTAATGTCTTGT
CTCGGAAGCATTAAGGCGCGTTTTAAGCGCGAGACTCCCTGTCTGTCTGCGTG
> CNE6_Xenopus
GGAACAAGAGAGGAGCTATATGGAAGCCCTAATGCAGACGGCTCTCTTCCCCAACAGTTTCATCCTTTTT
CCTTTCTGTCATCCTGAGGCAATGCGCCTGCGTTTGTGTGTAGATTTATAGTTTATCTTTATCTATGGCT
TCATCTGTAGGTAGCTATTATAGCTGCCTTGCTTGGATATTGTTCGGGCCTGATAGATCTGTCTGTAGTT
CATCAAAGGGAAGCAGAGTGGCTAAAAGCCGTTGGGTCCAGCTGATGATGAAATACGAGCCATCAGCCTT
GGTAATGGCTGTAAAATTTCATAATTACACGGCCTTTATTACATTGCATAATGATCCTGAAGCAGTATGG
AACAATTAATTAAGATTTTCAAGCGTGGCTCTTTCAGAAATTAAAAGGTGCCAGCGACTGAGAAACAGGG
AAATGTCTGTCTCTAAAAGCATTAGCAATGCTCTTGAATATGTGCCGTGCCCCTCTGTCTTTACAGTATT
ACATGGTCATAGTTGGATAAT
> CNE6_Chicken
GAAAAGAGCGAGAGAGAAAGAGGAAGGATTTCCTTTCTGCGAGCCGCCAGCTTTCCCAAGTGCCTCATCC
TCTCCCCTCTGTCATCCTGAGGCAATGTGCCTGCATTTGTGTGTAGATTTATAGTTTATCTTTATCTATG
GCTTCATCTGTAGGTAGCTATTATAGCCGCCTTGCCTGGATATTGTTTGGGCCTGATAGATCTGTCTGTA
GTTCATCAAAGGGGAGCTGAGTGGCTAAAAGCCATTGGGTCCAGCTGAGGATGAAATACGAGCCATCAGC
CTTGGTAATGGCTGTAAAATTTCATAATTACACGGCCTTTATTACATTGCATAATGATCCTGAAGCAGTA
TGGAACAATTAATTAAGATTTTCAACCGTGGCTCTTTCAGAAATTAAAAGGTGCTAGTGGCTGAGAAATG
GGGAAGTGTCTTGTCCTAAAAGCATTAGGGATGCTTAAGAAGTACTTCCTGGGTACT
> CNE6_Mouse
TAAATGAGACGCTCTCTTGGGCTTGGGCTTTTCTCTCTTTTTTATAACACCCGAGCCATTGTGGAGCAGG
AGAAGCCCAGGAGGCCGCCTCCGTGGGAGCGGCCACTCCCAAGCGTCCCATCCTCTCCTCTGTCATCCCC
AGGCGACGCACTGCATTTGTGTGTGCAGATTTATAGTTTATCTTTATCTCTGGCTTCATCTGTAGGTAGC
TATTAGAGCCGCCTCGCCCGGATATTGTTTGGGCCTGATAGATCTGTCCGTAGTTCATCAAAGGGGAGCC
GAGTGCCTAAAAGCCATCGAGTCCAGCTGAGGATGAAATACGAGCCATCAGCCTTGGTAATGGCTGTAAA
ATTTCATAATTACACGGCCTTTATTACGTTGCATAATGATCCTGAAGCAGTATGGAACAATTAATTAAGA
TTTTCAACCGTGGCTCTTTCAGAAATTAAAAGGTACTGGGAGCTGAGAAATGGGGATCTGTCTTGCCCTG
GAAGCATTAGGGATGCTAAAGGAAGGGCTTGGCTGCATACTTATTTTTCCTTAAGAGGAAAGCACCTCTT
GGTTGCGGAGCACGCATTAGTATCCCTTGCCTATTTC
> CNE6_Human
TAAAATCCACCCCATTCTGGAGTGAATGAGCGAGAAGCACAGGAGGTGGCCTTCGTGGGAGCAACTACCT
TTCCCAAGTGTCTCATCCTCTCTCCTCTGTCATCCCCAGGCAATGTGCCTGCATTTGTGTGTGTAGATTT
ATAGTTTATCTTTATCTATGGCTTCATCTGTAGGTAGCTATTATAGCTGCCTCGCCTGGATATTGTTTGG
GCCTGATAGATCTGTCTGTAGTTCATCAAAGAGGAGCCTAGTGCCTAAAAGCCATTGGGTCCAGCTGAGG
ATGAATTACGAGCCATCAGCCTTGATAATGGCTGTAAAATTTCATAATTACATGGCCTTTATTACATTGC
ATAATGATCCTGAAGCAGTATGGAACAATTAATTAAGATTTTCAACCGTGGCTCTTTCAGAAATTAAAAG
GTGCTAGTAGCTGAAAAATGGGATCTGTCTTGTCCTAAAAGCATTAGGGATGCTTAGGAAATACTTTCCA
ATTGCACTTTTATTTTTTGTTAAGAGGAACATCCGCTAGTTAGGGAACAT
> CNE6_Lamprey
CTAATATTTACTGTGAAATATGAGCCGTCGCCATCTGTAATGGCTGTAAAATTTCATAATTACATGTCCT
TTATTGCATTGCGTAATGATCCTGAAGCGGCGCAAAAGAATAATTAATTAGGAGATTATGAGCCGTAGAT
TCGGAGAAT

CNE7

> CNE7_Anolis
AATACTTTTGCTTCTAAAATATACCCATCTTGGCTAATTTAATCTAAATTTCTATCACAAGATCACAATA
ATGACAGTGTGATATGGACTAGTCACATAATGTGTTATGCTTTTGAGTGTTTAGTGGCATAGTAAGTGTT
CTCTTGGGTGTCTTTTAATTATAAACTTTTGCCAGAAAATTTGGAACCGATTATGTGCTTCATTCAAGGA
GGACCTGGCTGCTTCAAGTCAATTCTTTTGTTGAGTCATTTATTCCCTTAATAGGAATTTTCTGATCGGC
TGTCATTCTTGTTCACACACTGGGTGTCACTGCCTCTAAAACACTTGTCATTCTTTAATGGAAACAAAAT
AGTCATTGCTGTCAGAGCTGCAATGTCATTCCCCTACCCCTTGCTGCGTCGGCAATAGTGAAAATGACAG
AGCGGCTCTCAGTAGGGGTAATTAAAATGTAAATATTGATATTTTATTATTTGAAATTACTTTCCAGTGC
GGCATTTAATATCTCTCCATCTGTGCGGCTCTGTTTAGCGGTCACTTTAGTGCAGCTCTGGGATGCCAAG
TGGCGGCTTGATCCCCGTTATGTCAAAATGCTTGTTGCTGCTTAATTTTGATATCTAATTAAGTGGAAAA

GTACAGTCCACACTGTAATCCATGGCATCTTAACCAGCTGCTTGGGTATATTTAGAATTAATCTCCACTA
TGGAATCATCCTAATGTATGCAATTAAGAGTCTGCCGATGGCTAATTAGGTGTATTAGAATCAGGCAGCT
TCCACCCCCCCCTTTCTTTCTTTGAACTGTTGCAGACTTCAGACACTCTGCCTCCTGAAGTAGTCTTAA
GAACACCAATACACTCAGGCAAGGGATGCCCAAACCTCTTTAACTTAAATTGGAAATTGGGATGTTGGGT
ACTGAGACACTCCTGCCATTTC
> CNE7_ElephantShark
TGGTGTTATTCTTGCAAAAGTCTGTGTCTCGTTGACTCTGATCACTTGGCATTCCTTAATGGAAACAAAA
TAGTCCTCCCAGTAAGAAGTCCAATGTCATTTCACTACCCCATGCTGTCTTAGCAATAGTCAAATGACAG
AGTGATTCTCAGTAGGAGTAATTAAAATGTAAATATTGATATTTTATTATTTGAAATTACTTTCCAGTAC
AGCATTTAATGTTGCTCCATCTGTGCAGCTCTGTTTAGTGGTCACTTAAGTGCAGCTCTGGGACATGAAG
CAGCAGTTCAATCCTGTCATGTCAAAATGCTTGTTCCTGCTTAATTTTGATATCTAATTAAGTGGAAAAG
TGCAGTCCATACTGTAATCCAGGCATCTTAAACAGCTGCTTGGGTATATTTAGAATTAATCTTGGCTATG
AATTGTCTTAATGTATGCAATTAAGAGTCTGTGCATTCCTAATTAGGTATATTAGAATCATGCAGCTTAC
CATCCCAGTATAGACTTTGGGATGTCTGCCTCGAGAGCAAATGATAA
> CNE7_Zebrafish
AATAGGAAAAAGGTGTAATTAAAATGTAAATATTGATATTTCATTGTTGGAAATGGCTGTGGTTAATGTC
ACTCCATCTGTGGAGCCTCTGCCATGGATGCTTTATTACAGTCCGGGGAGCAGCCGCCCTTCTCCGCCGC
CACCAGGGCACATGTGCTTGTTGCTGCCTCGTTTCAATACGCAATTAAGTGGAAAAGTACAGTCCGAACA
GTAATCCATGCTATCTTAAGCAGCTGTCTGGGTATATTTAGAATTAATCTCCACCACCAATCGGCCTTAA
TGTATGCAATTAAGAGTCTGCCGATTGCTAATTAGGTGCATTAGAATCAAGAAGCCATGCTAACGCGACA
ATGCAG
> CNE7_Xenopus
AGTGTGAACTGCTGCTTTCAGCCAGAAAGAGAAAATAAGAAAAAAAACATTTTGAAAATGAATATTTGTG
TCAAGAGGGGATTAGCTTTGTAAAGTCAATTTCCTTTTCGGGAAATGTATTCCCTTAATAGGACTTTTCC
GATCCACTGTCATTCTTGTTCACACACTGGGTGTCACTGCCTTTAAAACGCTTGTCACTCTTTAATGGAA
ACAAAACAGTCTTTGCAGTCGGTGCTGCAATGTCATTCCCTTACCCCTTGCTGCGTCGGCAATCGTGAAA
ATGACAGAGCGCCTCTCGGTAGGGGTAATTAAAATGTAAATATTGATATTTTATTATTTGAAATTACTTT
CCAGTGCGGCATTTAATATCTCTCCATCTGTGCCGCTCTGTTTTGTGGTCACTTTAGTGCAGCTCGGGGA
TGGCGGGGAACGGCTTGATCCCCCGTTATGTCAAAATGCTTGTTGCTGCTTAATTTTGATATCTAATTAA
GTGGAAAAGTACAGTCCACACAGTAATCCATGGCATCTTAACCAGCTGCTTGGGTATATTTAGAATTAAT
CTCCACTATGGAATCAACCTAATGTATGCAATTAAGAGTCTGCCGATGGCTAATTAGGTGTATTAGAACC
AGGCAGCTTTCTATCTTTCATTCTTTCTTCTTTCTATTTGTGTGTGGGGGGCTTTTTTTGGACTGTCAAG
GGCTTAACCCCATGGGTATCAGAAAGGT
> CNE7_Chicken
TGTCTAGCTCTTAGACCAAATGAGCCTTGAGTTTTAGTGTTTTTAAATTGACCGGTTATCATGTAAGGTC
CTATCAGTCTAAATATTTTATGAGAAGCTAAGGTTTCCCCTGGGCACCTCTAATAATGAACTTTCGGCAG
GAAATTTAGAAAGGATTATGTGTTTCAAGGAGGAGCTGGCTGCTCGAAGTAAATTCTCTTGTTGAGTCAT
TTATTCCCTTAATAGGAATTTTCTGATCTGCTGTCATTCTTGTTCACACACTGGGTGTCACTGCCTCTAA
AACACTTGTCATTCTTTAATGGAAACAAAATAGTCATTGCAGTCAGAGCCGCAATGTCATTCCACTACCC
CTTGCTGCGTCGGCAATAGTGAAAATGACAGAGCGGCTCTCAGTAGGGGTAATTAAAATGTAAATATTGA
TATTTTATTATTTGAAATTACTTTCCAGTGCGGCATTTAATATCTCTCCATCTGTGCGGCTCTGTTTAGC
GGTCACTTTAGTGCAGCTCTGGGATGCCAAGCAGCGGCTTGATCCCCGTTATGTCAAAATGCTTGTTGCT
GCTTAATTTTGATATCTAATTAAGTGGAAAAGTACAGTCCACACTGTAATCCATGGCATCTTAACCAGCT
GCTTGGGTATATTTAGAATTAATCTCCACTATGGAATCATCCTAATGTATGCAATTAAGTGTCTGCCGAT
GGCTAATTAGGTGTATTAGAATCAGGCAGCTTCCTTTTTTTTCTCTTTCCCCTTCTCCCTTTTTTTTTTC
TTTTGTAT
> CNE7_Mouse
CGTGGTGCTGCTAATGCAAATGAGGGTAAGAAAAAAAAAATCCCAACCCCCTCCCCCGTGATTTGCAGAGA
ACTCCCGCATATTTGCCCCCATCAAGTATTATTGTTTTTAAATTGACAATCATCACATAACGTTTTACAG
TTCTAAATATTTCATGGGAGACTAAAACTTCCCTTGGGCCTCCCCAATAATAAACTTTCAGCAGAAGAGT
CAAAAAGGCTGATGTACTTCAAGGAGGAGTCTGCTGCTCCCAGAGAATTCTCTCACTGAGGCATTTAGCC
CCTTAATATGAATTTTCTGATCGGCTGTCACCCTCACGGGTGCGCCGAGCGTCACTGCCTCCGAAGCACT
TGTCATTCTTTAATGGAAACAAAACAGTCATTGCAGCCAGAGCCACGGTGTCATCTCACAGCCCCTTGCT
GCGTCGGCAATAGTGGAATGACAGAGAGGCCCTCGGTAGGGGTAATTAAAATGTAAATATTGATATTTTA
TTATTTGAAATTACTTTCCAGTGCGGCATTTAATATCTCTCCATCTGTGCGACTCTGTTTAGCGGTCACT
TTACTGCAGCTCTGGGAGTCAGAGCGTAGGCTTGATCCCCGTTATGTCAAAATGCTTGTTGCTGCTTAAT
TTTGATATCTAATTAAGTGGAAAAGTACAGTCCGCACTGTAATCCATGGCATCTTAACCAGCTGCTTGGG
TATATTTAGAATTAATCTCCACTATGGAATCATCCTAATGTATGCAATTAAGGTTCTGCCGATGGCTAAT
TAGGTGTATTAGAATCAGGCAGTGTTTTTTTCTCCCCCTCCCCCTCTCTCTCTTTCCCCTGCTTTCCTTT
TGGACCATTGCCTATGTAAGAGTGCCTGCCTCCAGAGGTGTTCTTAATACCACTTAAATCAAGTTTGGAG
ACGACACAAGTTCCTAATAGATTCATCT
> CNE7_Human
ATTTAAATGAGAGAATATGAAAAAAGAAAACCCCCAACCTCGTTGTGATTTACACATAACTTCTGGATAT
TTGTTCCCGTCAAGTATTATTGTTTTTAAATTGACAATCATCATATAATGTTTTACGGATCTAAATATTT
CATGGGAGACTAAAACTTCCCTTGGGCCTCCCCAATAATAAACTTTCAGCAGAAAATTTGGAAAGGATTA
TGTGCTTCAAGGAGGAGCTGGTGCTCAGAGTAAATTCTCTTGTTGAGGCATTTAGTCCCTTAATAGGAAT
TTTCTGATCTGCTGTCATTCTTACTCACACACCGAGTGTCACTGCCTGTAAAACACTTGTCATTCTTTAA
TGGAAACAAAATAGTCATTGCGGCCAGAGCCGCAATGTCATTCCACTACCCCTTGCTGCGTCGGCAATAG
TGAAAATGACAGAGCAGCTCTCAGTAGGGGTAATTAAAATGTAAATATTGATATTTTATTATTTGAAATT
ACTTTCCAGTGCGGCATTTAATATCCCTCCATCTGTGCAGCTCTGTTTAGCGGTCACTTTAGTGCAGCTC
TGGGATACCAAGCAGCGGCTTGATCCCCGTTATGTCAAAATGCTTGTTGCTGCTTAATTTTGATATCTAA
TTAAGTGGAAAAGTACAGTCCACACTGTAATCCATGGCATCTTAACCAGCTGCTTGGGTATATTTAGAAT
TAATCTCCACTATGGCATCATCCTAATGTATGCAATTAAGAGTCTGCCGATGGCTAATTAGGTGTATTAG
AATCAGGCAGCTTTTTTTTTTTTTCCTCCATTTCTTCTCCCCCTTTCATTTTGGACTGTTGTAGACAGAA
GAGTCTCTGCCTCCAGAGGTAATGTTCATGCTACTCAAATCTAGTTAGGAAACAGGACAACTTCCAAATA
TGGTAACCCTTGTATT
> CNE7_Lamprey

GCAACGCTCGGGATTGGCTGATTAAAATGTAAATGTTGATATTTGGGCACATAAAAGTCGCTTCCCCGTG
TGCCACTTACCGCCCTCCCTGAACGCTCACAAGCTGCGATTCGCGGTGCTTCAAAATTAGTGCCAGCGTA
GCGATCGCCGCCAAAACCCAAACTCTGTTTCTCCCAGCAATTCTGGCCTCTAATTAGGCAAACAGGCGAA
CGATCCAAGCATCCTGAACAGCTGCTCCAGTATATTTAGAATTAATCTCGGCTCAGAATAGTCTAATTTA
TGCAATGGTCTGGAATTACGGCATGCTTAATTAGGCTCA

CNE8

> CNE8_Anolis
GGAGTGATGGGGGAGATGAATCAAGCAACTGTTGTGTTACTCAATCAGTTTCCTCTCAGTGCATAGTTAG
TGATACCAGTGAGTCATTAGGATATAATTACAACTCGAGTGTAAGGTGCAGTTTGTTGAAAAGTCTCTAT
TTATAGTGAACTGGAAGCTGTATTCTACATAGGATGCAGGAAAAAATGTTGACAGGGTGGGGGAGAGAGA
CATAACCAGCAAATCAATGCCATTGTTTATGGCTATTTACAAGAAACTGCGTGGATTTACAGTGCCTTGC
ATTCGGCAATGTAGAAACGGTACAGAAAAGAATGCCTAGCAGGGGTTAAGTTCTTATGCCTGCACTGCAG
CCTATTTCTGTCAACACTGGACTTGGGAAACAAACATTTATGCTTTGTGAAATGAAAGTGTATTTTTGAA
GACTAGCCGTCAGATATTTTTTGCCCTTCACACTTTCAAATGTTTAGCCTTTTTTGTAAGTTGACTGCAA
AGTGCATCAGTTTGCCAGCATCCCTTTTTCTCTCTCCCCCCTCCTCAAAAGCAAAAGAAAGAAAGAAGGG
GGGGGGGTGTAGAATAAGGGGAGGGAGTCTTGACAAGTGCCCATGGCATCATTGTCAAGTACATGAGCTT
CAAGTAGAAGATGGCAGGAGAGTTGGCACAGAACATGCTTCCTTCAAGCCTATTACCTCCGGGGTACCTG
ACTCTTGAGAAGCTTTTCAATCCTTCCAAATGACGTTATTCCAAACTGTCATCTGGTTTATTTGGAGCAC
ACTGTCTCGCAGATAAACATGCTATTTAACTGCAGGTCAAGGTACAGAGTCATTCTGCTAGCAAAATGCC
TGGGATACATTAAATGGCACTGACATGCAGAGGCAGACGGAGAGAGATCAAAGTTGTCACCAGCTTGGAT
GAAATTGGTCTGCACTGGGGGTGAAAATGCACCATGGTATATTGAAGTGCATTATTAAAAGACATAATAT
AGACAAACATTTAAAACTATATGATCTGTCAAATGCTAGGGATTAAGGTACTGTGTAATACAGAACATGA
AGTTCAGGTTCGTGCCATCAATGCCACATC
> CNE8_ElephantShark
TGCATATCTTTCAGAATGAAAGTGCCTTTTAAAGACTAACTGTCATATATATATTTTTTTTGCCCTTCAC
ACAGTCAAATATTTAGCCTTTTAGCAGACTG
> CNE8_Zebrafish
GGACGGCCGGTGGTTGCTGTGAGGCCTGGTGGTGAGCAGTCCTGCCCGGTAGGGGTTAAGTTCGCATGGT
TGCACCGCAGCCTCTTTCTGTCAGCCTGTGCTACTCGCTGCTTCAATGCTTATTTCTTCTGAAAAGCGAC
TGCAGGTTTCACAAGGCCTCAGCCATCAGATACCTTCTACTGGTGTAACCTTCACAGATCCACATACTTA
GCCTTTCCTCCCCATCTCGCACAAAGTGCATCAGTTTACTGAGAGAAAAGAGAGAGATAGAGAGGA
> CNE8_Xenopus
TTGGTGCGCTGGAAATGCTGAAAGCTACCGGAGAATGCCTGAGAGGGGTTAAATTCATATGCCTTCATTG
CAGCCTATTTCTGTCAACACTCGGCTACAAAAATAAACATTTATGTTTTGTGAAGTGAAAGTGTATTTTT
GAAGACTACCCATCAGATATTTTTTTTTTTTGTCCTTCACACTTTCCCGTGCTTAGCCGTTTTTGTTAAG
TTGGCTGCAAAGTGCATCGGTTCGCTTGGCTAAAAAAAAAAATTATAGAATAAAAAAAAACAAAAAAACGA
GGAAAAAATAGCTG
> CNE8_Chicken
GGTATGTGAAAGGAGAAACGTAAGCATATGTTCGGTTGCTCAATCAACCTTCTCTCGATGCACACTTGGT
GATCTCAGCGAGTCATTACGGCATAATTACAATTTGAGTGTGATATGCTGTTTGTAGAAAAGTATCTATT
TTTAGCTGAAGGAATGCTACGGTCCCTGAAAAGGAGAAAGAAAGAAAAAAAGGAAGGGAGAAAATCTGTA
ACCAGCAAATCAATGCTATTGTTAACTGATATTATGAGACACCCTGTAGATTTACAATGCTTGCATGCAG
CCACATAAACCCGTGCTGAAAAGAATGCCTACCAGGGGTTAAATTCATGCGCCTGCACTGCAGCCTATTT
CTGTCAACACTCAGCTCAGGAAATAAACATTTATGCTTTCTGAAATGAAAGTGTATTTTTGAAGACTAGC
CGTCAGATATTTTTTGCCCTTCACACTTTCAAATGCTTAGCCTTTTTTGTAAGTTGACTGCAAAGTGCAT
CGGTTTGCTAGGCAAAAAGGAAAAAAAAAAACCTTGACAAGTGCCCATGGCATCATTGTCAAGCAGAAGA
GTTTCTGGTAGGAGATGGCAGGAGGGTTGGCACAGAACATGCTTCCTTCAAGCTTATTACCTCGGGGGTA
CCTGACTCCTGAGAAGCCTTTCAGTCCTTCCAGATGACGTTATTCCCGGCTGTCATCTGCTTTATCTGGA
GCGCTGCTGCAGCCGAGCACTGCCGAGCTGCGG
> CNE8_Mouse
TATCTATCTATCTATCTATCTATCTATCTATTGTGGAATAGAGAAACTATACTTCAAAACCCGAGAACGA
GCAAATCAATACCGTGATTAACTGATACTAACAGATACCCTGAGGATTTAAATGTTGGCGTTCTGTTTCG
AGCTAAGCAGCTGCCAGGCGAATGCCTAGCAGGGGTTAAACCCAAATGCCTGTGCCGCAGCCTATTTCTG
TCAGCACTCCACTCAGGAAATAAACATTTATGCTTTCTGAAATGAAAGTGTATTTTTGAAGACCAGCCGT
CAGATAGTTTTTGCCCTTCACACTTTCAAATGCTTAGCCTTTTTTTTTGTACATTGACTGTAAAGTGCAT
AAGTTTGCCAGGCTGGGGGAAAAATGTTACTGACAGTGCCCATGGCGTCCCACTCAGTCCCTGAAGGTTC
TGGGAGAAATGGGCAGGAAGGTTGGCACAAAGCATGCTTTCTTCAAGCCTGCTACCTCAGGGGCACCTGT
TGGGAGAAGTTCTTCAATCCTTCCACATGACATTATTCTCCACCATCATCTAGTGTACCGTAGTGCAGAT
AAACACACAATTTAGCTGCAGGTGATGGTAGAGAGCCCTTCTGCCCAGCCAACTGCCTAAGATACAGTAA
ACGGCACTGAGTCCAGGACAGAGATGAAGAGAGATCAGACTTGTCAGCAGTATCAGTGGAAAAGGCTGCC
GACAGGAAAAAGAAAAATGTGCTGTGGTGTATTTTACAAAGCTGTAATAGGACTGAACATTAGAAAGCAT
AGCCTGCCACATGCCTGCCACACGCCAGAGAGTAAGATCCCGGATGCACAGCACGTGAGCTTCAAGGGAC
ACAATGGCAAGTCCCATCCCATTTTGCTTGAGAGAAAGGTATCTTGTCAACGACAGCTACATGGTATAGA
TGACAAGACCTCTAAACACCATGAATGTCTGCTAAGAGCACAAATGAGTAATTATCATCTAGACACAAGT
GTCCATGAGCCAATAACACCATCATGTTTTTATTATTAAAAAA
> CNE8_Human
GAAAGAAAAGAATCTAGCTTACGTTGCATTGCTTAATCAGCTTCTCAAAATGAACTTCATTTGTGATCTC
AGTGAGTCATTATGGCATAATTACAGTTCTAGCATGATATGCTAGTTGTTGAAAAATGTTGATTTTTTTT
TTTTTTGTGGAACAGAAAAACTACAGTTCAAAAACTGTGATCAGGCAAGTCAATGCCATGATTAACTGAT
ATTAACAGATGCCCTGAAGATTTAAAATGCTTGCTCTCTATTTCAAGAAAGCAGCCGAGAAGAAAATGCC
TAGCAGGGGTTAAACCCAAATGCCTGCACTGCAGCCTATTTCTGTCAACACTCTGTTCAGGAAATAAACA
TTTATGCTTTCTGAAATGAAAGTGTATTTTTGAAGACTAGCCGTCAGATATTTTTTGCCCTTCACACTTT
CAAATGCTTAGCCTTTTTTGTAAGTTGACTGCAAAGTGCATCAGTTTGCCAGGCTAAAAAAAAAAAAAAA
CAAAAAAAAAAACAGTGCTTGATGAGTGCCCATGGCACCACTGTCAACCTCCAAAGTTTCCAGTAGAAGA

AGGCAAGACGGTTGGCACAAAACATGCTTTCTTCAAGCCTATTACCTCAGGGGTACCTGCCTCTTGAGAA
GCTTTTCAATCCTTCCAAATGATGTTATTCCCAACTGTCATCTGGTTTATTTGGAGCACACTGTATTGCA
GATAAACATGCTATTTAACTGCGGGTCAAGGTAGAGAGTCATTCTGCAAGCAAAATGCCTGAGATACATT
AAATGGCACTGACTCTGGGACAGAGATGAAGAGAGATCCGAGTTGTCAGCGATATGCAGGGAATGGGCTA
TAGCTGTGGAAGAAAAAATGTGCCATGGTATCAGTGCATTTTAAAAAGCTGTAATATGGCTGAACATTTA
AAACTATAACCTGTCACACACCAAAGATTAAGGTCCCAGGATGTACAAAACATGAAGTTCAAACGACACA
ACTGCAAAGCATATTTTATTTCACTTGGGAAAGAGGTTTCTCTCCAAAGATAGCTAAATATTACAGATGA
TGAAGACCTCTAACTTGTAGCCCAAGAGCTATAAACTACCATTAAATACCTGGTATAAACATAAACAAGT
AATTATCATTTGGATACAAGCATGTGTAAGCCAATAGAGCAATTATGTTTTAAGA

CNE9

> CNE9_Anolis
AAACGATTGAAATTAAAACTTCTTTTGTGATCAAAGGGGATTTTATTGCCTTTTGCTTAATTAAATGCCT
CAGAAGTGTGTGAAACATTGTTATAGTTGAAAAAATGTTACTTTGTAAATCGTAGGGACAAAAGGGCTCC
ACTGCAACTTTAAAGTTTCCGTGCACGTAAATGTCAATACAGTGCCTTCTCCATCATCAGCACTAAATTC
ACATTGATGCCTCTTTTCTTCTCTGTATTGATCCTTCCATGAGAACGTAGATTTGTATCTGTTAATTAAT
GCGTCCTGAAACAGCGTTTGTATTAATCAGGCAGATAAGAAAAATTGGATGCCTGCACCCTCATGACAAC
CGTAAAAGTGCTGGCCCTGATAAAAATCATGGACGGACCTCAATAAAAAAGTTCCTCCTTCCACTCAATA
AACTAATCATCCTGCTTTTAATTATTCGGCAGAAAGATGTGTGGAGATTGGAGAATGTGTAAATCTATTT
ACTAACAGCAGTGTCTGGGAGGGAGAATAGGGCTGTCACAGGACAGTGCTGCAGGCGACGTTCTCTGTCC
ATCTGCTGTAGCAGAAAAACTGGTTCTTGAGAGAGAAAGGGCAGAGAGGCTGGAGCTAATTGAAAATATG
CATTCCTTTTTGGACCCTTTCAAATTATTATTTCTGTTTCATTTCCTCCCATTTCCAACCAGACGTGTGG
CGTTCTCCATTGTCATGTTTCAGAGAAGCTTTCTTTACTTGTGT
> CNE9_ElephantShark
GAACTGGGCAGACTTTGGAGTTTCTGCGCACGTAAATGTCAATACATTGCCTTCTCCATCATCAGCACTA
AATTCACTTTGATGCCTCTTTTCTTCTCTTTATTGATCCCTTCATGAGAACGTAGATTTGTATCTGTTAA
TTAATGCATCCTGAAACAGCGTTTGTATTAATCAGTCAGATAAGAAAAATTGGATGCCTGCACCCACATG
ACAACCGTAAAAGTGCTGCCCTGATAAAATCATTGATAGGCCTCAATAAAAAAAGTTCCTCCTTTCACTC
AATAAACTAATCATCCTGCTTTTAATTATTAGGCCGAAGATGTGTGTAGATTGGGAATGTGTAAATCTAA
TTATTTACCAACAGCAGTGTCTGGTGGAGGGGGAGGGGGATAGAGAGAAAATAAAACTAGCTCTCTGCCG
TCGCACGAAGGTGCTGGCAGACACACATACAGGGCATCTGCTGCTTCTGGG
> CNE9_Zebrafish
ATGAAATAACAGATGAAAAGGAGGGAGGAAAAAAAAATGCATCTTTAAAGTTTGCCCTCGCGTAAATGTCA
ATAGAGAACCTTTTCCATCATCAGCAGTAAATTCACATTGATGGCTCTTTTCTTCTCTTTATCGATCCGG
CCATGAGAACGTAGATTTGTATCTGTTAATTAATGCGTCCTGAAACAGCATTTGTATTAATCAGGGAGAT
AAGAAAAATTGGATGCCTGCACTGGCATGACAACCGTAAAAGCGCGAGCCCCCTGATAAAATCATTGATG
GACCTCAATAAAGAGGGAGAAAAAAAAAAGTTCTTCCTTCCACTCAATAAACTAATCATCCGGCTTTTAA
TTATTCTGCGGAGAGATGTGTGGAGATTGGGGAATGTGTAAATCTAATTATTTACTAACAGCAGTGTCTG
AGAGGGAGGGCTGGGAGTGGCACAGGGGGAGCTCTAGGCGATGGGCTCCGTCCATCTGCCACTCCACACA
CCCGCCCCCTGCTCTCCGTTTG
> CNE9_Xenopus
TTATTATCTTTTCTTTTTTTTTTTTTTTAATTAAACCCCCCCCCCCAGTGAGGTTTTTTTATTTTTCGGAA
AGGTCCGCGGAGAGAAATTGTTCATTTGTAAATCACGGACAAAAGAGGCCGCGAAGAATCTCTAAAGTTT
CCGCGCACGTAAATGTCAATATAGTGTCTTCTCCATCATCAGCACTAAATTCACATTGATGCCTCTTTTC
TTCTACGTATTGATCCTTCCATGAGAACGTAGATTTGTATCTGTTAATTAATGCGTCCTGAAACAGCGTT
TGTATTAATCAGGCAGATAAGAAAAATTGGATGCCGGCACCCTCGTGACAGCCACAAAAGTGCTGGTCCT
GATAAAATCGTGGACGGACCTCAATAAAAAAAAAAAAAAAAAGTTCCTCCTTCCACTCAATAAACTAATCA
TCCCACTTTTAATTATGCCGTAGAAAGATGTGCGGAGATTGGAGAATGTGTAAATCTAATTATTTACTAA
CAGCAGTGTCTGGGAGGGAGAATTGGCTGTCACCGAAAGGTGCTGCGGGCTATATTCTCTGCCCATCTGC
TCCTGCAGAACAACAACAAAAAAAACAAAAACACCCGGGTTCTTGAGAGAAGTAGAGCATCTAAAGCTAA
TTGAA
> CNE9_Chicken
TGCAAGTTAAACTTTTTTTTTTTTTTTTAAATCAAATCTGATTTTATTGCTTTTTGCTTAATTAAACTCC
TCGGAAGAGTGTGAAACATTGTTATACCTGAAAAATTGTTAATTTGTAAATCACAGGGACAAAAGGGCTC
CACTGCAACTTTAAAGTTTCTATGCACGTAAATGTCAATAGAGTGCCTTCTCCATCATCAGCACTAAATT
CACATTGATGCCTCTTTTCTTCTCTGTATTGATCCTTCCATGAGAACGTAGATTTGTATCTGTTAATTAA
TGCGTCCTGAAACAGCGTTTGTATTAATCAGGCAGATAAGAAAAATTGGATGCCTGCACCCTCATGACAA
CCGTAAAAGTGCTGGCCCTGATAAAATCGTGGATGGACCTCAATAAAAAAGTTCCTCCTTCCACTCAATA
AACTAATCATCCTGCTTTTAATTATTCGGCAGAAAGATGTGTGGAGATTGGAGAATGTGTAAATCTATTT
ACTAACAGCAGTGTCTGGGAGGGAGAATAGGGCTGTCACAGGACGGTGCTGCAGGCTATGGTCTCTGTCC
ATCTGCTGCTGCAGAAAAACTGGTTCTTGAAAGACGGGAAGGAACAGAAACGTTGAAGCTAATTGAAATC
ACGAATTCTTCCCTATAACCTCTCCAACGATTATTTCTTCTCCATTTCTCTCTTATTTATTTTAATCACA
GATGTGTTATTTTCCATTGTCACCTCCTCGAGGGTTTTCTGGCACTGTTGGCTCGGGTTTTGTTGTTTTT
TT
> CNE9_Mouse
TTTGTGCTTAAAAATAAAGTACCCTTTCTTTACCTTTTCTTTTTCTTTATTTCATAGTATAAAGGGAAAG
AATAAGGTTTGCAAAACAGATGCCGCACAGGCACACACACAGAGCCATCCATACAGAGGAAACAAATTGC
AGAATCAACCTTTAAAAATGAAATTTTATTGCTTTTGCTTAATTAAACCCCAAAGAAATGTGGCAGACAT
TGTTGTTGTTGAAAAAAATGTTAATTTGTAAATCACAGGGACAAAGGGGCCCCGTGCAACTTTAAAGTTT
CCGCACACGTAAATGTCGATAGCGCGCCTTCTCCATCATCAGCACTAAATTCACATTGACGCCTCTTTTC
TTCTCGGTATTGATCCTTCCATGAGAACGTAGATTTGTATCTGTTAATTAATGCGTCCTGAAACAGCGTT
TGTATTAATCAGGCAGATAAGAAAAATTGGATGGCTGTACCCCTGTGACAACCGTAAAAGTGCTGGCCCT
GATAAAATCATGGATGGACCTCAATAAAAAAGTTCCTCCTTCCACTCAATAAACTAATCATCCTGCTTTT
AATTATTCGGCAGGAAGATGTGTGGAGATTGGAGGACGGTGTAAATCTATTTACTAACAGCAGTGTCTGG
GAGGGAGGACAGGGCTGTCTCAGGACGATGCTCCGGGCTATGAGGTCCCTCCATCTGCTTCGGCAACAGA

CTGCTGTTTGGGATCCTGAAGCGGAAGAATTAAACCAGGAAAATTAAAAAAAAAATTCCATTCCTGTGGC
TCAGAAGTTTAACAGTGATGATTGTTGTGGCAGGTGTTGTTGATTGAATTTGTAAAAGAAATTTCATTTT
ACAAATCTTTTTCTCTTCCAGTTCATCGTTGTGCTAGTGGTGGTGGTGG
> CNE9_Human
GCTGTTTCTTCCCCTTTTTTCCTTCTCTTTATTTCATAGAGAAGCAAAAGGAAAAAGTTAGGTTTGCAGG
AAGGATAAACACACACACATGCACACACATATACACACACACAACCATCAATATAGAAGAAACAAATTGC
AGGATCAACTTTAAAAATGAAATTTTATTGTTTTTTGTTCAATTAAACCCCAAAGAAATGTGCGAAATGT
TGTTATGGCTGTAACATTGTTAATTTGTAAATCACAGGGACAAAGGGGGCCCGGTGCAACTTTAAAGTTT
CCGTGCACGTAAATGTCGCTAGCGTGCCTTCTCCATCATCAGCACTAAATTCACATTGATGCCTCTTTTC
TTCTCCGTATTGATCCTTCCATGAGAACGTAGATTTGTATCTGTTAATTAATGTGTCCTGAAACAGCGTT
TGTATTAATCAGGCAGATAAGAAAAATTGGATGCCTGCACCCCCCTGACAACCGTAAAAGTGCTGGCCCT
GATAAAATCGTGGATGGACCTCAATAAAAAAGTTCCTCCTTCCACTCAATAAACTAATCATCCTGCTTTT
AATTATTCGGCAGAAAGATGTGTGGAGATTGGAGAATGTGTAAATCTATTTACTAACAGCAGTGTCTGGG
AGGGAGAATAGGGCTGTCACAGGAAGGTGCTGCGGGCTACACGCTCTGTCCATCTGCTGCTGCAGAGAAA
CTGCGACTTGGGAGCATGAAAAGGAACAAGAAAACTAAGGCAAATTGAAAAGACCCATTCATTTTTGCTC
CTAACTTTCCAGATGATTTTTTTTTTTTTGGTATTTTAATTTACACAAG
> CNE9_Lamprey
GATGTTGGCCACCGGCAGCCTTTTGCCGTTCTGCACACGTAAACATTGACGGGGGCGTCCTCTCCATCAT
CCAAAGTAAATTCACTCTGAAGGCCTATTCTTCCCCCCCGTATTGATCTCTTCATGTGAATGTAGATTTG
TATCTGTTAATTAATGCAGTGGGAAGCAGCCCTTGTGTTAATCAGCGAGATAAGAAAAATTGGACACTGG
GATGGGGCTGACAACAGTAAAAGTGTCAGGCTGATAACATCACCAATATCCCGTAGATAAAAAGTTCTGC
CAGCGCTCAATAGACCAATCATCCCGGTTTTAATTATTGGCCCGAAGATTCTGACCAACTGAAA

CNE10

> CNE10_Anolis
AATGAGCAGTGCAGCCTGTTCTTTCTGCAGGATCCTTGTCAAAGCACATGTAAGCAGCTCCATAAACAAC
TGAATGAAAGCTTTAGACATGTCTTTAAGACCCCCTTAAGAAAAGCTTGACTAATCCCCTTTGATGATTT
TATTAAGTACTTGGTGGTTGCTGGTGCCAACTGGCAAACCACTATGTTTTACCAACAGGCCAGGAAATCA
CTCCTTCACTGGCGCCTTATGCGAGGGTGGATAGTAACAGTCAGAGATAATAAATTAACAGCTGCTGTAA
ACAGGAGCCTGGAAAAGTATGGCACTGGAGACGTTTGCCCTCAGTGATCCTATTAGGCAGGAAGGTGGGG
AGCAAAACAAAAGCAAAAACTTAAACACATTAAGAAGCATTATTAAATTTCCAGCCTCTCTCAAAAATAA
TGGTGTGATTGCTATTTGCTTGGCACTTTAATGTGCCATCCTATTATTTCAGCCCTGCAGTTGAGGCAAG
AGCCATTACAGATGTAATTAGTGTATTTTCTGAAAAGGGTAGGCC
> CNE10_ElephantShark
CACACACAGTTTGAAAGCTTGGCTAATCCCCTTTGATGATTGTATTAAGTGCTTAAGGCGAGCTCTGGCA
ACTGGCATTCCACTCTGCGTCAAACCGACAGCCTTGTTTTTTCAAAAAAATCTCAAAACCTGTAAACTTG
CCCTGTTAACAAGATAGCAGATCTTAACAATGAGGAGGTAATAAATTAGTATGCTAGTTGTTATAAACAA
TACTGTGAAAGATACATAATGAGCTGTTTGCCATTAGTGGTTTAGTTAGAC
> CNE10_Zebrafish
CATGCCACCTTTCATTGAGGCCAGTTTGTTTGCAAGAATGCCGCAGTCTTGCATTAGACCCCCTTAAGAA
ATTCTTGGCTTAATCCCTTTTGATGATTTTATTAAGTAGTAGAAGATGGCCCTGCCAACTGGCATTGGGC
ACACTTGCACCAGCAGGCCAGGAGAGGCACCAACTGCTCACCAGCTCTTAGTCTGCCTCTAATGAGATCA
GAGATGTTAACAGTTTGGGATAATAAATTAGCAGTTGGTATAAACAATAGCCCCAAACCTATACCTAATG
AGCTCCTTGCCATTAGTGATTAACTGGCAAAAAAAAAGAGATTTCAAAATAAAAGAAA
> CNE10_Xenopus
TGCCACGGCCAAGTCAATGTAAAGCCACAGACCCCCTTAAGAAAAGCTGCACTAATCCCCTTTGATGATT
TTATTATGTCCGTGTCATGGT
> CNE10_Chicken
ATGGCAAAAAGGATGTTCTTGCAGCAGGAGCCCTGTCAGAGAACGTGTAAGCAGCTCCATAAACAACTAA
ATGAAAGCCTCAGACATGCCTTCAGACCCTTTAAGAAAGGCTTGACTAATCCCCTTTGATGATTTTATTA
AGTAGTCGTGGTTGCTGGTGCCAACTGGCAAACTCATGCAGCTTACCAACAGGCCAGGAAATCACATCCT
CTCCCCTCTTGTTCCATAGCTCCTAATGTGATTGGGATATTAGCAGTTTGATATCAGAAATTAACAGGTA
CTGTAAACAAGAGGCTGAAAAGGTAAGGCAGTAAAGATGTTAGTCCCACATGATCCTATTAGGAAAGACA
GCAGGGAAAAAAAGAAGGAAAAAAAAAAAAAAAAGGAAAGCAAAAGTTAAATTCATTAAGAGGCATTTATT
AAATTTTTGCTTGTCCTCAGAAAAGTGACGGTGTGACTGCTGTTCAGTTGGCACCGTAATGTGGCATTCT
GCTCCTCCACCAAGTGCAGCAGAGGCAGGAGCTGTTGCAGAAGGAATTAGCATCTTTTCTGAGGAGTGGG
TTACTTC
> CNE10_Human
TCTCTAAGAGGATATTTATTAATTTAACCCCAAATGCAATGACAAAAGCAGAATAAGAAACAAGACTTAC
ACAGCCAGTCCAAAAGAAATCCTGTCAAATAACATGTAAGCACCATGATAAACAACGAAAGAAATGCATT
TTACACATATCTTAAAGATTCCCTTAAGAAAAGCTTGACTCATCTCTTCTGATGATTTTATTAAGCACTT
ATAATTAATGGTACCAAATGGCAAACCACTGTTTTTCACCAATAGGTCAGGAAATCCTTTCCTTTTCTTT
ACTGTGCTGTAGTTTCTAATATCATATGGGATACCCACAATTTGATATGATTGAAAAGGACAGCAACAGT
GACCATAAGCAGCAGCCTAAAAAGGTTGGGCATCAGTGGCTACTTATGATTTGGATAATAATGTCTAGAA
AGGGAGTAAAAATAGATTTCTTAAGTTGCATTATTAAAAATG

el161

> el161_Anolis
TTCTAATTTGCTTAAAAGAAGTCAGAATTTTTTTCGGAAGGTATTAAAAGTAAAGATGCTTTCCAATAAA
CATAGATGTTGTTCTGCTTAAATCCATTTCATAATCCAGATAAGTCTAATCATTTCCCTAAAACCCAGTA
GAATGGATGGGACCATGACTAAATGTAATATAAGATCCACTTCAATCCAGCTACTAAACACAGTGAACTC
AATAAGTAAGGTGAACTCAGGTTTTATCAGGGAAGGGAGCAAATTACTGTTTAATCCTACAGGAACTCTT
CTTCATCATACAAATTTGATTTTATTGTAGCTGTTTAACTGACAGATACAACTTATTCAGGAGGGGTTAA
GAAATCAAGGGAGGATTCTTCTCTGCATTTGAACTTAAAAGTGCTGAAACAGTGAACCACTGTATGATAA

GTAAAAAATCGTTTGTTCTTTGTTGTTTTTTCTTTTGTGCTTCCCTAATGCTTTATTGTGGTCTTAAGTC
CCTTTTAGCATTTGCATAAAGTTCATGATAGATCCCCCTTTAATGACGTGCCGATCATCAGATACCTGTG
TGTCAATAACATGCTCTGATGCTATGTACCATTGACAGTCACACCGTGCCCCTCCATCTGCTTTTGTTTT
GACCCTATCTTTGAACTTTATCTTGGTTGCTGGCCAGTAGTTTTCCCTTTAATTACAAGAAGGAAACTGT
CAATAAAATCTGTTAAATGGGATGCAATGAGTGGCTTGAATGGGCGGACGGCTATTTGTTCAAATTCTGC
AGCATTCAAGGTATTGACAGTTTGTTTTCTGGGGCCATATGTGACGATCAATCTCAGGCTGGGCTCAGCC
GCGCTGAAAAATGAAAAACCAGATTGCTTTTGCTTACTTGTGGATGACGACTTGTGATTTGGTACGGTCC
TAGTGAGATGAGACCTTCTGCCCAAATTGCCCCCCCGAGAGCCAGGGTGCGTGACTGTTTTTAGAAATAA
TTTTTAATTGATTTTGTAATTAACACTTCATCTACAATATTGATGCACAGATCTTCAGACGGATAGGAGG
AAAATTGTTTTCAACAATGAAGTTGTACTTTCCTATTTGTCTTCATGATGGGATCTAGCAGTTTGAATAT
TTAATTGGGATTTAATTTTTTTTTTAATTAGGAAGCAAAACCAAGAAAATATA
> el161_ElephantShark
CAACTGGGAATGAATCATGAATAAAATCGTTTGTGCTTTGTTGTTTTTTCCCTTCTCCCCAATGCTTTAT
CGCAGTCTTTAGACACTTTAGCATTTGCATAAAGTTCATGATAGATCCCTCTTTAATAACATACTGATCG
TTAGCTACCTGTGTGTCAATAACAGCTCTGATGTTGTGTACCGTTGACAATCACGCCATGTCCCTCCATT
TCCCCCTGCCTCCTTTTGTTTTGACCTAATCTTTGAACTTTATCTTGGTTGCCGGCCAGTAGTTTTCTCT
TTAATTACAAGAAGGAAACTCAATAAAATCTGTTAAATGGAATGCAATGAGTAGCTGAATGGGAGGACAG
CCATTTGTTCAAATTCTGCAGCACTCAAGGTATTGACAGTTTGTTTTCTGAGGCCATATGTGTTGATCAA
TCTCAGGCTGGGCTCAGCCACGCTGAAAAATGAAAAACCAGATTGCTTTTGCTTATTTTCGGATGACAGC
TCTTGATTTGGTGGGTCCGAGATGAACCTTCTGCCAAATTGTACCCCTGAGAATCAAAGCGTGACTGTTT
TGAAAATTATTTTTTAATTGATTTTGTAATTAACAGTTCATCTCGTATATTGATGTGCAAATTCCTAGAT
TGATGGGAGAGAAATTGTTTCAACAAAAAGTTGCACTTTTTTCATTTGTCTCAGCAACTGCTGTTGGCAC
T
> el161_Zebrafish
ATTAGCATAAGTCTCTGCTCAGATGCCTCTTAAGTAAGGAGCCCATCACTATGCACCTGCATGTCAATAA
CAGGCTCTGATGTTGTCCACCATCGACAGTCACACCATGGCCCTCTGGCTCCCTTTGTGTTGACCCCATC
TTTGAACTTTATCTTGGTTGCTGGCCAGTAGTTTTCCCTTTAATTACAAGAAGGAAACTGTCAATAAAAT
CTGTTAAATGGGACGCAATGAGCAGCTTGAATGGGAGGGCGGCTATTTGTTCAAATTCTCCAGCGCTCAA
GGTATTGACAGTTTGTTTTCTGGGGCCATATGTGACGATCAATCTCCGACTGGGCCTGGCCATGCTGAAA
AATGAAAAAGCAGATTGCTCTTGCTCGCTTGTGGATGACGGCTAGCTGCATTGGCCCTAGCCTGGAGACA
CTGGGGGCCGTTCTGCCCAAAGTGCACCCCTGAGAGAACGAGCAAGCGCGTGACTCTTTTGATAAATTCT
TTTTTAATTGATTGCACTATTAGCAGTTCATCTCATCTTGTTGACGCACACGTCGGCGTGCCAAAAGC
> el161_Xenopus
GGTTTTTCGTTAAGGGTTAAGAAGAGTTTAGCCATCATGAGGGCATAAATGTGCTGAAGCAGTGACCGGG
ATCAGATAAGTAAAAATCGTTTGGGCTTTGTTTTCCCTTTGTGTCACCTTAATGCTTTTCCTGTGGTCTT
AAGTCCCTTTTAGCATTTGCATATAGTTGATGATAGATCAGTGCTTAATAACATGCTGATCATTAGATAC
CTGTATGTCAATAACACGCTCCCATGCTATGTGCCATTGACAGTCACACCGTGCCCCTCCATCTGCTTTT
GTTTTGACCCTTTCTTTGAACTTTATCTTGGTGGCTGGCCAGTCATTTTCCCTTTAATTACAAGAAGGAA
ACTGTCAATAAAATCTGTTAAATGGGATGCAATGAGTGGCTTGAATGGGCAGACGGCTATTTGTTCAAAT
TCTGGAGCATTCAAGGTATTGACAGTTTGTTTTCTGGGGCCATATGTGGCGATCAATCTCAGGCTGGGCT
CAGCCGCGCTGAAAAATGAAAAACCAGATTGCTTTTGCCGACTTGTGGATGACGACTTGTGATTTGGTGC
AACCCTACAGAGCGGAGACCTTCTGCCCAAATTGCCCCCCAGAGAGATGGAAATGTGTGACGGTTTATAG
AAATATTGTTTTTAATTGATTTTGTGATTAACAGTTCATCTATAGCATTGTCGATGGTCTTTGATAGAAT
CAGTTTTTTACAGTGCTTTGT
> el161_Chicken
GTGATTTGGAGAGTATTTTACAGGCAAAGATCCTTACAAATGAATATGGATGCTCTTCTCCTAAATCCAT
TCCATAATCCAGATGAGTCTAATCATCGTAGTTAAAATGAACAGAAAGGATTCATGAGGATATGCTATAT
CCGTTCTCTTTAAATGCAGCTACTGATTTATAAGATAGAGTGAATACAGGGTATGGGAGGGGGGAGGAAA
TTCTTTATTAATCTGACGGGGACCCCACCTTACCTTTTGGATCCTCTGCATTTGTGGTAACGCTTCTCCA
AAGATTACTGTTACCTGTGCAGAAAGGGTTAAAAATCTCAGAAAGATTTTTTTTTCCTGCATTTGAATTT
AAAAGTGCTGAAGCAGTGAATACTGCCAGATAAGTAAAAATCGTTTTTGCTTTGTTGTTTTTCCTTTTGT
GTCTCCTTAATGCTTTATTGTGGTCTTAAGTCCCTTTTAGCATTTGCATAAAGTTCACGATAGATCCCTC
TTTAATGACGTGCCGATCATTAGATACCTGTGTGTCAATAACATGCTCTGATGCTATGTTCCATTGACAG
TCACACCGTGCCCCTCCATCTGCTTTTGTTTTGACCCTATCTTTGAACTTTATCTTGGTTGCTGGCCAGT
AGTTTTCCCTTTAATTACAAGAAGGAAACTGTCAATAAAATCTGTTAAATGGGATGCAATGAGTGGCTTG
AATGGGCGGACGGCTATTTGTTCAAATTCTGCAGCATTCAAGGTATTGACAGTTTGTTTTCTGGGGCCAT
ATGTGACGATCAATCTCAGGCTGGGCTCAGCCGCGCTGAAAAATGAAAAACCAGATTGCTTTTGCTTACT
TGTGGATGACGACTTGTGATTTGGCGCGGTCCTAGTGAGATGAGACCTTCTGCCCAAATTGCCCCCCTGA
GAGCCAGGACGCGTGACTGTTTTTAGAAATAATTTTTTAATTGATTTTGTAATTAACAGTTCATCTACAA
TATTGATGCATGAATCCTCAGATTGATAGGAGGGAAATTGTTTTCAACAATGAAGTTGTACTTTCCTATT
TGTCTTTGTAATGTGATTTAGCACTCCGCATTTTTAATTGGAAATATAATTCAAAAACATGCAAAGCCAA
AATGCTTGTTAGTTTCTTTGTATTC
> el161_Mouse
AATTCTAGGAATACGTATGCCTAGCTGGGACAGGAGTGGGCTAAAATAGGGGCATACTCTGGTAATTCAA
AATGGTTGGTATTTTGTTATATTTCCCCTTTGTGCCTTCTCTAAGGCTAGATTGTAGTATTAAGTCTCTT
TTAACATTTGCATAAAGTTCAGGATAGATCCCTCCTAATGACGAGCTGATCATCAGATACCTGCGTGTCA
ATAACGGCTCTGAAACCATGTACCATTGACAGTCACACGACACTCGTTCCAGCTGCTTTTGTTTTGACCC
TATCTTTGAACTTTATCTTGGTTGCCGGCCAGTAGTTTTCCCCTTAATTACAAGAAGGAAACTGTCAATA
AAATCTGTTAAATGGGATGCAATGAGTGGCTTGAATGGGCGGATGGCTATTTGTTCAAATTCTGCAGTAT
TCAAGGTATTGACAGTTTGTTTTCTGGGGCCATATGTGACGATCAATCTCAGGCTGGGCTCAGCCGTGCT
GAAAAATGAAAAACCAGATTGCTTTTGCTTACTTGTGGATGACGACTTTGTGATTTGGCGCGGTCTGGGC
AAGATGAGACCTTCTGCCCAAATTGCCCCCATGAGAGCCAGGGATGCGTGACTGTTTTTAGAAATAATTT
TTAATTGATTTTGTAATTAACAGTTCATCTAAAATATTGATGCATGAATCCTTGGACTGATGGAAGGGAA
ATTGTTTTCAACAATGAAGTTGTACCTTCCTATTTGTCTTTATAATGGGTGTTAGCATTTCACAGTTTTA
AATGTGGATATAACCCACTTATTACCACACAATTCACCCTACTTTCCCTCACTTTCCTGGGAACTTAAAT
TGTGAGTGAGTGTGTGTGTGTGT
> el161_Human

GCTTTGAGTTTTGGTTATCAAAGAACAGTTTTCTTACATTCTGGAGTTCTTTAGAAGTCAAATTCTGCCT
ATGCAGTAAGGGTTAAGAATTATGGGAATAACTGGTCTTTACATTCGAACTGAAACATGCTAAAATAGCA
ACCTCTAATAAGTAAAAATCGTTGGTGCTTTGTTGTTTTTCCCTTTGTGCCTCTCTTAATGCTTTATTAT
GGTCTTAAGTCTCTTTTAGCATTTGCATAAAGTTCAGGATAGATCCCTCCTTAATGACGTGCCGATCATT
AGATACCTGCGTGTCAATAACGTGCTCTGATGCTATGTACCACTGACAGTCACACCGTGCCCCTCCATCT
GCTTTTGTTTTGACCCTATCTTTGAACTTTATCTTGGTTGCCGGCCAGTAGTTTTCCCTTTAATTACAAG
AAGGAAACTGTCAATAAAATCTGTTAAATGGGATGCAATGAGTGGCTTGAATGGGCGGACGGCTATTTGT
TCAAATTCTGCAGCATTCAAGGTATTGACAGTTTGTTTTCTGGGGCCATATGTGACGATCAATCTCAGGC
TGGGCTCAGCCGCGCTGAAAAATGAAAAACCAGATTGCTTTTGCTTACTTGTGGATGACGACTTTGTGAT
TTGGCGCGGTCCTAGTGAGATGAGACCTTCTGCCCAAATTGCCCCCATGAGAGCCAGGGACGCGTGACTG
TTTTTAGAAATAATTTTTAATTGATTTTGTAATTAACAGTTCATCTACAATATTGATGCATGAATCCTTA
GACTGATAGGAGGGAAATTGTTTTCAACAATGGAGTTGTACTTTCCTATTTGTCTTCATAATGCAGTTTA
GCATTTCACAGTTTTAATTGTGAATATAACCCTCTTCCTCACCACACCATGCACCTCTCTTCTTCTACGC
TCCTGTGAAATTATAAAATATGATTTCTTATGACAAATGACATTGTTATTATGAACCCTGA
> el161_Lamprey
GCGCACAGGCCAGCGTCTTGGTCTGAGGCCAGCTGGCTTTTCTCGTTAATTACAACGGGGGAAAATGTCA
ATAAAATGTGTTAAATGAAATGTAATGAGTGGCCGTATGGGAGGGCAGTCATTTGTTCGCATTGTTCGCT
CTTCAAGGTATTGACAGTTTGTTTTGGGAGCAGCGCGTTGATCAATGGTGGCTGCGAATCGGTCAAACTG
AAAAATGAAAAAGCAGATTGGCTTTGCTCAGTTTCAGCGGAGGCATTAGAGGCTGCGGTAAGCCACAGAC
GAGGCTTTTGTCTAATCGTGCCCCCGAGAACTAAAACGTGACTCCTTTCGAAATTATTTTTAATTGATTT
TGTAATTAACGGGTTCAGCCCTTCCATCGACGTAGGGCCTGTTGACTGATGGGAAGGAAATTGTTTCACC
AAAGTAACACCGTGCTCATTTGTCTTCTCAAGTAGCTCTGATGT

Lamprey ERR8t12 cloned sequence

>ERR8t12_seq
GCCCACACACCGCCTAATATTTACTGTGAAATATGAGCCGTCGCCATCTGTAATGGCTGTAAAATTTCATAATTACATGTCCTTTA
TTGCATTGCGTAATGATCCTGAAGCGGCGCAAAAGAATAATTAATTAGGAGATTATGAGCCGTAGATTCG
GAGAATTAAGGGAATTATTGGGGGGGGGGGAGGGAGGAGCCTACACACTCTGAGCCCCTCCGCGGCTCTG
CTTTACTGCGTGTGCAGCCCAAAAACACGAAGGCGGCTCGGAATGTCCCAGCGCGCGTCGCCGCGCAACA
GAGGGCCATTATTATCCAAGCGCTTTCCGTGTTCGACAAACATATTTAGCAAAAACACGGCACGCTAAGT
GGGTAATATTTGTTGAAGAAATATCGTCGAGTAGTTTTGAGGATTTCTTAGTGTGTGTGTGTTTATAAAT
GCGTTGGGCTTTATTGAAGATGTTTGCATGGTTCAGTTTAAACAGGCATGGGTCTCCCCTCACAAGTCCT
GGAATGAAAAAGTGTGAATAATTGTATTATTCTGACCTGAAGCAGATTAGGCACTTGGCTACTGATTTCT
GTAGCATTTGCCTGCTCATGTTGCCACAATTGGCTATTGAGTCAATTAGTGAGATGCATTTATTGTGTCT
AACAAGGAAATCAATATTAAAATTGGTTGATTTACTGGTGCTGTAATTATGATTCCATGTTGATGGAGAG
CGCAGGATAGTGTGATTATCGCCCTTATTTTGGTACAACAAGACATTTATTGTCTCTGTGGGACCGTGTT
CTATTTTGTCTTCAAAACACGAGTTAATTGCCTCCTGTGGACGTGCTGTGTGGCACCGTTTGCAACACGC
AGCCCTTCCTCGTGGACGACACTTGGAGAGGAAAATGGCTCACGTGGGTCCCTGCAGGCTGCCGCTAAGG
CTGCCAGTGGCATGCGCCGTCCCGCCGATGATGGCCAATGGCGGGACAGCGCATGCCACTGGCGGGGCCG
AGTGGCGGGCCAGGTGCCCCATGGGCACCGTGCCACTCTGCTGCCCTCGTCCTGTTCCCCTGCTCTGAGC
GAAGGTCCAAGTGCCCCACTGCGCCTTGCGCCACTCCCCGCTGCCCTCTGCGCAGCCCAGGATTCTTGCG
GCAACGCTCGGGATTGGCTGATTAAAATGTAAATGTTGATATTTGGGCACATAAAAGTCGCTTCCCCGTG
TGCCACTTACCGCCCTCCCTGAACGCTCACAAGCTGCGATTCGCGGTGCTTCAAAATTAGTGCCAGCGTA
GCGATCGCCGCCAAAACCCAAACTCTGTTTCTCCCAGCAATTCTGGCCTCTAATTAGGCAAACAGGCGAA
CGATCCAAGCATCCTGAACAGCTGCTCCAGTATATTTAGAATTAATCTCGGCTCAGAATAGTCTAATTTA
TGCAATGGTCTGGAATTACGGCATGCTTAATTAGGCTCATTGAAGCCATGTGACTGAGGCTGCCCCGAGA
GAAGAGCTGAGGGCTCCGAATGTGGATTACATTGTTCCGAGAGCAGACCTGAGCCACGCTAATCAGCCCC
CACACCTGTCTGGATTAAAGGTCAGCTGTGGTTAGCACAAGAGACTGGGATTTAAACTGGAAGCTTATCC
ACTAGGATTTGTAGTATTCATGTAGCGGCTAACACAGCGATCAAACTTGTCTTTTTTTGGGGACTCAGCC
TTACACAAGACTCTCGTGTAATTGTCCCGACATCCATCTGAGTCTCAAGTATTACACGTGAATCCTTTAT
TCTTGACCAATTGAATGGGTAAAATGTGAAGCAGGGTTTTAATGGTATTAGTGAGATTTAGGATAAAACT
GAATAAAAGATTCACATAATAATGTAGTTCATGTGACGCTGTAAAGATATATTTTTTTGCATCCACTGTT
TGCGTTTCCGCAATTGATAATAATGAAATGTAACACTTCTTGTGGCTGAGAATTATCTGGCGTTAAATGT
TGCCAATCTTGGCTGGAAGTCCTATGATGTTGACAAATTTGGCAAGTTGTATCATCCATCTGCTAGGTAT
TAGGGGATTATGATCAGTAGTTTCCGTGATGCAGGAAATGAAGCGGCACAGCCCAGGCAACTTGCCATGG
AGTAAAGCCTACCTTTGCTGCAGGACCCACCACCACCGCTGATATGGGGGCATCAGCAGAGTCGTTTTAA
AGCCAACCAATGCTTTGTGCAGTTTGTGTGGAATAAGCACTGTTTCCTTTATACGCTGCTAATTTAAATT
GCTTACCGTTCAGCCTCAAGCTATTTACATTCCATTCACTGAAACAAAATGATCTTTTCAATCAGATGTT
TATTGTGAGGTTTTTTTCTTCTTCTTCCAAAACCACAATCAGGAGGCCCCAAGACCTCCACAGATAGCAT
CTTTTCAAGTGACCAGAAATAGAGGGACCCATTATCACGGTAACCTCCAGCGGCACCAGGCATCCTTCTT
TCTCGCGCTCCCATTCAGCGCTCGGCCCGTCACTCACGGCTGCCGCCGTGCCCCCCCCCCAAATGTGGC
GCGTGGGGGGGGGGGCGCTCCGGCACCGCCACGGCGATGTTGGCCACCGGCAGCCTTTTGCCGTTCTGCA
CACGTAAACATTGACGGGGGCGTCCTCTCCATCATCCAAAGTAAATTCACTCTGAAGGCCTATTCTTCCC
CCCCGTATTGATCTCTTCATGTGAATGTAGATTTGTATCTGTTAATTAATGCAGTGGGAAGCAGCCCTTG
TGTTAATCAGCGAGATAAGAAAAATTGGACACTGGGATGGGGCTGACAACAGTAAAAGTGTCAGGCTGAT
AACATCACCAATATCCCGTAGATAAAAAGTTCTGCCAGCGCTCAATAGACCAATCATCCCGGTTTTAATT
ATTGGCCCGAAGATTCTGACCAACTGAAAGCGTATAAATCCAATCGTTTGCCGCTGGCAGTTTTGAGTGT
GCTGAGCATTCCTCTCGGATGC

5. NCBI Sequence IDs for sequences used in molecular phylogenetic analysis

FAMILY	PROTEIN NAME	NCBI ACCESSION
	ERR_lottia	AGG68283.1
	ERR_capitella	ELT99464
	ERR_lingula	XP_023932889
	ERR_Priapulus	XP_014672486
	ERR-like_Priapulus	XP_014680032
	ERR_Saccoglossus	XP_006824850
	ERR_Strongylocentrotus	XP_011669460
	ERR_Branchiostoma	AAU88062
	ERR_Ciona	NP_001071700
	ERRB_Lepisosteus	XP_015206123
	ERRG_Lepisosteus	XP_015201956
	ERRGr_Lepisosteus	XP_006627644
	ERRA_Oryzias	NP_001098387_1
	ERRB1_Oryzias	NP_001098388_1
	ERRB2_Oryzias	NP_001156563_1
	ERRG1_Oryzias	NP_001098389_2
	ERRG2_Oryzias	NP_001156564_1
	ERRGr_Oryzias	XP_011480485_1
ERR	ERRA_Danio	NP_998120_1
	ERRB_Danio	NP_001311468_1
	ERRGa_Danio	NP_998119_1
	ERRGb_Danio	NP_001122150_1
	ERRD_Danio	XP_001921093_3
	ERRA_Xenopus	NP_001072756_1
	ERRB_Xenopus	XP_002938647_1
	ERRG_Xenopus	NP_001093680_1
	ERRGl_Xenopus	XP_002938860_2
	ERRA_Chrysemys	XP_005305569_1
	ERRB_Chrysemys	XP_008163110_1
	ERRG_Chrysemys	XP_005288793_1
	ERRA_Anolis	XP_003230133_2
	ERRB_Anolis	XP_008119299_1
	ERRG_Anolis	XP_016846156_1
	ERRB_Gallus	XP_015143195_1
	ERRG_Gallus	NP_001007082_1
	ERRA_Homo	NP_001269379_1
	ERRB_Homo	NP_004443_3

	ERRG_Homo	NP_001429_2
	ERR_Mizuhopecten	BAN84542.1
	ER_Mizuhopecten	OWF40232.1
	ER_Octopus	ABG00286.1
	ERR_Octopus	XP_014789866.1
	ERR_Drosophila	NP_729340.1
	ERR_Apis	NP_001155988.1
ER	ER_Capitella	ELU03759.1
	ER_Lingula	XP_013404577_1
	ER-like_priapulus	XP_014668376_1
	ER_Saccoglossus	XP_006824830_1
	ER_Branchiostoma	ACF16007.1
	ER1_Lepisosteus	XP_006625908_1
	ER2_Lepisosteus	XP_006632252_1
	ER1_Oryzias	XP_004083548_1
	ER2_1_Oryzias	NP_001098172_1
	ER2_2_Oryzias	NP_001121984_1
	ER1_Danio	NP_694491_1
	ER2a_Danio	NP_851297_1
	ER2b_Danio	NP_777287_2
	ER1_Xenopus	NP_988866_1
	ER2_Xenopus	NP_001035101_1
	ER1_Chrysemys	NP_001269175_1
	ER2_Chrysemys	XP_005285947_1
	ER1_Anolis	NP_001277446_1
	ER2_Anolis	XP_016846689_1
	ER1_Gallus	NP_990514_1
	ER2_Gallus	NP_990125_1
	ER1_Homo	NP_001278159_1
	ER2_Homo	NP_001428_1
	ER_Lottia	XP_009064842_1
	ER_like_branchiostoma	ACB10649
AR	AR_Danio	NP_001076592.1
	AR_Gallus	NP_001035179.1
	AR_Anolis	XP_008118585.1
	AR_Xenopus	XP_002941888.2
	AR_Chrysemys	XP_005279584.1
	AR_Callorhinchus	XP_007892478.1
	AR_Homo	NP_000035.2
RXR	RXRA_Homo	NP_002948.1

RXRB_Homo	NP_001257330.1
RXRG_Homo	NP_008848.1
USP_Drosophila	NP_001259168.1
USP_Apis	NP_001011634.1
RXR_Mizuhopecten	XP_021373595.1
RXR_Crassostrea	XP_011434500.1
RXR_Strongylocentrotus	XP_011661795.1
RXR_Ciona	NP_001071809.1
RXR_Branchiostoma	XP_002609360.1
RXRA_Callorhinchus	XP_007901074.1
RXRG_Callorhinchus	XP_007901150.1
RXRG_Xenopus	XP_004913829.2
RXRB_Xenopus	NP_001015937.1
RXRA_Xenopus	XP_012824678.1
RXRG_Gallus	NP_990625.1
RXRA_Gallus	XP_003642339.2
